# Dithienoarsinines: stable and planar π-extended arsabenzenes†

**DOI:** 10.1039/d4sc06590e

**Published:** 2024-11-19

**Authors:** Akifumi Sumida, Akinori Saeki, Kyohei Matsuo, Kensuke Naka, Hiroaki Imoto

**Affiliations:** a Faculty of Molecular Chemistry and Engineering, Graduate School of Science and Technology, Kyoto Institute of Technology Goshokaido-cho, Matsugasaki, Sakyo-ku Kyoto 606-0962 Japan himoto@kit.ac.jp; b Department of Applied Chemistry, Graduate School of Engineering, Osaka University 2-1 Yamadaoka Suita Osaka 565-0871 Japan; c Innovative Catalysis Science Division, Institute for Open and Transdisciplinary Research Initiatives (ICS-OTRI), Osaka University 1-1 Yamadaoka Suita Osaka 565-0871 Japan; d Institute for Chemical Research, Kyoto University Gokasho Uji Kyoto 611-0011 Japan; e Materials Innovation Lab, Kyoto Institute of Technology Goshokaido-cho, Matsugasaki, Sakyo-ku Kyoto 606-0962 Japan; f Fusion Oriented Research for Disruptive Science and Technology (FOREST), Japan Science and Technology Corporation (JST) Honcho 4-1-8 Kawaguchi Saitama 332-0012 Japan

## Abstract

Stable planar dithienoarsinines were synthesized and structurally characterized. These compounds exhibit monomeric structures in the solution and solid states, avoiding dimerization, even in the absence of steric protection. They exhibited high global aromaticity with 14 or 22π-electron systems. In the solid state, intermolecular interactions through arsenic atoms were observed, and As⋯As interactions resulted in aggregation-induced emission enhancement properties with a significant bathochromic shift. The W(CO)_5_ complex displayed a significantly distorted coordination geometry owing to arsenic cooperative stacking and hydrogen interactions, resulting in a 1D alignment of the complex. Additionally, despite their aromatic nature, dithienoarsinines undergo reactions with alkynes or benzynes to form the corresponding [4 + 2] cycloadducts. Oxygen molecules oxidize the *p*-position of arsinine, leading to the formation of σ-dimerized compounds while retaining the aromaticity of the arsinine ring. In contrast, oxygen attacks the phosphorus atom in phosphinine, resulting in the formation of phosphinic acid with a loss of aromaticity.

## Abstract 2

Dithienoarsinines, the first π-extended arsabenzenes, exhibit high stability and planarity. Their structure, optical/electronic properties, reactivity, and coordination behavior are comprehensively studied, highlighting the features of arsenic.

## Graphical abstract

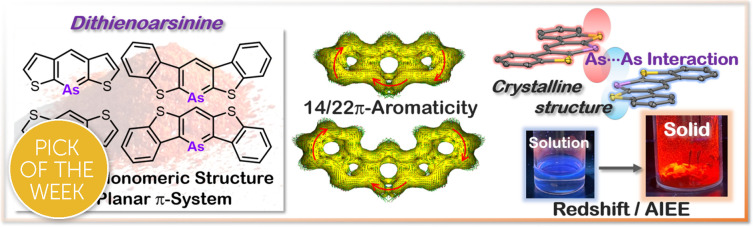

## Introduction

**Fig. 1:**
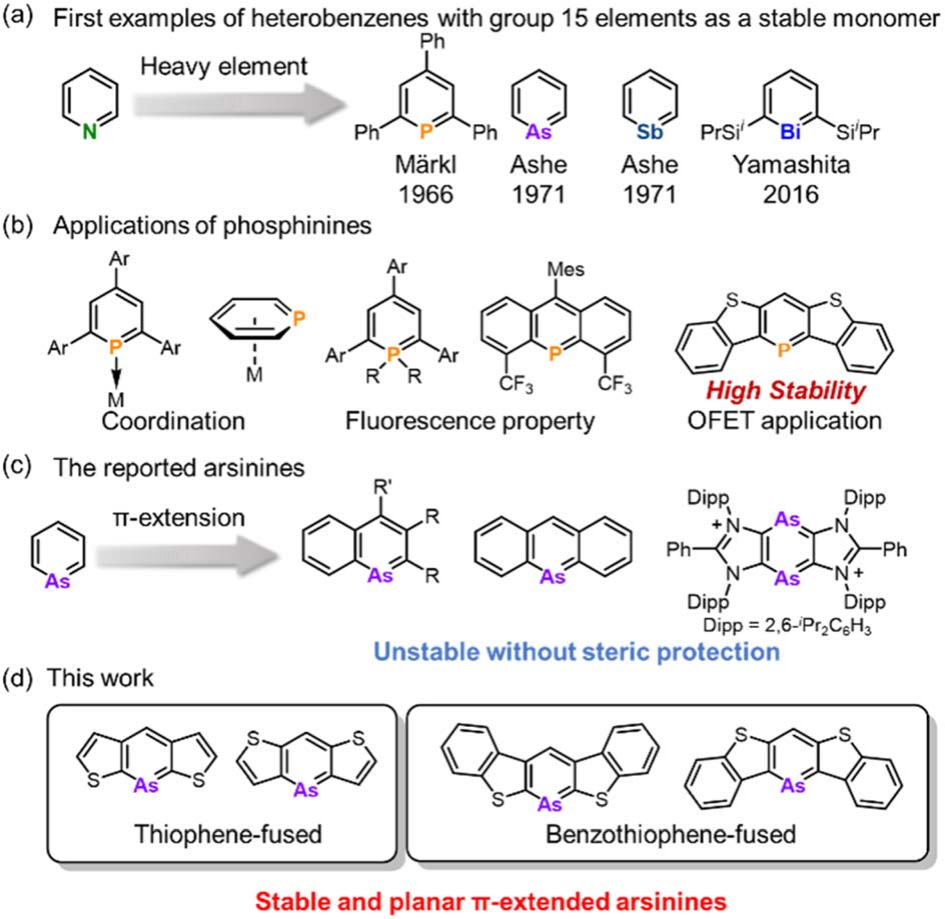
(a) First examples of the isolation of heterobenzenes embedded with pnictogens as a stable monomer. (b) The applications of phosphinines and π-extended phosphinines. (c) The examples of arsinine derivatives with π-extension. (d) Thiophene- and benzo[*b*]thiophene-fused arsinines.

Since the 1960s, heterobenzenes have attracted significant attention and numerous heteroatoms have been incorporated into benzene frameworks to investigate their structure, aromaticity, and reactivity.^1^ Among these, group 15 heterobenzenes, heavier analogs of pyridine, are noteworthy because of their lone pair of electrons, which confer Lewis basicity, and their dicoordinated structure, which maintains planarity (Fig. 1a). Phosphinine motifs are widely utilized as ligands in transition-metal complexes and as conjugated units in organic semiconductors (Fig. 1b).^2^ Transition metal complexes with phosphinine ligands have been extensively studied because of their ability to coordinate through various modes. Phosphinine units serve as π-acceptors due to the symmetry and lower energy level of their LUMO, as well as σ-donors through their lone pairs. This dual functionality led to the development of unique metal complexes using monodentate and multidentate phosphinine ligands.^2*a*–*c*,3^ In the realm of luminescent materials, λ^3^- and λ^5^-phosphinines have been extensively explored,^4^ and recently, blue OLED materials based on λ^5^-phosphinine have been developed.^5^ Additionally, π-extended phosphinines have gained interest for their structure, reactivity, and electronic properties as heavier aromatic molecules and their potential applications in optoelectronics and semiconductors. Consequently, various phosphinine derivatives have been synthesized and investigated, including 1-phosphanaphthalene,^6^ 2-phosphanaphthalene,^7^ 5-phosphaphenanthrene,^8^ phosphaanthracene,^9^ 1,4-diphosphinine,^10^ and dithieno[3,2-*b*:4,5-*b*′]phosphinine.^11^

In contrast to the rich chemistry of phosphinines, arsinines, which are heavier analogs, have been markedly less studied, and their synthesis remains challenging (Fig. 1c). This is because of the potential instability caused by the poor overlap between the arsenic and carbon orbitals.^12^ Conversely, utilizing the large arsenic π-orbital could enhance the intermolecular interaction to construct the unique molecular arrangement. Furthermore, a narrower bandgap can be utilized for luminescent materials or unique reactivities. The parent arsinine was first synthesized by Ashe in 1971,^13^ marking the commencement of its chemical investigation, with various derivatives emerging in the 1970s.^14^ Despite these developments, its π-extended derivatives are rare. In 1969, Jutzi and Bickelhaupt reported the synthesis of 9-arsaanthracene,^15^ although it was detected only by mass spectrometry and UV-vis absorption spectroscopy and was isolated as the [4 + 2] adduct with maleic anhydride. In 2001, Ashe described 1-arsanaphthalene bearing a trimethylsilyl group at the 2-position.^16^ Nevertheless, these arsinines are prone to rapid dimerization at ambient temperatures *via* [2 + 2] or [4 + 2] cycloaddition, which hinders detailed structural and property insights. More recently, Dostál synthesized 1-arsanaphthalene from 2,1-benzoazaarsole by incorporating bulky diisopropylphenyl (Dipp) groups.^17^ Ghadwal investigated 1,2-diarsinine, which exhibited benzenoid rather than diradical characteristics.^18^ However, they feature steric protection groups that are stable in the monomeric state. This poses a significant obstacle to the investigation of reactivity and intermolecular interactions. Therefore, a novel molecular design was developed to circumvent the introduction of sterically protected groups.

Tovar *et al.* reported that benzo[*b*]thiophene-fused borepins exhibited enhanced aromaticity compared to the thiophene-fused variant.^19^ The aromaticity of the borepin ring is localized and enhanced according to Clar's aromatic sextet rule. This electronic stabilization strategy was extended to include phosphinines. Yamada *et al.* detailed a benzo[*b*]thieno-fused phosphinine that exhibited stability under ambient conditions owing to the preservation of aromaticity within the phosphinine ring and the distinct isolated aromaticity.^20^ Importantly, the absence of steric protection enables their application in field-effect transistor (FET) devices. We envisioned that heavier heterobenzenes could also be stabilized based on the electronic stabilization strategy and that monomeric arsinines with high planarity could be synthesized without any steric protection.

In this study, we synthesized a series of highly planar thiophene- and benzo[*b*]thiophene-fused arsinines (Fig. 1d) that were stable in their monomeric states in solution and solid states, even without steric protection groups. The structures, aromaticity, electronic properties, and reactivities of the π-expanded arsinines were investigated experimentally and computationally.

## Results and discussion

**Scheme 1:**
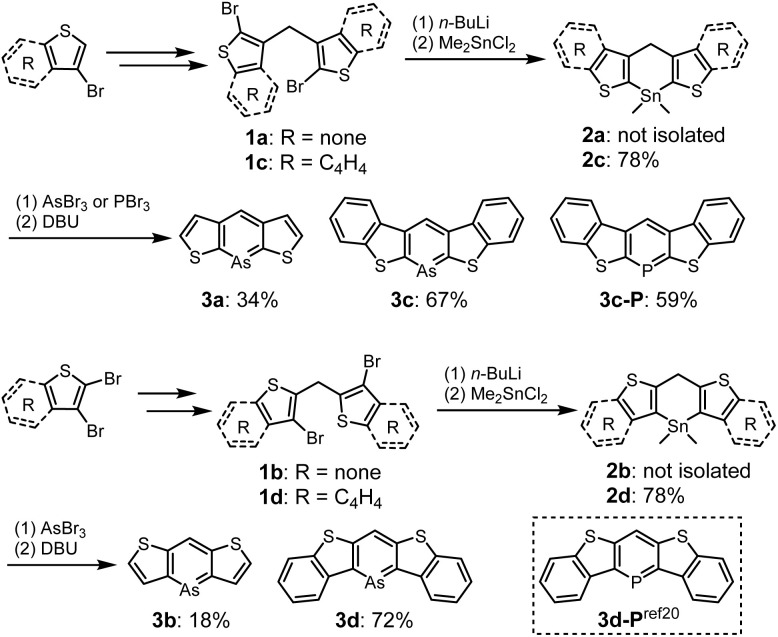
Synthesis of 3a–d and 3c-P.

The syntheses of thiophene- and benzo[*b*]thiophene-fused arsinines are outlined in Scheme 1. Bis-bromo(benzo[*b*])thienyl methanes 1a–d were synthesized in 3–4 steps, starting from commercially available thiophenes, and were isolated in moderate yields without the need for column chromatography. Bromine–lithium exchange reactions with 1a–d were conducted by treatment with *n*-butyllithium (*n*-BuLi), followed by a reaction with dimethyltin dichloride (Me_2_SnCl_2_) to yield the corresponding tin precursors 2a–d. Benzo[*b*]thieno-fused tin compounds 2c and 2d, which are stable under ambient conditions (air and light), were purified by trituration with ethanol and subjected to silica gel column chromatography. In contrast, thieno-fused tin compounds 2a and 2b were slightly sensitive and underwent hydrolysis during passing through neutral silica/alumina gel column chromatography (Fig. S10†). The crude products of 2a and 2b with sufficient purity were used in subsequent steps after removing the inorganic salts. Following the tin–arsenic exchange of 3a–d with arsenic tribromide (AsBr_3_) in benzene or tetrahydrofuran (THF) at 50 °C, three equivalents of 1,8-diazabicyclo[5.4.0]undec-7-ene were reacted. After solvent evaporation, 3c and 3d were triturated with ethanol in air, whereas 3a and 3b were isolated under argon atmosphere. Benzo[*b*]thieno-fused phosphinine 3c-P was also synthesized by replacing AsBr_3_ with phosphorus tribromide (PBr_3_), and 3d-P, a phosphorus analog of 3d, was prepared according to the literature.^20^ All the arsinines and phosphinines were characterized using NMR, HR-MS, and single-crystal X-ray analysis. In the ^1^H-NMR spectra, signals attributed to the benzylic protons of 2 at approximately 5.4 ppm disappeared, and a singlet peak that shifted to 8 ppm appeared, assigned to the proton at the 4-position of the arsinines (Fig. S11†). In the ^13^C-NMR spectra, the benzylic carbon signals shifted to the aromatic region (Fig. S12†). The ^1^H- and ^13^C-NMR spectra confirmed that all the arsinines and phosphinines exhibited high aromaticity, indicating their monomeric structure in the solution state, with no dimeric compounds detected. Although 3c and 3d were stable in solid form, they were slightly unstable to oxygen in solution and insoluble solids precipitated after storage in air for several days (*vide infra*). All arsinines 3a–d have photostability and can be stored under light in solution and solid state. In addition, thermal stabilities of air-stable arsinines 3c and 3d were evaluated by thermal gravimetric analysis (TGA) under nitrogen atmosphere (Fig. S15†). They have high thermal stabilities considering that their decomposition temperatures at 5 wt% loss (*T*_d5_) were 285 °C (3c) and 272 °C (3d).

**Fig. 2:**
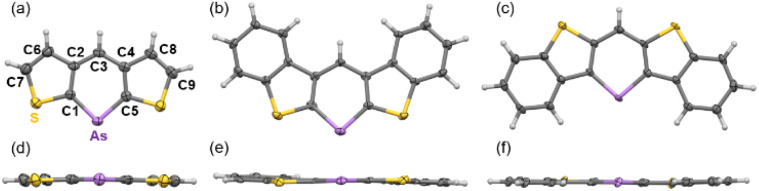
ORTEP of (a and d) 3a, (b and e) 3c, and (c and f) 3d. Thermal ellipsoids are drawn at the 50% probability level.

Single-crystal X-ray diffraction was used to obtain structural information. Single crystals of 3a–d were obtained by slowly diffusing ethanol into dichloromethane solutions of 3a, 3b, and 3d, or by slowly cooling chlorobenzene solutions of 3c and 3c-P. Two polymorphs of 3d were obtained and characterized as plate (major) and needle (minor) crystals. The plate polymorph exhibited sandwich herringbone packing, whereas the needle polymorph displayed the same 1D-columnar packing as the previously reported 3d-P. The crystal data for 3b and 3d (plate) indicate disordered structures with flipped molecules, suggesting that a detailed discussion of the structural parameters might be inappropriate. Photophysical and carrier mobility data were collected for 3d (plate) in the present study (*vide infra*). All arsinines demonstrated monomeric structures in the solution and solid states, as determined from the NMR spectra and X-ray data, respectively. The structures of 3a, 3c, and 3d (needle) are shown in Fig. 2 (for other structures, see ESI†), and the structural parameters of 3a, 3c, 3d (needle), 3c-P, 3d-P, and the carbon analog of 3d (4) are summarized in Tables 1 and 2. The arsenic atoms in these structures were dicoordinated with C1–As–C5 angles of 94.7(2)°, 94.8(2)°, and 97.4(2)°, respectively. The arsinine rings are highly planar, and the sum of the internal angles in the arsinine rings is 719.9° (3a), 720.0° (3c), and 720.0° (3d). Regarding the overall planarity of the molecules, the root mean square deviations (RMSD) from the mean plane of the entire molecule were 0.025 Å (3a), 0.051 Å (3c), and 0.029 Å (3d). The dihedral angles between the two terminal thiophene (3a) and benzene (3c and 3d) rings were 2.77°, 4.29°, and 3.01°, respectively, indicating high planarity but slight twisting with increased π-extension. Focusing on elements such as carbon and phosphorus, it was observed that the C–E–C angles decreased in the order of C (4, 119.3(4)°), P (3d-P, 100.5(1)°), and As (3d, 97.4(2)°), and the dihedral angles and RMSD values decreased in the order of C (4, 0.067 Å), As (3d, 0.029 Å), and P (3d-P, 0.026 Å). Furthermore, 3c (RMSD = 0.051 Å) exhibited larger values compared to 3d, a trend similarly observed in their phosphorus analogs (3c-P > 3d-P). In 3d-P, the strain induced by thiophene ring contraction was mitigated by the longer C–P bond, enhancing the planarity. However, in the case of arsenic, an increased C–As bond length led to reduced planarity. Additionally, in terms of fused-ring patterns, the planarity in 3c/3c-P was further decreased compared to 3d/3d-P because of steric repulsion between the protons on the benzene ring and the proton at C3.

**Table 1:** Structural data from SC-XRD analysis of 3a, 3c, 3d, 3c-P, and 3d-P

	C1–E–C5 angle/°	Sum of inner angle^a^/°	Dihedral angle^b^/°	RMSD^c^/Å
3a	94.7(2)	719.9	2.77	0.025
3c	94.8(2)	720.0	4.29	0.051
3d	97.4(2)	720.0	3.01	0.029
3c-P	97.63(9)	720.0	3.38	0.042
3d-P^20^	100.5(1)	720.0	2.62	0.026
4^20^	119.3(4)	720.0	7.42	0.067

a Sum of the inner angles of the arsinine/phosphinine ring.

b Dihedral angles between the two thiophenes in 3a and benzenes in 3c, 3d, 3c-P, 3d-P and 4.

c Root mean square deviation from the mean plane.

**Table 2:** Bond length (SC-XRD analysis) and WBIs (optimized structure) of 3a, 3c, 3d, 3c-P, 3d-P and 4. The label for numbering atoms is shown in Fig. 2

	C–E bond/Å (WBI)	C(1/5)–S bond/Å (WBI)	C(7/9)–S bond/Å (WBI)
3a	1.860(3)	1.738(3)	1.727(4)
	1.859(3)	1.728(3)	1.736(4)
	(1.22)	(1.17)	(1.16)
3c	1.853(5)	1.743(5)	1.762(4)
	1.864(5)	1.756(4)	1.757(5)
	(1.22)	(1.15)	(1.11)
3d	1.857(5)	1.763(4)	1.742(5)
	1.859(5)	1.769(5)	1.752(5)
	(1.21)	(1.12)	(1.09)
3c-P	1.739(2)	1.752(2)	1.744(2)
	1.740(2)	1.755(2)	1.750(2)
	(1.24)	(1.16)	(1.10)
3d-P^20^	1.737(2)	1.755(3)	1.751(3)
	1.732(3)	1.760(2)	1.741(3)
	(1.26)	(1.10)	(1.10)
4^20^	1.393(6)	1.753(4)	1.755(4)
	1.394(6)	1.753(4)	1.755(4)
	(1.40)	(1.11)	(1.10)

The bond lengths within the ring provided important insights into aromaticity, and we focused on the C–C, As–C, and S–C bonds. The As–C bond lengths ranged from 1.853 to 1.864 Å, positioned between the typical ranges for single (1.946–1.964 Å in triphenylarsine)^21^ and double bonds (1.816–1.827 Å in non-conjugated acyclic arsaalkenes).^22^ These lengths are comparable to those reported for arsinines, such as 1-arsanaphthalene (1.832 to 1.893 Å)^17^ and 1,4-diarsinine (1.856–1.877 Å).^18^ No differences in the As–C or P–C bond lengths were observed between the two thiophene fusion modes. The C–C bond lengths within the arsinine rings varied from 1.388 to 1.430 Å, with no significant differences between compounds 3a and 3c. The C–S bond lengths between arsinine and thiophene increased from 3a to 3c, and the two modes of thiophene fusion (3c/3d) caused little variation in these lengths. The Wiberg bond indices (WBIs) based on the optimized structures (B3LYP/def2TZVP) confirmed these tendencies. These findings suggest that the aromaticity of the arsinine rings remained consistent even after the π-extension, whereas the aromaticity of the thiophene decreased.

**Fig. 3:**
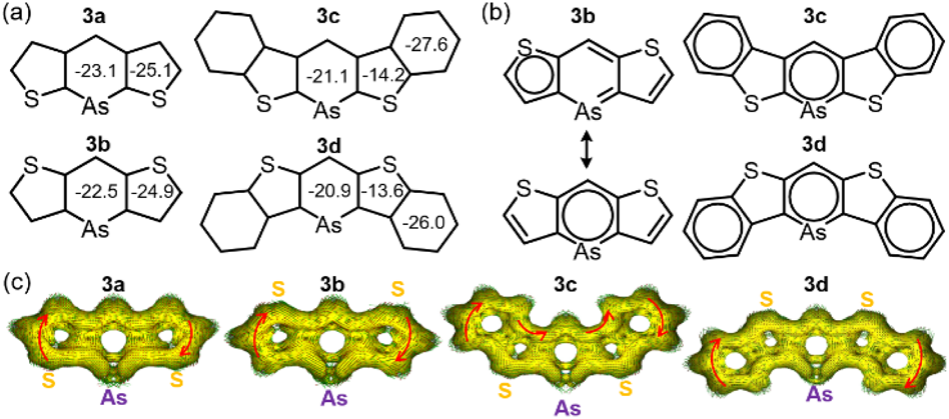
Aromaticity evaluations: (a) NICS(1)_*zz*_ values of 3a–d. (b) Clar's sextet of major resonance structures of 3a, 3c, and 3d. (c) ACID plots of 3a–d.

For further understanding of aromaticity, nucleus-independent chemical shift (NICS) analysis^23^ (GIAO-B3LYP/def2TZVP) was conducted to assess the aromaticity of compounds 3a–d (Fig. 3a). The benzene value (−29.8 ppm) showed that all arsinine rings exhibited sufficient aromaticity, ranging from −20.9 to −23.1 ppm. The NICS 1D plots of 3a–d displayed typical aromatic characteristics similar to those of the benzene rings in 3c and 3d, which also showed high aromaticity (Fig. S18a–S21a†). However, the values for thiophene differed by the π-extension. 3a and 3b exhibited −25.1 and −24.9 ppm, respectively, indicating high aromaticity, whereas 3c and 3d showed significantly lower values (−14.2 ppm and −13.6 ppm, respectively), indicating weakened aromaticity in the fused thiophene. This was consistent with the results of the X-ray crystal analysis. The NICS-XY scan^24^ (Fig. S18b–S21b†) revealed that, although 3a and 3b had three local circuits at the thiophenes and arsinine, 3c and 3d had three aromatic circuits at the benzenes and arsinines. Similar observations have been reported for benzo[*b*]thiophene-fused borepins,^19^ in which the arsinine rings of 3c and 3d exhibit isolated aromaticity. The phosphinine ring in 3c-P showed higher aromaticity (−23.1 ppm, Fig. S22–S23†) than that of the arsinines, although the NICS(1)_*zz*_ values for the thiophene and benzene rings of 3c-P (−13.6 and −27.6 ppm, respectively) were similar to those of 3c. The major resonance structures of 3a and 3b were localized on thiophene and arsinine, whereas those of 3c and 3d were localized only on arsinine and the two benzene rings (Fig. 3b). To further investigate the aromaticity of 3a–d, an anisotropically induced current density (ACID) analysis (CSGT-B3LYP/def2TZVP) was performed (Fig. 3c).^25^ The ACID plots revealed that arsinines 3a–d and phosphinine 3c-P (Fig. S24†) demonstrated clockwise currents at the periphery of the molecule, indicating global aromaticity and a 14 (3a and 3b) or 22π (3c and 3d) electron aromatic system. Additionally, focusing on the As–C moieties, it was observed that π-electron delocalization was diminished due to weak overlap between the 4p orbital of arsenic and the 2p orbital of carbon. Notably, this represents the first example of a stable 14 or 22π-electron arsinine synthesis.

**Fig. 4:**
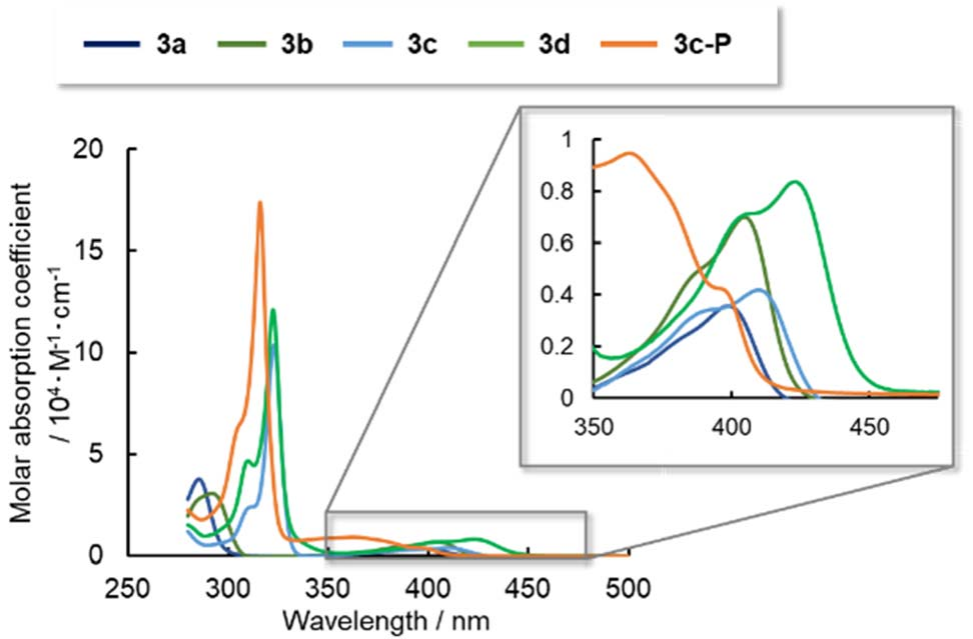
UV-vis absorption spectra of 3a–d and 3c-P in 2-MeTHF solutions (1.0 × 10^−5^ M).

To determine the electronic structures of arsinines 3a–d and phosphinine 3c-P, their absorption/emission spectra were measured in 2-methyltetrahydrofuran (2-MeTHF) solution and are summarized in Fig. 4 and Table 3. Arsinines and phosphinines exhibited two absorption bands in the 280–320 nm and 350–400 nm ranges, respectively. The substitution patterns had a minimal effect on the optical properties of the monomeric states, with a slight red-shift from 3a to 3c or 3b to 3d. The longest absorption maxima (*λ*_abs_) of 3a and 3c were red-shifted due to the expanded conjugation. Furthermore, in examining the effect of π-extension by comparing 3c and 3d the absorption wavelengths were also red-shifted.

**Table 3:** Photophysical properties of 3a–d and 3c-P in solutions^a^

	*λ*_abs_ b [nm]	*ε* c [M^−1^ cm^−1^]	298 K				77 K		
			*λ*_ex_ d [nm]	*λ*_em_ e [nm]	*Φ* f	*τ* g	*λ*_em_ e [nm]	*Φ* f	*τ* g
3a	400	3500	286	424	0.02	53.4 ns	420	0.89	12.7 ns
3b	404	6200	—	n.d.	n.d.	n.d.	n.d.	n.d.	n.d.
3c	410	4200	322	440	<0.01	1.0 ns	430, 668	0.01, 0.08	0.71 ns, 1.1 ms
3d	422	8400	322	455	0.02	1.3 ns	440, 570	0.04	2.1 ns, 35 ms
3c-P	398	4000	316	420	0.01	34.3 ns	390, 575	0.06, 0.12	36.8 ns, 127 ms

a Measured in 2-MeTHF (1.0 × 10^−4^ M for 3a–b, 1.0 × 10^−5^ M for 3c–d and 3c-P) solutions.

b The longest absorption maxima.

c Molar excitation coefficients at the absorption maxima.

d Excitation maxima monitored at the emission maxima.

e Emission maxima monitored at the excitation maxima.

f Absolute quantum yield.

g Emission lifetime.

**Fig. 5:**
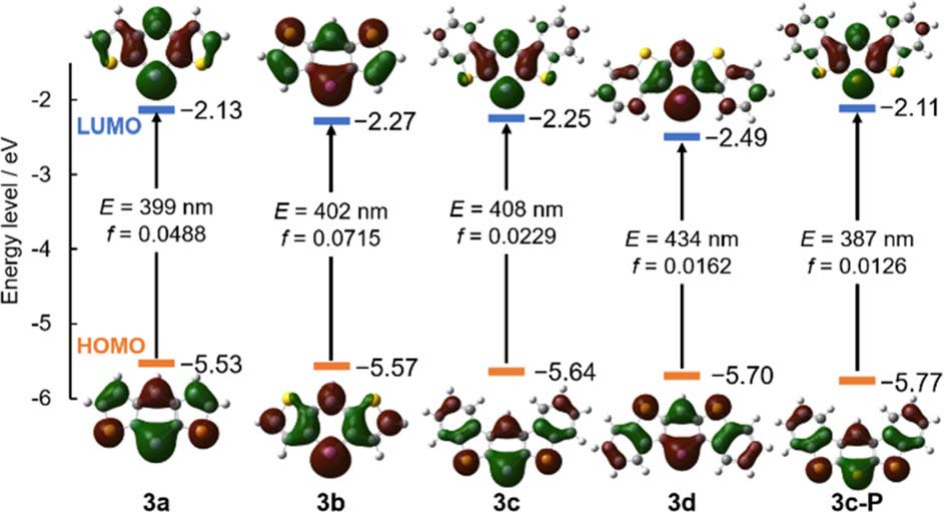
Frontier orbitals of 3a–d and 3c-P, their energy levels, transition energy (*E*), and oscillator strength (*f*) for the HOMO–LUMO transitions. Isovalue = 0.025.

To elucidate the origin of the absorption, time-dependent DFT (TD-DFT) calculations were conducted at the B3LYP/def2TZVP level of theory, and the orbital distributions and energy levels are summarized in Fig. 5. The electronic transitions of all compounds were attributed to the HOMO–LUMO transitions, with the oscillator strengths of 3a–d estimated to be small (*f* = 0.0162–0.0715). Regarding the orbital levels, the HOMO and LUMO were stabilized after the π-extension from 3a to 3c. The delocalization of the LUMO stabilized the LUMO, whereas the stabilization of the HOMO was attributed to the stabilizing effect of the Clar's sextets in the outer benzene rings, thus lowering the HOMO level owing to the aromatic stabilization energy.^26^ In view of the differences in substitution patterns between 3c and 3d, the changes had minimal effect on the HOMO, whereas the LUMO was stabilized by 0.24 eV due to the increased delocalization. When substituting arsenic for phosphorus, the orbital distributions remained almost the same; however, the bandgap was narrowed due to the smaller energy splitting of π–π* because of the poor overlaps between carbon and the heavier atom. Furthermore, the spatial distribution of the orbitals of 3c compared to those of 3c-P may enhance the intermolecular interactions through the arsenic atoms.^27^

**Fig. 6:**
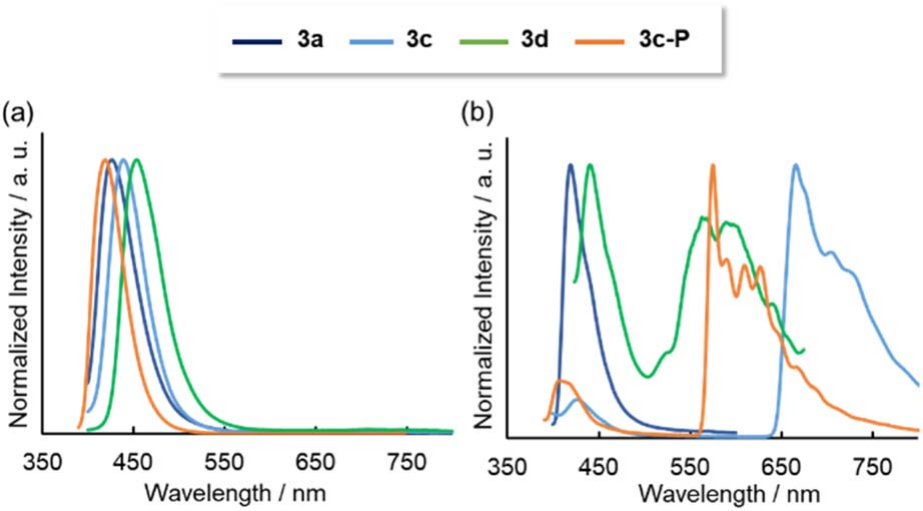
Emission spectra of 3a–d and 3c-P in 2-MeTHF solutions (1.0 × 10^−5^ M) at (a) 298 K and (b) 77 K.

Photoluminescence (PL) spectra were obtained for solution and solid states. In solution, all the compounds except 3b exhibited fluorescence spectra, with the order of the fluorescence wavelengths being consistent with the order of their absorption wavelengths (Fig. 6a). For 3b, the small energy gap between S_1_ and T_2_ (0.003 eV) might promoted the intersystem crossing (Fig. S26†). The triplet excitons were deactivated *via* non-radiative pathway probably because the large energy gap between T_2_ and T_1_ (1.51 eV) inhibited the internal conversion. The emission lifetimes (*τ*) were ns-order at 298 K, implying that the emissions were fluorescence. No excimer emissions were observed even at higher concentrations (1.0 × 10^−3^ M for 3a, 1.0 × 10^−4^ M for 3c, 3d, 3c-P). The quantum yields were <0.02, as the oscillator strengths of the S_0_ → S_1_ transitions were small due to the partial change in orbital symmetry. Low fluorescence efficiencies were observed in λ^3^-phosphinines due to the low differences of symmetry between the HOMO and LUMO.^4^ Additionally, PL spectra were measured in a glass matrix of 2-MeTHF at 77 K (Fig. 6b). Although 3a showed only fluorescence even at 77 K, the benzothiophene-fused compounds 3c, 3d, and 3c-P displayed fluorescence and phosphorescence, judging from the co-existence of the ns- and ms-order *τ* values. DFT analysis revealed that the T_2_ levels of 3c, 3d, and 3c-P were less than their S_1_ levels, and their energy gaps between the T_2_ and S_1_ were sufficiently small (3c: 0.34 eV, 3d: 0.23 eV, 3c-P: 0.37 eV) to promote intersystem crossing, resulting in phosphorescence. In contrast, the T_2_ level of 3a was higher than the T_1_ level, and the energy gap between T_1_ and S_1_ was excessively large (1.45 eV), inhibiting phosphorescence. The low-lying T_2_ of 3c, 3d, and 3c-P was probably due to delocalization along the relatively large conjugated system.

**Fig. 7:**
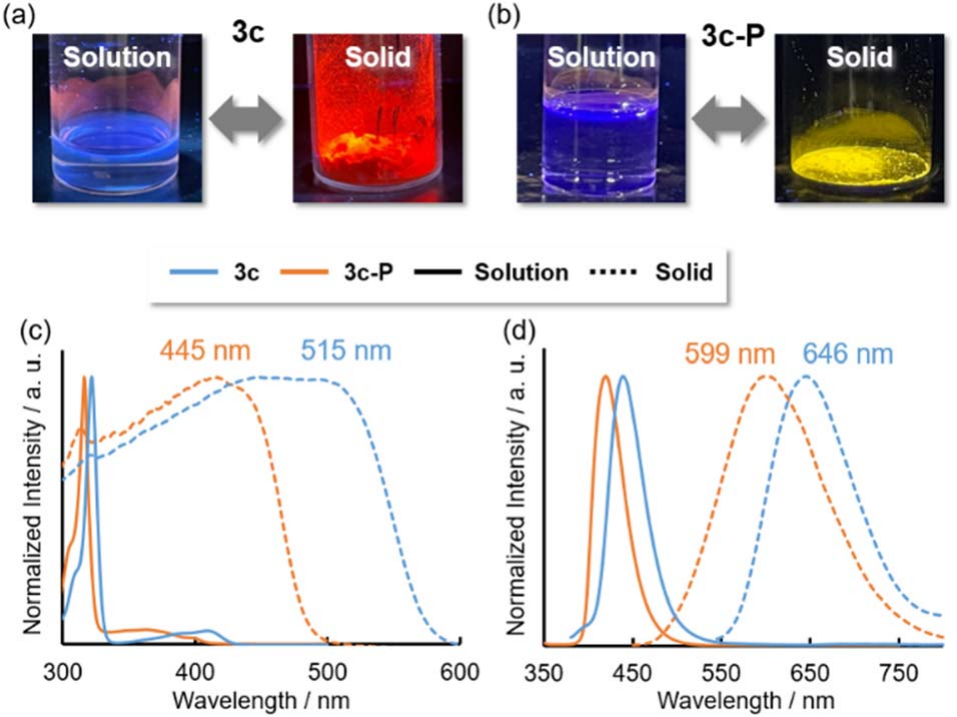
Pictures of luminescence in solution and solid state of (a) 3c and (b) 3c-P. (c) Absorption and (d) emission spectra of 3c and 3c-P in solution (solid lines) and solid state (dashed lines).

In the solid state, although 3a, 3b, 3d, and 3d-P showed negligible emission, 3c and 3c-P exhibited intense red and yellow emissions, respectively (Fig. 7a and b), in contrast to their behavior in solution. The absorption and emission spectra are shown in Fig. 7c and d, respectively. Significant redshifts were observed for both compounds, accompanied by a broadening of the spectra. The ns-order *τ* values of 3c and 3c-P (0.53 and 1.6 ns, respectively) indicated that the long-wavelength emissions in the solid states were attributed to fluorescence.

**Fig. 8:**
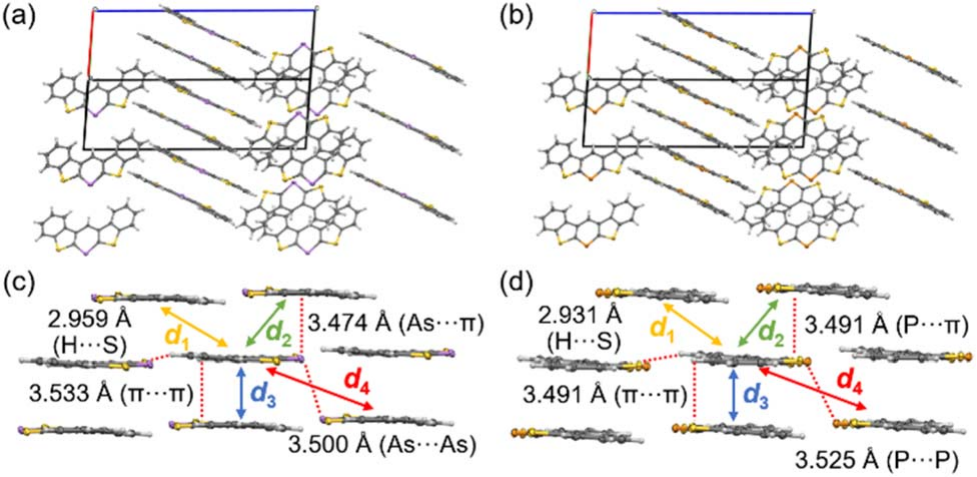
Packing structures of (a) 3c and (b) 3c-P. Intermolecular interactions of (c) 3c and (d) 3c-P with showing dimers *d*_1_–*d*_4_.

To understand the solid-state emission behavior of 3c and 3c-P, their packing structures were investigated; for non-emissive solid samples (3a, 3b, and 3d), please see ESI (Fig. S1–S4†). 3c and 3c-P exhibited similar columnar packing structures (Fig. 8a and b) with numerous intermolecular interactions through the π plane (for dimers *d*_1_–*d*_3_) and H⋯S interactions (Fig. 8c and d). Notably, As⋯As (3c) and P⋯P (3c-P) interactions (for dimer *d*_4_) were observed at distances of 3.500 Å and 3.525 Å, respectively. Although the atomic radius of arsenic (1.85 Å) is larger than that of phosphorus (1.80 Å),^28^ the As⋯As distance was shorter than the P⋯P distance. This indicates stronger interactions of *d*_4_ in 3c due to the more spatially expanded molecular orbitals involving the 4p orbital of arsenic, which promotes efficient As⋯As interaction. The symmetry-adapted perturbation theory (SAPT) was employed to reveal the interaction energies and components, as summarized in Table 4.^29^ For the C⋯C stacked dimer (*d*_1_ and *d*_3_), there were few differences in composed energies between 3c and 3c-P. Conversely, in the π-stacked dimer interacting with As or P (*d*_2_), 3c exhibited closer interaction (3.474 Å) than 3c-P (3.491 Å), with electrostatic (*E*_elst_) and dispersion (*E*_disp_) energies (−10.5 and −25.8 kcal mol^−1^, respectively), more favorable than those of 3c-P (−8.05 and −23.7 kcal mol^−1^, respectively). This indicates that the more spatially enlarged arsenic orbitals contribute significantly to intermolecular interactions. For the Pn⋯Pn interaction (*d*_4_), *E*_disp_ was the dominant component, with *E*_elst_ also contributing. The *d*_4_ in 3c displayed higher *E*_disp_ and *E*_elst_ values than those in 3c-P due to its more spatially expanded orbitals and natural population analysis charge, as confirmed by DFT calculations (Table S18†). However, the short As⋯As the distance of 3c resulted in greater destabilization due to exchange energy (*E*_exch_ = 13.2 kcal mol^−1^), making the *E*_total_ (−4.76 kcal mol^−1^) lower than that of 3c-P (−4.94 kcal mol^−1^).

**Table 4:** SAPT analysis (SAPT0/jun-cc-PVDZ) of the selected dimer of 3c and 3c-P in the solid state

		*E*_elst_ a	*E*_exch_ b	*E*_ind_ c	*E*_disp_ d	*E*_total_ e
3c	*d*_1_	−5.89	8.72	−1.19	−12.3	−10.6
	*d*_2_	−10.5	21.6	−2.11	−25.8	−16.8
	*d*_3_	−11.0	25.1	−2.58	−35.5	−24.0
	*d*_4_	−5.78	13.2	−2.39	−9.83	−4.76
3c-P	*d*_1_	−6.40	9.97	−1.33	−12.9	−10.7
	*d*_2_	−8.05	17.5	−1.69	−23.7	−15.9
	*d*_3_	−12.0	27.1	−2.73	−36.5	−24.1
	*d*_4_	−3.50	9.18	−1.55	−9.08	−4.94

a Electrostatic.

b Exchange.

c Induction.

d Dispersion.

e Total energies (kcal mol^−1^).

TD-DFT calculations with a dispersion-corrected function (M06/def2-TZVP) were conducted to investigate the reasons for the bathochromic shift and the aggregation-induced emission enhancement (AIEE) properties in selected dimers with π–π (*d*_1_–*d*_3_) or As⋯As (*d*_4_) interactions (Table S19†). At this calculation level, the transition energy of 3c was estimated to be 400.7 nm with a small oscillator strength (*f* = 0.0266). Dimers *d*_1_–*d*_3_ with π–π stacking showed slightly bathochromic shifts from the monomeric state (401.9–423.6 nm); however, their oscillator strengths were still small (0.0126–0.0772). In contrast, dimer *d*_4_ exhibited a bathochromic shift (475.6 nm) and a large oscillator strength (0.2039). For dimer *d*_4_, the HOMO and LUMO levels increased and decreased, respectively. Furthermore, the transition dipole moment of *d*_4_ increased compared to that of the monomeric state because of the symmetry-allowed transitions of the HOMO and LUMO, thereby facilitating the S_0_ and S_1_ transitions of 3c and 3c-P. Consequently, 3c and 3c-P exhibited enhanced fluorescence. The Pn⋯Pn interaction for *d*_4_ resulted in AIEE properties, as these compounds showed more efficient fluorescence (*Φ* = 0.10 and 0.18, respectively) in the solid state than in the solution state (*Φ* = 0.01).

We then evaluated the hole mobilities of 3c, 3d, and 3c-P by conducting flash photolysis time-resolved microwave conductivity (TRMC) measurements (Fig. S16 and S17†).^30^ Photocarriers were generated by excitation at 355 nm. The TRMC profiles are shown in Fig. S16,† where *φ*Σ*μ* is the product of *φ* (the quantum yield of photogenerated charge carriers) and Σ*μ* (the sum of charge carrier mobilities). The order of *φ*Σ*μ* maxima was as follows: 3d (4.3 × 10^−5^ cm^2^ V^−1^ s^−1^), 3c-P (3.7 × 10^−5^ cm^2^ V^−1^ s^−1^), 3c (2.4 × 10^−5^ cm^2^ V^−1^ s^−1^), and 3d-P (1.7 × 10^−5^ cm^2^ V^−1^ s^−1^). Moreover, the effective lifetime of the TRMC decay was longest for 3d (4.0 μs) followed by 3c-P (2.0 μs) and 3c (1.6 μs), exhibiting the superior photoconductive character of 3d; the lifetime of 3d-P was not determined because of the weak signal. Transfer integrals and reorganization energies were calculated for 3c and 3c-P, whereas calculations for 3d were not conducted because of their disordered structures (Table S9†). Although the *φ*Σ*μ* values were lower than those of typical thienoacenes,^31^ the transfer integrals through *d*_4_ dimer showed considerably higher values (176.0 meV) despite their small interaction surface (Table S21†). This is due to their large orbital coefficients and extensive orbitals. Thus, these theoretical evaluations provide insights into novel molecular designs involving acenes embedded with heavy pnictogens for OFET applications with potential charge transport abilities.^32^

**Scheme 2:**
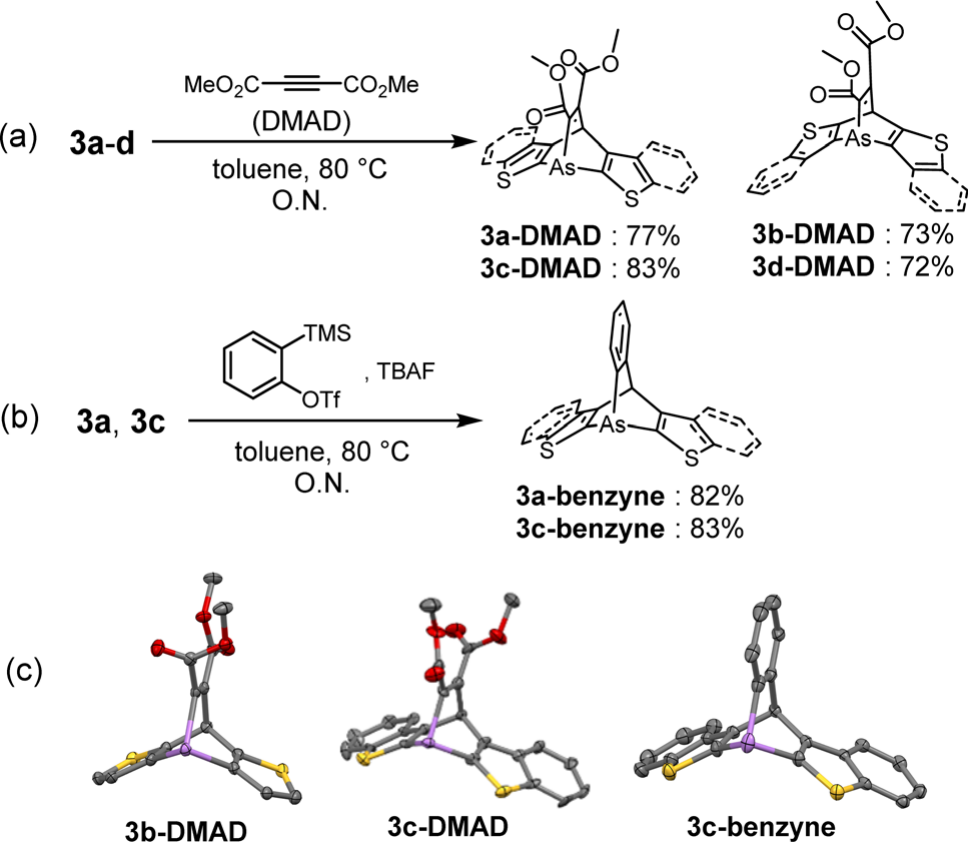
[4 + 2] cycloaddition of arsinines with (a) dimethylacetylene dicarboxylate (DMAD) (for 3a–d) and (b) *o*-benzyne (for 3a and 3c). (c) ORTEPs of 3b-DMAD, 3c-DMAD, and 3c-benzyne. Thermal ellipsoids are drawn at the 50% probability level. Hydrogen atoms were omitted for clarity.

The reactivities of the arsinines were also investigated. Heterobenzenes are known to undergo [4 + 2] or [2 + 2] cycloadditions with themselves and with dienes and alkynes.^33^ Compounds 3a–d were reacted with dimethyl acetylenedicarboxylate (DMAD), a dienophile, to yield the [4 + 2] cycloadducts, arsabarrelenes 3-DMAD (Scheme 2a). In addition, compounds 3a and 3c reacted with *o*-benzyne to yield 3a-benzyne and 3c-benzyne, respectively (Scheme 2b). The structures of these cycloadducts were characterized using NMR spectroscopy and, for some compounds, using single-crystal X-ray diffraction (Scheme 2c). Furthermore, the thermal activities of the compounds were investigated. Degassed chlorobenzene solutions of 3a–d were heated to reflux under N_2_ atmosphere, and no dimerized products were detected by ^1^H-NMR spectroscopy.

The complexation behavior was investigated. Arsines have a lone pair with high s-character and less directional coordination behavior.^34^ It was assumed that unique molecular arrangements could be achieved by the flexible coordination of arsines and intermolecular interactions through large arsenic orbitals. First, we attempted to synthesize gold(i) complexes by reacting 3d with gold(i) chloride (AuCl). However, insoluble materials and gold nanoparticles were generated. This probably occurred because Au(i) oxidizes 3d, reducing it to Au(0) (*vide infra*). Therefore, tungsten hexacarbonyl (W(CO)_6_) was selected as a redox-neutral metal source. The reaction with 3d failed due to the low solubility of the complex, resulting in an insoluble material. Thus, 3a reacted with W(CO)_6_ because of its better solubility than 3d.

**Fig. 9:**
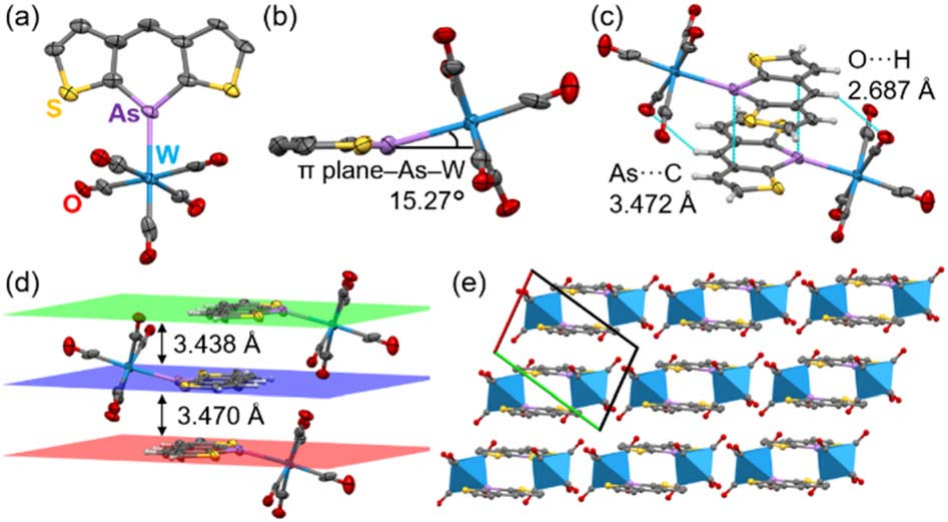
Crystal structure of 3a-W(CO)_5_. (a) Front and (b) side view of 3a-W(CO)_5_. (c) Face-to-face stacking dimer, (d) mean planes of each column, and (e) 1D-columnar packing separated by W(CO)_5_ moiety (blue hexagon).

After recrystallization from THF/hexane, yellow crystals suitable for X-ray structural analysis were obtained. Owing to poor data, the precise parameters of the C–C bonds are not discussed. Surprisingly, the W(CO)_5_ moiety was tilted 15.27° from the arsinine plane (Fig. 9a and b). To the best of our knowledge, this coordination geometry is the first example of μ^1^-coordination in phosphinines and arsinines. NBO analysis revealed that the hybridization of the lone pair comprised 72% 4s orbitals and 28% 4p orbitals for 3a. Considering the values for triphenylarsine (60% 4s and 40% 4p), pyridine (29% 2s and 71% 2p), and phosphinine (61% 3s and 39% 3p, Table S18†), the lone pair of 3a had fewer directional orbitals, thus enabling flexible coordination. To elucidate the differences in the coordination behaviors of the pnictogens (N, P, and As), the energy profiles dependent on the coordination angle were calculated (Fig. S27†). The most stable geometries for all elements were planar, with nitrogen and phosphorus showing energies 1.64 and 1.26 kcal mol^−1^ higher, respectively, at 18° than at 0°. In contrast, arsenic showed an energy increase of 0.44 kcal mol^−1^ at 18°, indicating that attractive interactions, such as hydrogen bonds and π–π interactions, can induce the flexible coordination geometry of 3a-W(CO)_5_. The optimized structure of 3a-W(CO)_5_ (B3LYP-GD3BJ/def2-TZVP) showed planar coordination geometry, suggesting that the distorted coordination direction was caused by intermolecular interactions in the crystal packing. 3a-W(CO)_5_ exhibited face-to-face stacking with distances of 3.470 Å and 3.438 Å between the mean planes (Fig. 9d). The π–π interactions included As⋯C interactions at 3.472 Å due to the large arsenic orbitals. Additionally, hydrogen bonds between the carbonyl oxygen and the hydrogen at the 4-position of arsinine at 2.678 Å in each column contributed to the distortion of the As–W coordination (Fig. 9c). Furthermore, 3a-W(CO)_5_ formed a 1D-columnar packing, with each column separated by the W(CO)_5_ moiety (Fig. 9e). The optimization of the 3a-W(CO)_5_ dimer (B3LYP-GD3BJ/def2TZVP) reproduced a distorted structure with a distortion angle of 14.30° (Fig. S28†). Hydrogen bonds and π–π interactions were also confirmed by non-covalent interaction analysis based on the optimized structure (Fig. S29†).^35^

**Scheme 3:**
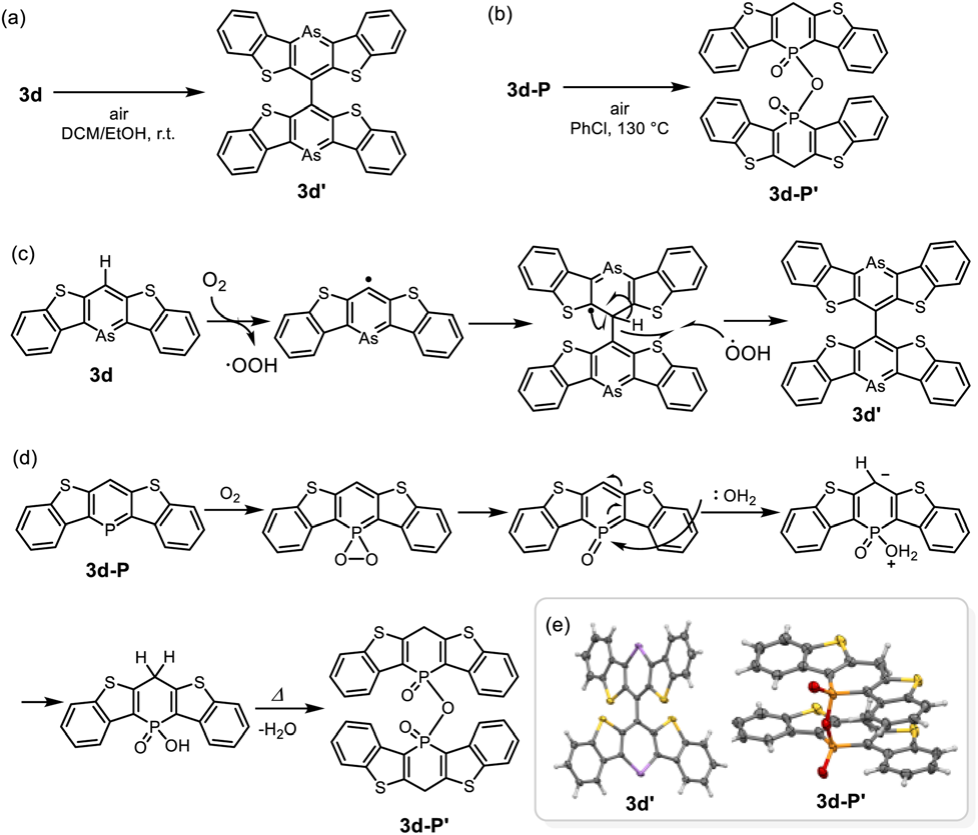
Dimerization of (a) 3d and (b) 3d-P in air, and their plausible mechanisms for (c) 3d and (d) 3d-P. (e) ORTEPs of 3d′ and 3d-P′. Thermal ellipsoids are drawn at the 50% probability level.

Finally, we investigated the reactivity towards oxygen (O_2_) for compounds 3d and 3d-P (Scheme 3). As mentioned above, 3d was slightly unstable to oxygen in solution; insoluble solids precipitated after storage in air for several days even under dark condition. When a solution of 3d was exposed to air, brown crystals were formed. X-ray structural analysis and ^1^H-NMR revealed that 3d dimerized at the 4-position *via* a single bond to form 3d′ (Scheme 3a and e). Based on the bond lengths and NICS(1)_*zz*_ value of the arsinine ring (−20.9 ppm), 3d underwent dehydrogenative-oxidative dimerization while retaining its aromaticity. In contrast, the exposure of 3d-P to air resulted in the precipitation of insoluble materials that could not be analyzed using NMR. Heating a solution of 3d-P in air yielded a single crystal of the oxidative decomposition product, which was identified as the dimerized phosphinic anhydride 3d-P′ (Scheme 3b and e). Owing to the poor quality of the crystal, the detailed structural parameters are not discussed. The bond lengths in the P-containing six-membered rings exhibit bond alternation. The two P–O bonds had distinct characteristics; the dangling P–O bond was shorter than the bridging P–O bond, indicating that 3d-P′ is a phosphinic acid. Based on the NICS values of 3d and 3d-P in the optimized structures, the aromaticity of arsinine was lower than that of phosphinine. However, arsinine 3d dimerized while retaining its aromaticity, whereas phosphinine 3d-P′ lost its aromaticity. Plausible mechanisms for the oxidative dimerization of 3d and 3d-P are shown in Scheme 3c and d, respectively. In 3d, O_2_ initially abstracted hydrogen at the 4-position, and the resulting arsinine radical dimerized. The hydroxyl radicals then abstract hydrogen again, yielding arsinine dimer 3d′. Other oxidants such as AuCl (*vide supra*), *N*-bromosuccinimide, *N*-iodosuccinimide, and iodine also induced the dimerization of 3d to 3d′ (Fig. S13 and S14†). For phosphinine 3d-P, O_2_ first attacks the phosphorus atom, generating phosphinine oxide. This intermediate, known for its high instability,^36^ reacts with water to form phosphinic acid, which is subsequently dehydrated to yield phosphinic anhydride 3d-P′. These differences in the oxidative products arise from the intrinsic properties of the elements. Arsinine has lower aromaticity and oxophilicity, allowing O_2_ to attack the most reactive hydrogen instead of the arsenic atom. In contrast, phosphinine, which has higher aromaticity and oxophilicity, experiences oxygen attack at the phosphorus atom rather than the hydrogen in the phosphinine ring.

## Conclusions

We successfully synthesized thiophene- and benzo[*b*]thiophene-fused arsinines (3a–d), marking the first instance of stable and planar π-extended arsinines. These compounds exhibited remarkable planarity and bond equalization, including the As–C bonds. NICS and ACID analyses confirmed their 14- or 22π global aromaticity, although the degree of local aromaticity varied depending on the fused rings. The absorption and PL spectra of these arsinines were bathochromically shifted relative to those of their phosphinine analogs (3c-P and 3d-P), which was attributed to the incorporation of heavier pnictogen atoms in the heterocycles. Notably, compound 3c demonstrated emission enhancement with a significant bathochromic shift in the solid state, compared to its solution-phase emission, due to dimer formation through As⋯As interactions. The tungsten(0) complexes 3a-W(CO)_5_ exhibited highly distorted As⋯W coordination in the solid state, influenced by the less directional lone pair of the As atom and 1D columnar molecular packing. Additionally, [4 + 2] cycloadditions of 3c and 3d with alkynes and benzynes were observed. Under an O_2_ atmosphere, 3d dimerized at the 4-position while retaining its aromaticity, whereas the P atom in 3d-P underwent oxidation, losing its aromaticity, which was driven by their differing affinities for oxygen. This study provides critical insights into the structural, electronic, coordination, and reactivity properties of heavier element-containing heterobenzenes obtained from stable and planar arsinines. Ongoing efforts are directed towards further diversifying and exploring applications of π-extended arsinines.

## Data availability

The authors confirm that the data supporting the findings of this study are available within the article and its ESI.†

## Author contributions

A. Sumida: synthesis, structural analysis, data curation, writing – original draft; A. Saeki: TRMC measurements, data curation, writing – review and editing; K. Matsuo: evaluation of OFET, computational calculations, data curation, writing – review and editing; K. Naka: conceptualization, investigation, writing – review and editing, supervision; H. Imoto: conceptualization, investigation, writing – original draft, writing – review and editing, funding acquisition project administration, supervision.

## Conflicts of interest

There are no conflicts to declare.

## Supplementary Material

SC-OLF-D4SC06590E-s001

SC-OLF-D4SC06590E-s002
