# Supplementary material for: Dithienoarsinines: stable and planar π-extended arsabenzenes
Source: Chem Sci. 2024 Nov 19;16(3):1126–35. doi: 10.1039/d4sc06590e (PMC11632611; doi:10.1039/d4sc06590e)
Supplement: SC-016-D4SC06590E-s001 [file SC-016-D4SC06590E-s001.pdf]

Supplementary Information (SI) for Chemical Science.
This journal is © The Royal Society of Chemistry 2024

Dithienoarsinines: Stable and Planer π-Extended Arsabenzenes

Akifumi Sumida,[a] Akinori Saeki,[bc] Kyohei Matsuo,[d] Kensuke Naka,[ae] and Hiroaki Imoto[aef]*

[a] Faculty of Molecular Chemistry and Engineering, Graduate School of Science and Technology, Kyoto Institute of Technology, Goshokaido-cho, Matsugasaki, Sakyo-ku, Kyoto 606-0962, Japan

[b] Department of Applied Chemistry, Graduate School of Engineering, Osaka University, 2-1 Yamadaoka, Suita, Osaka 565-0871, Japan

[c] Innovative Catalysis Science Division, Institute for Open and Transdisciplinary Research Initiatives (ICS-OTRI), Osaka University, 1-1 Yamadaoka, Suita, Osaka 565-0871, Japan

[d] Institute for Chemical Research, Kyoto University, Gokasho, Uji, Kyoto 611-0011, Japan

[e] Materials Innovation Lab, Kyoto Institute of Technology, Goshokaido-cho, Matsugasaki, Sakyo-ku, Kyoto 606-0962, Japan.

[f] Fusion Oriented Research for Disruptive Science and Technology (FOREST), Japan Science and Technology Corporation (JST), Honcho 4-1-8, Kawaguchi, Saitama 332-0012, Japan.

Contents:

1 General Information
2 Materials
3 Synthesis
4 X-ray diffraction analysis
5 Photophysical properties
6 Reactivity
7 Time-resolved microwave conductivity
8 DFT calculations
9 NMR spectra

1. General Information

All reactions were carried under dry nitrogen atmosphere otherwise noted. Unless otherwise noted, all commercial reagents were used without additional purification. All dry solvents were stored with molecular sieves. ^{1}H (400 MHz), ^{13}C{^{1}H} (100 MHz), ^{31}P (162 MHz) NMR spectra were recorded on a Bruker AVANCE III 400 NMR spectrometer using Me_4Si (TMS) as an internal standard. The following abbreviations are used; s: singlet, d: doublet, t: triplet, q: quartet, m: multiplet. High-resolution mass spectra (HRMS) were obtained on a JEOL JMS-SX102A spectrometer. Matrix assisted laser desorption ionization time-of-flight mass spectrometry (MALDI-TOF-MS) was recorded on a Bruker Autoflex II instrument (Bruker Daltonics, Billerica, MA, USA): *trans*-2-[3-(4-tert-butylphenyl)-2-methyl-2-propenylidene]malononitrile (DCTB) matrix (20 mg/mL in $CHCl_3$) and sodium trifluoroacetate cationizing agents (1 mg/mL in THF). X-ray diffractometry (XRD) studies were performed on a Rigaku SmartLab with Cu Kα radiation (λ = 1.5406 Å) in the $2\theta/\theta$ mode at room temperature. The 2θ scan data were collected at 0.01° intervals and the scan speed was 10° (2θ)/min. UV/Vis spectra were recorded on a JASCO spectrophotometer V-670 KNN. Emission and excitation spectra were obtained on an FP-8500 (JASCO) spectrometer and the absolute PL quantum yields (Φ) were determined by using a JASCO ILFC-847S; the quantum yield of quinine sulfate as a reference was 0.52, which is in agreement with the literature value. Emission lifetimes were measured by using a Quantaurus-Tau (Hamamatsu Photonics, Shizuoka, Japan) instrument.

2. Materials

All solvents used for the synthesis, *N*-bromosuccinimide, *N*-iodosuccinimide, magnesium, and Cerite® were purchased from FUJIFILM Wako Pure Chemical Industry, Ltd. *n*-Butyllithium (*n*-BuLi, 1.51 M in hexane) was purchased from Kanto Chemical Co. Inc. Dimethyltin dichloride, ethyl formate, lithium chloride (LiCl), triethylsilane (Et_3SiH), dimethyl acetylenedicarboxylate (DMAD), 2-TMS-benzenetriflate, and tetrabutylammonium fluoride (TBAF, 1.0 M in THF) were purchased from Tokyo Chemical Industry Co. Ltd. Trifluoroacetic acid and 1,8-diazabicyclo[5.4.0]undec-7-ene (DBU) were purchased from Nacalai Tesque. Phosphorus tribromide (1.0 M in DCM), and tungsten hexacarbonyl ($W(CO)_6$) were purchased from Sigma-Aldrich. Bis(2-bromothinyl)methane,[1] bis(3-bromothinyl)methane,[2] tribromoarsine ($AsBr_3$),[3] bis-2-(3-bromobenzo[*b*]thienyl)methane,[4] and **3d-P**[5] were synthesized according to literature procedures.

3. Synthesis

8,8-Dimethyl-4,8-dihydrostano[2,3-*b*:6,5-*b*']dithiophene (**2a**)

To a solution of bis(2-bromothinyl)methane (1.09 g, 3.21 mmol) in ether (60 mL) was added *n*-BuLi (1.51 M in hexane, 4.36 mL, 6.58 mmol) dropwise at −78 °C and the mixture was stirred for 30 min at the same temperature. Then, dimethyltin dichloride (0.7756 g, 3.53 mmol) in ether (10 mL) was added dropwise into the mixture at the same temperature, and the mixture was warmed to room temperature. After stirring at room temperature overnight, the volatiles were removed *in vacuo*. The crude product was dissolved in hexane and passed through the Cerite® and the volatiles were removed to give the title compound with sufficient purity, which was used for the next reaction without purification. ^{1}H-NMR ($CDCl_3$, 400 MHz): δ 7.65 (d, J = 4.6 Hz, 2H), 7.20 (d, J = 4.6 Hz, 2H), 4.38 (s, 2H), 0.58 (s, 6). ppm. HR-FAB-MASS (m/z): calculated for $C_{11}H_{13}S_2Sn$ $[M+H]^+$; 328.9475, observed; 328.9483.

Dithieno[2,3-*b*:3',2'-*e*]arsinine (**3a**)

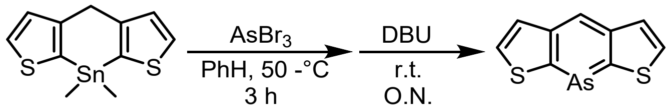

The crude mixture of **2a** was dissolved in benzene (15 mL) was added arsenic tribromide (1.11 g, 3.53 mmol) and the mixture was stirred for 3 h at 50 °C. Then, DBU (1.5 mL, 10 mmol) was added into the mixture at room temperature, and stirred overnight. The volatiles were removed *in vacuo*. The crude product was washed with ethanol in argon-filled glove box to give the title compounds as yellow solid (272.4 mg, 1.08 mmol, 34%). ^{1}H-NMR ($CDCl_3$, 400 MHz): δ 8.62 (s, 1H), 7.71 (d, J = 5.3 Hz, 2H), 7.64 (d, J = 5.3 Hz, 2H). ppm. HR-FAB-MASS (m/z): calculated for $C_9H_5S_2As$ $[M]^+$; 251.9049, observed; 251.9058.

8,8-Dimethyl-4,8-dihydrostano[1,2-*b*:4,3-*b*']dithiophene (**2b**)

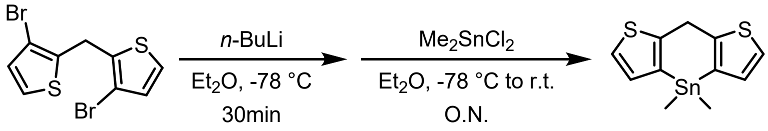

To a solution of bis(3-bromothinyl)methane (0.511 g, 1.51 mmol) in ether (30 mL) was added *n*-BuLi (1.51 M in hexane, 2.05 mL, 3.10 mmol) dropwise at −78 °C and the mixture was stirred for 30 min at the same temperature. Then, dimethyltin dichloride (0.365 g, 1.66 mmol) in ether (5 mL) was added dropwise into the mixture at the same temperature, and the mixture was warmed to room temperature. After stirring at room temperature overnight, the volatiles were removed *in vacuo*. The crude product was dissolved in benzene and passed through the Cerite® and the volatiles were removed to give the title compound with sufficient

purity, which was used for the next reaction without purification. ^{1}H-NMR ($CDCl_3$, 400 MHz): δ 7.32 (d, J = 4.9 Hz, 2H), 7.12 (d, J = 5.0 Hz, 2H), 4.61 (s, 2H), 0.48 (s, 6H). ppm. HR-FAB-MASS (m/z): calculated for $C_{11}H_{13}S_2Sn$ $[M+H]^+$; 328.9475, observed; 328.9486.

Dithieno[3,2-*b*:2',3'-*e*]arsinine (**3b**)

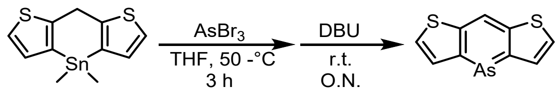

The crude mixture of **2b** was dissolved in benzene (7.5 mL) was added arsenic tribromide (0.523 g, 1.66 mmol) and the mixture was stirred for 3 h at 50 °C. Then, DBU (0.70 mL, 4.7 mmol) was added into the mixture at room temperature, and stirred overnight. the volatiles were removed *in vacuo*. The crude product was washed with ethanol in argon-filled glove box to give the title compounds as red solid (68.2 mg, 0.270 mmol, 18%). ^{1}H-NMR ($CDCl_3$, 400 MHz): δ 8.75 (s, 1H), 7.93 (d, J = 5.4 Hz, 2H), 7.58 (d, J = 5.4 Hz, 2H) ppm; ^{13}C-NMR ($CDCl_3$, 100 MHz): δ 168.9, 141.1, 130.9, 126.3, 119.2 ppm. HR-FAB-MASS (m/z): calculated for $C_9H_5S_2As$ $[M]^+$; 251.9049, observed; 251.9052.

Bis-3-benzo[*b*]thienylmethanol

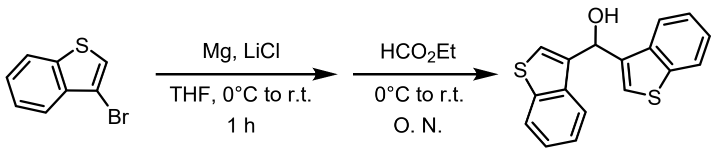

To a suspension of magnesium (0.970 g, 39.9 mmol) and LiCl (1.77 g, 41.8 mmol) in THF (2 mL) was added 1 M THF solution of 3-bromobenzo[*b*]thiophene (5.0 mL, 38 mmol) at 0 °C and the mixture was stirred for 1 h at the same temperature. Then, ethyl formate (1.54 mL, 19.1 mmol) was added into the mixture at 0 °C, and stirred overnight. the mixture was quenched with sat. NH_4Cl *aq.* and extracted with ether. The extracts were dried over anhydrous Na_2SO_4 and after filtration, the volatiles were removed in vacuo. The crude product had sufficient purity, which was used for the next reaction without purification. ^{1}H-NMR ($CDCl_3$, 400 MHz): δ 7.90–7.85 (m, 4H), 7.83–7.79 (m, 4H), 6.53 (d, J = 4.5 Hz, 1H), 2.49 (d, J = 4.6 Hz, 1H) ppm. HR-FAB-MASS (m/z): calculated for $C_{17}H_{12}OS_2$ $[M]^+$; 296.0330, observed; 296.0330.

Bis-3-benzo[*b*]thienylmethane

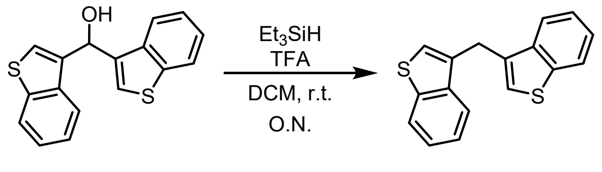

The crude product was dissolved in DCM (70 mL) and Et_3SiH (6.6 mL, 41.4 mmol) was added. The solution was cooled to 0 °C and trifluoroacetic acid (1.6 mL, 20.9 mmol) was added dropwise and stirred overnight at room temperature. The volatiles were removed in vacuo and crude product was passed through the pad of silica eluted with toluene to give the title compound as colorless oil. ^1H-NMR ($CDCl_3$, 400 MHz): δ 7.90–7.85 (m, 4H), 7.77–7.73 (m, 4H), 7.02 (t, J = 1.2 Hz, 2H), 4.38 (t, J = 1.2 Hz, 2H) ppm. HR-FAB-MASS (m/z): calculated for $C_{17}H_{12}S_2$ $[M]^+$; 280.0380, observed; 280.0383.

Bis-3-(2-bromobenzo[*b*]thienyl)methane

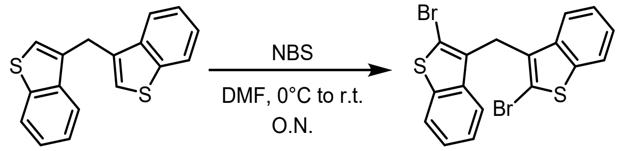

The crude product was dissolved in DMF (160 mL) and the solution was cooled to 0 °C. NBS (7.24 g, 40.7 mmol) was added portionwise at the same temperature and stirred overnight at room temperature. The volatiles were removed *in vacuo* and the residue was washed with ethanol, and the solid was filtered to give the title compound as colorless solid (6.27 g, 14.3 mmol, 75% over 3 steps). ^1H-NMR ($CDCl_3$, 400 MHz): δ 7.68–7.65 (m, 2H), 7.57–7.54 (m, 2H), 7.26–7.18 (m, 4H), 4.43 (s, 2H) ppm; ^{13}C-NMR ($CDCl_3$, 100 MHz): δ 139.6, 138.1, 131.6, 124.7, 124.6, 122.0, 121.7, 114.7, 28.1 ppm. HR-FAB-MASS (m/z): calculated for $C_{17}H_{12}Br_2S_2$ $[M]^+$; 435.8591, observed; 435.8596.

Bis[1]benzothieno[2,3-*b*:3′,2′-*e*]cyclohexastanane (**2c**)

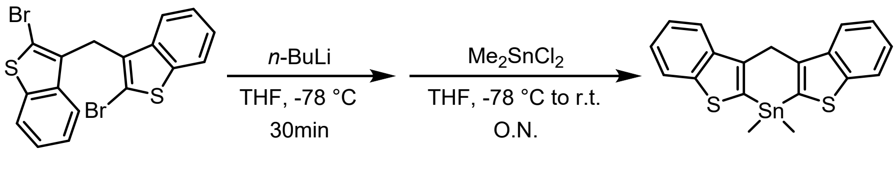

To a solution of bis-3-(2-bromobenzo[b]thienyl)methane (1.50 g, 3.42 mmol) in Et_2O (80 mL) was added *n*-BuLi (1.6 M in hexane, 4.5 mL, 7.2 mmol) dropwise at −78 °C and the mixture was stirred for 30 min at the same temperature. Then, dimethyltin dichloride (0.843 g, 3.84 mmol) in Et_2O (5 mL) was added dropwise into the mixture at the same temperature, and the mixture was warmed to room temperature. After stirring at room temperature overnight, the mixture was quenched with sat. NH_4Cl *aq.* and extracted with DCM. The extracts were dried over anhydrous Na_2SO_4 and after filtration, the volatiles were removed *in vacuo*. The crude product was washed with ethanol to give the title compounds as colorless solid (1.14 g, 2.68 mmol, 78%). ^{1}H-NMR ($CDCl_3$, 400 MHz): δ 8.00 (d, J = 7.9, 2H), 7.94 (d, J = 8.0, 2H), 7.49–7.45 (m, 2H), 7.40–7.36 (m, 2H), 4.69 (s, 2H), 0.66 (s, 6H) ppm; ^{13}C-NMR ($CDCl_3$, 100 MHz): δ 143.9, 139.8, 139.0, 129.1, 124.2, 123.9, 122.4, 121.1, 29.8, −6.7 ppm. HR-FAB-MASS (m/z): calculated for $C_{19}H_{17}S_2Sn$ $[M+H]^+$; 428.9788, observed; 428.9800.

Bis[1]benzothieno[2,3-*b*:3′,2′-*e*]arsinine (**3c**)

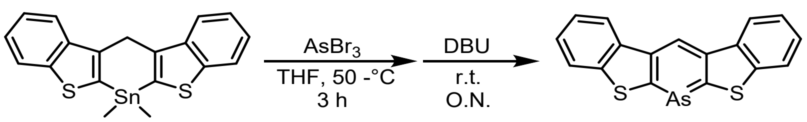

To a solution of **2c** (1.47 g, 3.44 mmol) in THF (35 mL) was added arsenic tribromide (1.14 g, 3.62 mmol) and the mixture was stirred for 3 h at 50 °C. Then, DBU (1.6 mL, 11 mmol) was added into the mixture at room temperature, and stirred overnight. the volatiles were removed *in vacuo*. The crude product was washed with ethanol in air to give the title compounds as red solid (0.810 g, 2.30 mmol, 67%). ^{1}H-NMR ($CDCl_3$, 400 MHz): δ 9.32 (s, 1H), 8.47–8.42 (m, 2H), 7.95–7.7.91 (m, 2H), 7.59–7.52 (m, 4H) ppm; ^{13}C-NMR ($CDCl_3$, 100 MHz): δ 174.5, 141.5, 137.0, 136.1, 127.2, 124.5, 122.3, 121.4, 116.6 ppm. HR-FAB-MASS (m/z): calculated for $C_{17}H_9S_2As$ $[M]^+$; 351.9362, observed; 351.9361.

Bis[1]benzothieno[2,3-*b*:3′,2′-*e*]cyclohexastanane (**2d**)

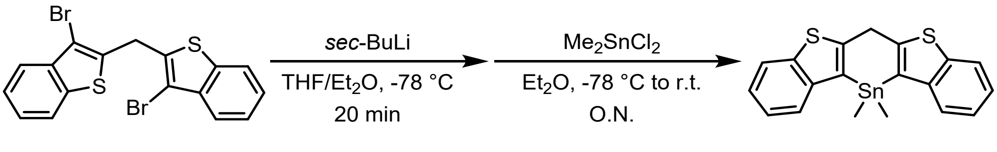

To a solution of bis-2-(3-bromobenzo[*b*]thienyl)methane (0.51 g, 1.16 mmol) in THF (17 mL) and Et_2O (100 mL) was added *sec*-BuLi (1.3 M in hexane, 3.58 mL, 4.65 mmol) dropwise at −78 °C and the mixture was stirred for 20 min at the same temperature. Then, dimethyltin dichloride (0.387 g, 1.76 mmol) in Et_2O (3.5 mL) was added dropwise into the mixture at the

same temperature, and the mixture was stirred for 1 h and warmed to room temperature. After stirring at room temperature overnight, the mixture was quenched with sat. NH_4Cl *aq.* and extracted with DCM. The extracts were dried over anhydrous Na_2SO_4 and after filtration, the volatiles were removed *in vacuo*. The crude product was washed with ethanol to give the title compounds as colorless solid (0.243 g, 0.568 mmol, 49%). ^{1}H-NMR ($CDCl_3$, 400 MHz): δ 7.93–7.83 (m, 2H), 7.68 (d, J = 7.4, 2H), 7.40–7.31 (m, 4H), 4.81 (s, 2H), 0.67 (s, 6H). ppm; ^{13}C-NMR ($CDCl_3$, 100 MHz): δ 146.7, 145.2, 139.8, 129.3, 124.3, 124.2, 124.1, 122.1, 33.3, −8.6 ppm. HR-FAB-MASS (m/z): calculated for $C_{19}H_{17}S_2Sn$ $[M+H]^+$; 428.9788, observed; 428.9787.

Bis[1]benzothieno[2,3-*b*:3′,2′-*e*]arsinine (**3d**)

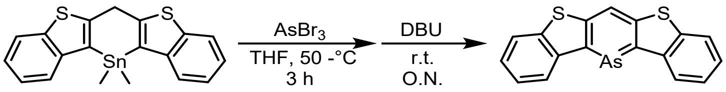

To a solution of **2c** (0.105 g, 0.245 mmol) in THF (2 mL) was added arsenic tribromide (84.8 mg, 0.267 mmol) and the mixture was stirred for 3 h at 50 °C. Then, DBU (0.11 mL, 7.4 mmol) was added into the mixture at room temperature, and stirred overnight. the volatiles were removed *in vacuo*. The crude product was washed with ethanol in air to give the title compounds as red solid (61.8 mg, 0.176 mmol, 72%). ^{1}H-NMR ($CDCl_3$, 400 MHz): δ 8.58 (s, 1H), 8.55–8.53 (m, 2H), 7.89–7.86 (m, 2H), 7.54–7.45 (m, 4H) ppm; ^{13}C-NMR ($CDCl_3$, 100 MHz): δ 166.0, 142.5, 142.0, 138.3, 127.7, 124.8, 122.7, 122.5, 119.2 ppm. HR-FAB-MASS (m/z): calculated for $C_{17}H_9S_2As$ $[M]^+$; 351.9362, observed; 351.9361.

Bis[1]benzothieno[2,3-*b*:3′,2′-*e*]arsinine (**3c**)

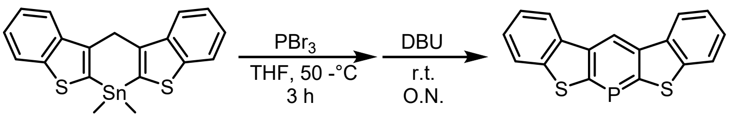

To a solution of **2c** (0.65 g, 1.52 mmol) in THF (8 mL) was added phosphorus tribromide (1.0 M, 1.67 mL, 1.67 mmol) and the mixture was stirred for 3 h at 50 °C. Then, DBU (1.14 mL, 7.62 mmol) was added into the mixture at room temperature, and stirred overnight. the volatiles were removed *in vacuo*. The crude product was washed with ethanol in air to give the title compounds as yellow solid (0.275 g, 0.90 mmol, 59%). ^{1}H-NMR ($CDCl_3$, 400 MHz): δ 9.18 (d, J = 4.1 Hz, 1H), 8.45–8.41 (m, 2H), 7.95–7.91 (m, 2H), 7.59–7.54 (m, 4H) ppm; ^{13}C-NMR ($CDCl_3$, 100 MHz): δ 165.0, 164.4, 141.2, 136.5, 127.6, 124.7, 122.6, 122.6, 121.7, 116.9, 116.7 ppm. ^{31}P-NMR ($CDCl_3$, 162 MHz): δ 151.4 ppm. EI-MASS (m/z): calculated for $C_{17}H_9S_2P$ $[M]^+$; 307.9883, observed; 307.9892.

General procedure of reaction with DMAD.

To a solution of arsinines in benzene (**3a**, **3b**) or toluene (**3c**, **3d**) was added DMAD (1.1 eq.) at 80 °C and the mixture was stirred for overnight. The volatiles were removed *in vacuo*. The crude product was purified by silica gel column chromatography (hexane: EtOAc = 5: 1) to give the title compounds.

3a-DMAD: **3a** (50 mg, 0.20 mmol) and DMAD (26.7 μL, 0.218 mmol) were used. The product was obtained as colorless solid (60.2 mg, 0.153 mmol, 77%). ^{1}H-NMR ($CDCl_3$, 400 MHz): *δ* 7.23–7.20 (m, 4H), 6.08 (s, 1H), 3.80 (s, 3H), 3.79 (s, 3H) ppm; ^{13}C-NMR ($CDCl_3$, 100 MHz): *δ* 168.4, 165.9, 155.1, 154.2, 152.5, 142.4, 129.0, 125.1, 52.8, 52.6, 49.6 ppm. HR-FAB-MASS (m/z): calculated for $C_{15}H_{11}O_4S_2As$ $[M]^+$; 393.9315, observed; 393.9313.

3b-DMAD: **3b** (50 mg, 0.20 mmol) and DMAD (26.7 μL, 0.218 mmol) were used. The product was obtained as colorless solid (56.8 mg, 0.144 mmol, 73%). ^{1}H-NMR ($CDCl_3$, 400 MHz): *δ* 7.22 (d, *J* = 4.9 Hz, 2H), 6.96 (d, *J* = 4.9 Hz, 2H), 6.19 (s, 1H), 3.80 (s, 3H), 3.79 (s, 3H) ppm; ^{13}C-NMR ($CDCl_3$, 100 MHz): *δ* 169.4, 164.5, 157.9, 152.1, 150.0, 144.9, 128.7, 122.6, 52.9, 52.5, 46.7 ppm. HR-FAB-MASS (m/z): calculated for $C_{15}H_{12}O_4S_2As$ $[M+H]^+$; 394.9394, observed; 394.9398.

3c-DMAD: **3c** (50 mg, 0.14 mmol) and DMAD (17.7 μL, 0.145mmol) were used. The product was obtained as colorless solid (58.0 mg, 0.116 mmol, 83%). ^{1}H-NMR ($CDCl_3$, 400 MHz): *δ* 8.09 (d, *J* = 8.1 Hz, 2H), 7.79 (d, *J* = 8.1 Hz, 2H), 7.46–7.42 (m, 2H), 7.35–7.31 (m, 2H), 6.88 (s, 1 H), 3.80 (s, 3H), 3.79 (s, 3H) ppm; ^{13}C-NMR ($CDCl_3$, 100 MHz): *δ* 165.3, 156.1, 150.8, 148.5, 145.8, 143.8, 135.7, 125.0, 124.7, 123.1, 120.9, 53.0, 52.7, 45.3 ppm. HR-FAB-MASS (m/z): calculated for $C_{17}H_9S_2As$ $[M]^+$; 493.9628, observed; 493.9621.

3d-DMAD: **3d** (50 mg, 0.14 mmol) and DMAD (17.7 μL, 0.145mmol) were used. The product was obtained as colorless solid (50.3 mg, 0.100 mmol, 72%). ^{1}H-NMR ($CDCl_3$, 400 MHz): *δ* 7.93 (ddd, *J* = 0.7, 1.1, 8.0 Hz, 2H), 7.77 (dt, *J* = 0.9, 8.1 Hz, 2H), 7.34–7.44 (m, 2H), 7.27-7.34 (m, 2H), 6.27 (s, 1H), 3.82 (s, 3H), 3.80 (s, 3H). ppm; ^{13}C-NMR ($CDCl_3$, 100 MHz): *δ* 168.8, 164.4, 156.5, 153.7, 150.5, 140.9, 140.2, 139.7, 124.9, 124.6, 123.0, 122.8, 53.0, 52.6, 48.3 ppm. HR-FAB-MASS (m/z): calculated for $C_{17}H_9S_2As$ $[M]^+$; 493.9628, observed; 493.9639.

General procedure of reaction with *o*-benzyne

To a solution of arsinines in THF (**3a**, **3c**) (2 mL) was added 2-TMS-benzenetriflate followed by TBAF (1.0 M in THF) at 80 °C and the mixture was stirred for overnight. The volatiles were removed *in vacuo*. The crude product was purified by silica gel column chromatography (hexane) to give the title compounds.

3a-benzyne: **3a** (50 mg, 0.20 mmol), 2-TMS-benzenetriflate (35.1 μL, 0.145 mmol), and TBAF solution (1.0 M in THF, 0.15 mL, 0.15 mmol) were used. The product was obtained as colorless solid (50.4 mg, 0.115 mmol, 82%). ^{1}H-NMR ($CDCl_3$, 400 MHz): δ 7.69 (d, J = 7.1 Hz, 1H), 7.46 (d, J = 7.3 Hz, 1H), 7.20 (d, J = 4.7 Hz, 2H), 7.15 (d, J = 4.7 Hz, 2H), 7.11 (td, J = 7.4, 1.2 Hz, 1H), 6.98 (td, J = 7.4, 1.2 Hz, 1H) 5.88 (s, 1H) ppm; ^{13}C-NMR ($CDCl_3$, 100 MHz): δ 154.9, 148.3, 145.8, 142.6, 131.6, 128.0, 127.9, 126.4, 125.1, 124.5, 52.7 ppm. HR-FAB-MASS (m/z): calculated for $C_{15}H_9S_2As$ $[M]^+$; 327.9362, observed; 327.9367.

3c-benzyne: **3c** (50 mg, 0.14 mmol), 2-TMS-benzenetriflate (50.5 μL, 0.208 mmol), and TBAF solution (1.0 M in THF, 0.24 mL, 0.24 mmol) were used. The product was obtained as colorless solid (50.4 mg, 0.118 mmol, 83%). ^{1}H-NMR ($CDCl_3$, 400 MHz): δ 8.11 (d, J = 8.1 Hz, 2H), 7.78–7.75 (m, 3H), 7.62 (dd, J = 7.3, 1.0 Hz, 1H), 7.45–7.41 (m, 2H), 7.31–7.27 (m, 2H), 7.13 (td, J = 7.4, 1.3 Hz, 1H), 7.01 (td, J = 7.3, 1.2 Hz, 1H), 6.53 (s, 1H) ppm; ^{13}C-NMR ($CDCl_3$, 100 MHz): δ 148.7, 147.1, 146.1, 145.3, 143.5, 136.0, 131.9, 128.2, 126.3, 124.8, 124.6, 124.3, 123.0, 120.5, 48.0 ppm. HR-FAB-MASS (m/z): calculated for $C_{23}H_{13}S_2As$ $[M]^+$; 427.9675, observed; 427.9686.

3c-W(CO)$_5$

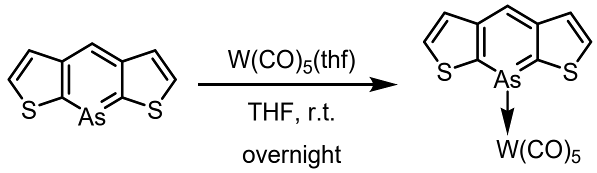

To a solution of $W(CO)_5$(thf) prepared by the UV irradiation of solution of $W(CO)_5$ (98.0 mg, 0.278 mmol) in THF (10 mL) was added **3c** (70.0 mg, 0.278 mmol) and the mixture was stirred overnight. The volatiles were removed *in vacuo* and the crude product was recrystallization from THF/ethanol in argon-filled glovebox to give the title compounds as red solid (115 mg, 0.200 mmol, 72%). ^{1}H-NMR (THF-d_6, 400 MHz): δ 8.73 (s, 1H), 7.86 (d, J = 5.3 Hz, 2H), 7.70 (d, J = 5.3 Hz, 2H) ppm; ^{13}C-NMR (THF-d_6, 100 MHz): δ 199.0, 191.3, 171.8, 141.4, 129.6, 126.9, 122.4 ppm. HR-FAB-MASS (m/z): calculated for $C_{14}H_5O_5S_2AsW$ $[M]^+$; 575.8304, observed; 575.8303.

3d’

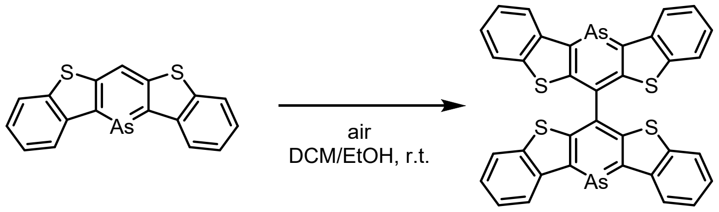

Solution of **3d** was dissolved in DCM and ethanol was layered on the solution. The mixture was left for 2 days to give the brown needle crystals. ^{1}H-NMR ($CDCl_3$, 400 MHz): δ 8.69–8.67 (m, 4H), 7.68–7.65 (m, 4H), 7.54–7.45 (m, 8H) ppm; HR-FAB-MASS (m/z): calculated for $C_{34}H_{16}S_4As_2$ $[M]^+$; 701.8567, observed; 701.8579.

3d-P’

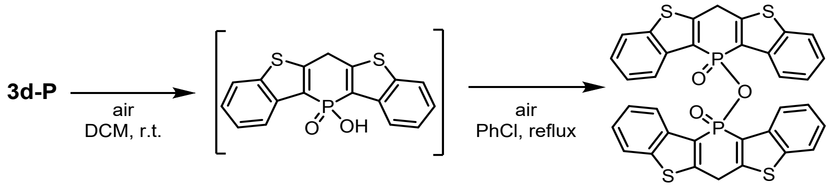

3d-P'

Solution of **3d-P** was dissolved in DCM and left for overnight to give the insoluble yellow powder, which was phosphinic acid. MALDI-TOF MS (m/z): calculated for $C_{17}H_9O_2S_2P$ $[M-H]^-$; 340.9865 observed; 340.9873.

The powder was dissolved by hot chlorobenzene and cooled to ambient temperature to give the yellow crystal. MALDI-TOF MS (m/z): calculated for $C_{34}H_{20}O_3NaS_4P_2$ $[M+Na]^+$; 688.9663, observed; 688.9650

4. X-ray diffraction analysis

The single crystal was mounted on a glass fiber. Intensity data were collected at 100 K on a Rigaku XtaLAB mini with graphite-monochromated Mo *Kα* radiation. Readout was performed in 0.073 mm pixel mode. The data were collected to a maximum 2θ value of 52.7°. Data were processed using CrysAlisPro.[6] An analytical numeric absorption correction[7] was applied. The data were corrected for Lorentz and polarization effects. The structure was solved by the ShelXT[8] and expanded using Fourier techniques. Non-hydrogen atoms were refined anisotropically. Hydrogen atoms were refined using the riding model. The final cycle of full-matrix least-squares refinement on F^2 was based on observed reflections and variable parameters. All calculations were performed using the Olex2[9] crystallographic software package except for refinement, which was performed using SHELXL2016.[10]

Deposition Number 2385300 (**3a**), 2385301 (**3b**), 2385302 (**3c**), 2385303 (**3d**-plate), 2385304 (**3d**-needle), 2385305 (**3c-P**), 2385306 (**3b-DMAD**), 2385307 (**3c-DMAD**), 2385308 (**3c-benzyne**), 2385309 (**3a-WO(CO)$_5$**), 2385310 (**3d'**), and 2385311 (**3d-P'**) contain the supplementary crystallographic data for this paper. These data are provided free of charge by the joint Cambridge Crystallographic Data Centre and Fachinformationszentrum Karlsruhe Access Structures service www.ccdc.cam.ac.uk/structures.

Table S1. Crystallographic Data of **3a-c**.

	**3a**	**3b**	**3c**
Crystal data			
Empirical formula	$C_9H_5AsS_2$	$C_9H_5AsS_2$	$C_{17}H_9AsS_2$
Formula weight	252.17	252.17	352.28
Crystal size / mm³	0.848×0.236×0.192	0.848×0.477×0.289	0.514×0.392×0.098
Crystal system	monoclinic	monoclinic	monoclinic
Space group	$P2_1/n$	Pn	C2/c
a /Å	10.5045(11)	6.1248(3)	13.4892(12)
b /Å	6.2032(5)	11.3296(6)	8.5408(7)
c /Å	13.9819(13)	12.9122(7)	23.219(2)
α /°	90	90	90
β /°	102.093(10)	95.535(6)	97.422(9)
γ /°	90	90	90
Volume /Å³	890.86(15)	891.82(8)	2652.6(4)
ρ_{calc} g/cm³	1.880	1.878	1.764
Z	4	4	8
F(000)	496	496	1408
Data Collection			
Temperature /K	197.15	100	100
2θmax /°	52.744	55.114	52.74
Tmin/Tmax	0.261/0.476	0.588/0.766	0.888/0.966
Refinement			
No. of Observed Data	1822	2998	2691
No. of Parameters	109	316	181
R1[a], wR2[b]	0.0363, 0.1018	0.0299, 0.0737	0.0461, 0.1027
Goodness of Fit Indictor	1.079	1.037	0.993

[a]R1 = Σ | |Fo| - |Fc| | / Σ |Fo| [b]wR2 = [Σ w ((Fo^2-Fc^2)^2 / Σ w (Fo^2)^2]$^{1/2}$ w = [σ^2(Fo^2)]$^{-1}$

CCDC 2385300 (**3a**), 2385301 (**3b**), 2385302 (**3c**).

Table S2 Crystallographic Data of **3d-plate**, **3d-needle**, and **3c-P**.

	**3d-plate**	**3d-needle**	**3c-P**
Crystal data			
Empirical formula	$C_{17}H_9AsS_2$	$C_{17}H_9AsS_2$	$C_{17}H_9PS_2$
Formula weight	352.28	352.28	308.33
Crystal size / mm^3	0.347×0.19×0.077	0.468×0.058×0.038	0.539×0.292×0.091
Crystal system	monoclinic	monoclinic	monoclinic
Space group	$P2_1/c$	$P2_1/c$	C2/c
a /Å	13.1348(11)	14.5703(9)	13.2423(8)
b /Å	8.5422(5)	3.9421(3)	8.4253(6)
c /Å	12.1986(10)	23.8017(18)	23.5820(15)
α /°	90	90	90
β /°	93.983(8)	99.317(7)	97.092(6)
γ /°	90	90	90
Volume /$Å^3$	1365.38(18)	1349.08(17)	2610.9(3)
ρ_{calc} g/cm^3	1.714	1.734	1.569
Z	4	4	8
F(000)	704	704	1264
Data Collection			
Temperature /K	100	100.00	100.00
2θmax /°	52.738	52.744	52.74
Tmin/Tmax	0.904/0.974	0.864/0.976	0.984/0.997
Refinement			
No. of Observed Data	2805	2741	2672
No. of Parameters	350	181	181
R1^a, wR2^b	0.0622, 0.1643	0.0503, 0.1369	0.0359, 0.0963
Goodness of Fit Indictor	1.112	1.053	1.096

^{a}R1 = Σ \| \|Fo\| - \|Fc\| \| / Σ \|Fo\| ^{b}wR2 = $[\Sigma w ((Fo^2-Fc^2)^2 / \Sigma w (Fo^2)^2]^{1/2}$ $w = [\sigma^2(Fo^2)]^{-1}$

CCDC 2385303 (**3d**-plate), 2385304 (**3d**-needle), 2385305 (**3c-P**).

Table S3 Crystallographic Data of **3a-W(CO)$_5$**, **3b-DMAD**, and **3c-DMAD**.

	**3b-DMAD**	**3c-DMAD**	**3c-benzyne**
Crystal data			
Empirical formula	$C_{15}H_{11}AsO_4S_2$	$C_{23}H_{15}AsO_4S_2$	$C_{23}H_{13}AsS_2$
Formula weight	394.28	494.39	428.37
Crystal size / mm³	0.514×0.149×0.137	0.547×0.285×0.083	0.54×0.33×0.15
Crystal system	monoclinic	orthorhombic	monoclinic
Space group	$P2_1/c$	Pnma	I2/a
a /Å	10.4030(4)	17.8326(12)	16.1897(5)
b /Å	14.0494(5)	7.7360(6)	7.1373(3)
c /Å	10.7101(4)	30.2340(15)	31.4478(9)
α /°	90	90	90
β /°	99.246(3)	90	90.732(3)
γ /°	90	90	90
Volume /Å³	1545.01(10)	4170.9(5)	3633.5(2)
ρ_{calc} g/cm³	1.695	1.575	1.566
Z	4	8	8
F(000)	792	2000	1728
Data Collection			
Temperature /K	100.00	100.00	100.00
2θmax /°	52.74	52.738	52.746
Tmin/Tmax	0.019/0.095	0.910/0.977	0.015/0.122
Refinement			
No. of Observed Data	3150	5488	3705
No. of Parameters	201	546	235
R1[a], wR2[b]	0.0328, 0.0931	0.0412, 0.0990	0.0345, 0.0919
Goodness of Fit Indictor	1.034	1.056	1.084

[a]R1 = Σ | |Fo| - |Fc| | / Σ |Fo| [b]wR2 = $[\ \Sigma\ w\ ((Fo^2-Fc^2)^2\ /\ \Sigma\ w\ (Fo^2)^2\ ]^{1/2}$ w = $[\ \sigma^2(Fo^2)\ ]^{-1}$

CCDC 2385306 (**3b-DMAD**), 2385307 (**3c-DMAD**), 2385308 (**3c-benzyne**).

Table S4 Crystallographic Data of **3c-benzyne**, **3d'**, and **3d-P'**.

	**3a-W(CO)$_5$**	**3d'**	**3d-P'**
Crystal data			
Empirical formula	$C_{14}H_5AsO_5S_2W$	$C_{34}H_{16}As_2S_4$	$C_{34}H_{20}O_3P_2S_4$
Formula weight	576.07	702.55	666.68
Crystal size / mm³	0.423×0.282×0.091	0.608×0.129×0.055	0.45×0.14×0.07
Crystal system	triclinic	triclinic	triclinic
Space group	P-1	P-1	P-1
a /Å	7.3180(12)	8.0954(6)	7.4187(3)
b /Å	10.2845(16)	11.3660(7)	13.2453(5)
c /Å	11.3383(10)	15.4655(8)	17.9755(7)
α /°	92.150(10)	87.822(5)	77.315(3)
β /°	107.144(11)	85.309(5)	81.898(4)
γ /°	100.322(13)	80.114(6)	76.885(4)
Volume /Å³	798.5(2)	1396.77(16)	1670.53(12)
ρ_{calc} g/cm³	2.396	1.670	1.325
Z	2	2	2
F(000)	536	700	684
Data Collection			
Temperature /K	100	100.00	180.15
2θmax /°	52.906	52.746	152.766
Tmin/Tmax	0/1	0.811/0.968	0.231/0.739
Refinement			
No. of Observed Data	3159	5695	6406
No. of Parameters	208	361	388
R1^a, wR2^b	0.0783, 0.2094	0.0421, 0.1076	0.1064, 0.3047
Goodness of Fit Indictor	1.025	1.016	1.048

^{a}R1 = Σ | |Fo| - |Fc| | / Σ |Fo| ^{b}wR2 = [Σ w ((Fo^2-Fc^2)^2 / Σ w (Fo^2)^2]$^{1/2}$ w = [σ^2(Fo^2)]$^{-1}$

CCDC 2385309 (**3a-WO(CO)$_5$**), 2385310 (**3d'**), 2385311 (**3d-P'**).

Table S5. ORTEP drawing (ellipsoids at 50% probability), and selected bond lengths and angles of **3a**. All hydrogens of the ligands are omitted for clarity.

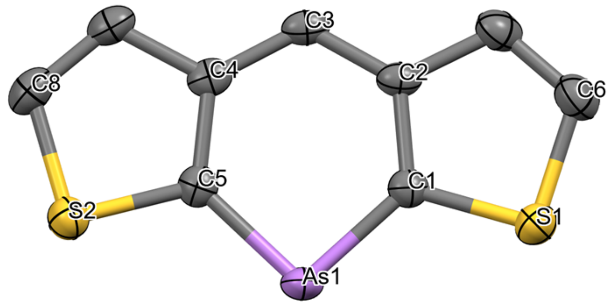

Distances (Å)			Angles (°)			
Atom1	Atom2	Length	Atom1	Atom2	Atom3	Angle
As1	C1	1.860(3)	C1	As1	C5	94.7(1)
As1	C5	1.859(3)	As1	C1	C2	127.1(2)
C1	C2	1.422(4)	C1	C2	C3	124.1(3)
C2	C3	1.393(5)	C2	C3	C4	123.2(3)
C3	C4	1.407(4)	C3	C4	C5	123.7(3)
C4	C5	1.424(4)	C4	C5	As1	127.1(2)
S1	C1	1.728(3)	C1	S1	C6	91.9(2)
S1	C6	1.736(4)	C5	S2	C8	92.2(2)
S2	C5	1.738(3)				
S2	C8	1.727(4)				

Table S6. ORTEP drawing (ellipsoids at 50% probability), and selected bond lengths and angles of **3b**. All hydrogens of the ligands are omitted for clarity. The major part was shown.

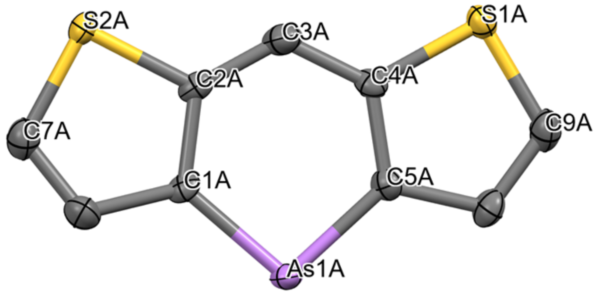

Distances (Å)		
Atom1	Atom2	Length
As1A	C1A	1.88(2)
As1A	C5A	1.87(1)
C1A	C2A	1.41(2)
C2A	C3A	1.36(1)
C3A	C4A	1.37(2)
C4A	C5A	1.41(2)
S2A	C2A	1.75(1)
S2A	C7A	1.73(2)
S1A	C4A	1.75(1)
S1A	C9A	1.73(2)

Angles (°)			
Atom1	Atom2	Atom3	Angle
C1A	As1A	C5A	96.8(6)
As1A	C1A	C2A	124(1)
C1A	C2A	C3A	126(1)
C2A	C3A	C4A	125.1(9)
C3A	C4A	C5A	125(1)
C4A	C5A	As1A	124(1)
C2A	S2A	C7A	91.2(6)
C4A	S1A	C9A	91.7(6)

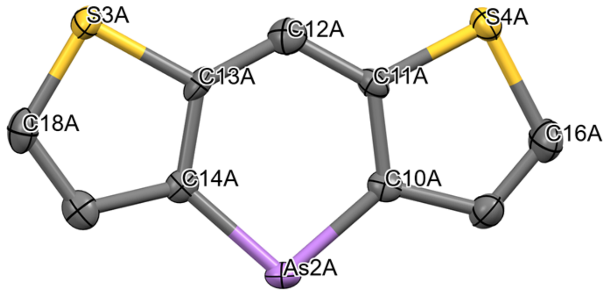

Distances (Å)		
Atom1	Atom2	Length
As2A	C10A	1.86(2)
As2A	C14A	1.87(1)
C10A	C11A	1.42(2)
C11A	C12A	1.36(2)
C12A	C13A	1.39(2)
C13A	C14A	1.42(2)
S3A	C13A	1.76(1)
S3A	C18A	1.72(2)
S4A	C11A	1.76(2)
S4A	C16A	1.80(2)

Angles (°)			
Atom1	Atom2	Atom3	Angle
C10A	As2A	C14A	97.7(6)
As2A	C10A	C11A	124(1)
C10A	C11A	C12A	126(1)
C11A	C12A	C13A	123(1)
C12A	C13A	C14A	127(1)
C13A	C14A	As2A	122(1)
C13A	S3A	C18A	91.9(7)
C11A	S4A	C16A	90.1(7)

Table S7. ORTEP drawing (ellipsoids at 50% probability), and selected bond lengths and angles of **3c**. All hydrogens of the ligands are omitted for clarity.

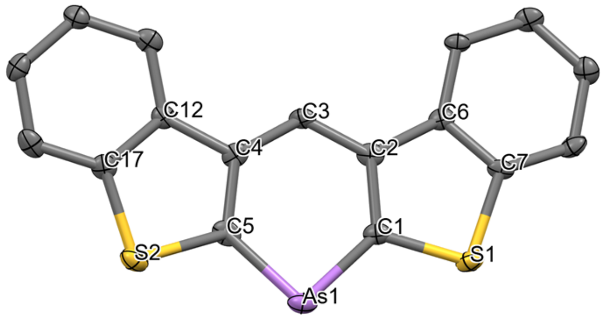

Distances (Å)			Angles (°)			
Atom1	Atom2	Length	Atom1	Atom2	Atom3	Angle
As1	C5	1.863(4)	C1	As1	C5	94.7(2)
As1	C1	1.853(5)	As1	C1	C2	128.0(3)
C1	C2	1.430(6)	C1	C2	C3	122.4(4)
C2	C3	1.398(6)	C2	C3	C4	123.9(4)
C3	C4	1.391(7)	C3	C4	C5	124.7(4)
C4	C5	1.421(6)	C4	C5	As1	126.2(3)
C5	As1	1.863(4)	C1	S1	C7	92.5(2)
S1	C1	1.756(4)	C5	S2	C17	92.2(2)
S1	C7	1.757(5)				
S2	C5	1.743(5)				
S2	C17	1.762(4)				

Table S8. ORTEP drawing (ellipsoids at 50% probability), and selected bond lengths and angles of **3d-needle**. All hydrogens of the ligands were omitted for clarity.

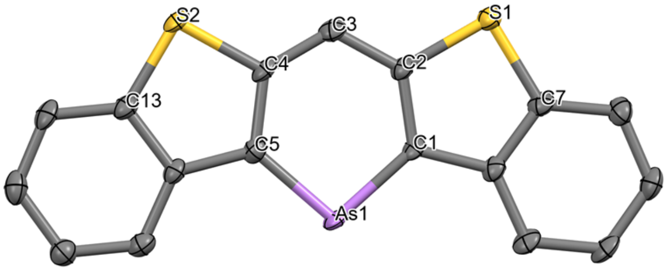

Distances (Å)			Angles (°)			
Atom1	Atom2	Length	Atom1	Atom2	Atom3	Angle
As1	C1	1.857(5)	C1	As1	C5	97.4(2)
As1	C5	1.859(4)	As1	C1	C2	123.7(3)
C1	C2	1.420(7)	C1	C2	C3	126.9(4)
C2	C3	1.392(6)	C2	C3	C4	121.2(4)
C3	C4	1.388(7)	C3	C4	C5	126.9(4)
C4	C5	1.415(7)	C4	C5	As1	124.0(3)
S1	C2	1.769(5)	C2	S1	C7	91.6(2)
S1	C7	1.752(5)	C4	S2	C13	91.7(2)
S2	C4	1.763(4)				
S2	C13	1.742(5)				

Table S9. ORTEP drawing (ellipsoids at 50% probability), and selected bond lengths and angles of **3d-plate**. All hydrogens of the ligands are omitted for clarity. The major part was shown.

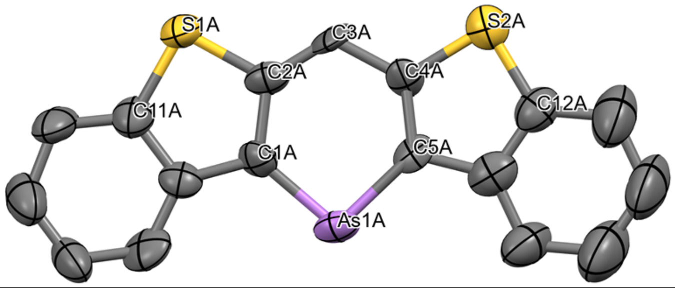

Distances (Å)			Angles (°)			
Atom1	Atom2	Length	Atom1	Atom2	Atom3	Angle
As1A	C1A	1.86(1)	C1A	As1A	C5A	96.3(8)
As1A	C5A	1.89(2)	As1A	C1A	C2A	124(1)
C1A	C2A	1.44(2)	C1A	C2A	C3A	128(1)
C2A	C3A	1.36(2)	C2A	C3A	C4A	121(1)
C3A	C4A	1.47(2)	C3A	C4A	C5A	125(1)
C4A	C5A	1.37(3)	C4A	C5A	As1A	126(1)
S1A	C2A	1.75(1)	C2A	S1A	C11A	91.0(6)
S1A	C11A	1.76(1)	C4A	S2A	C12A	90.1(5)
S2A	C4A	1.73(1)				
S2A	C12A	1.71(1)				

Table S10. ORTEP drawing (ellipsoids at 50% probability), and selected bond lengths and angles of **3c-P**. All hydrogens of the ligands are omitted for clarity.

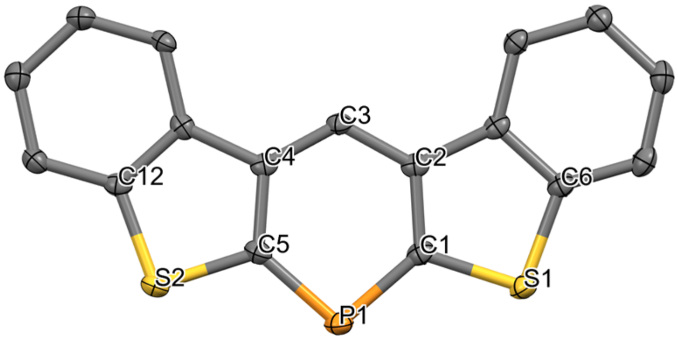

Distances (Å)			Angles (°)			
Atom1	Atom2	Length	Atom1	Atom2	Atom3	Angle
P1	C1	1.739(2)	C1	P1	C5	97.63(9)
P1	C5	1.740(2)	P1	C1	C2	127.6(1)
C1	C2	1.417(3)	C1	C2	C3	122.2(2)
C2	C3	1.395(3)	C2	C3	C4	122.6(2)
C3	C4	1.387(3)	C3	C4	C5	122.4(2)
C4	C5	1.416(3)	C4	C5	P1	127.6(1)
S1	C1	1.755(2)	C1	S1	C6	92.26(9)
S1	C6	1.750(2)	C5	S2	C12	92.11(9)
S2	C5	1.752(2)				
S2	C12	1.744(2)				

Table S11. ORTEP drawing (ellipsoids at 50% probability), and selected bond lengths and angles of **3b-DMAD**. All hydrogens of the ligands are omitted for clarity.

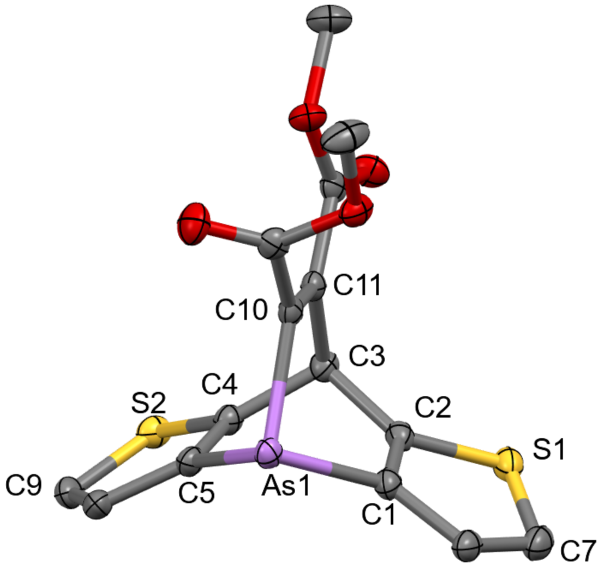

Distances (Å)			Angles (°)			
Atom1	Atom2	Length	Atom1	Atom2	Atom3	Angle
As1	C1	1.966(2)	C1	As1	C5	92.11(9)
As1	C5	1.956(2)	As1	C1	C2	115.4(2)
C1	C2	1.365(3)	C1	C2	C3	121.7(2)
C2	C3	1.519(3)	C2	C3	C4	107.4(2)
C3	C4	1.518(3)	C3	C4	C5	121.2(2)
C4	C5	1.359(3)	C4	C5	As1	116.2(2)
S1	C2	1.713(2)	C2	S1	C7	91.5(1)
S1	C7	1.732(3)	C4	S2	C9	91.4(1)
S2	C4	1.718(2)	C1	As1	C10	89.55(9)

S2	C9	1.713(3)	C5	As1	C10	89.37(9)
As1	C10	2.022(2)	As1	C10	C11	117.2(2)
C10	C11	1.337(3)	C10	C11	C3	119.0(2)
C11	C3	1.545(3)				

8

Table S12. ORTEP drawing (ellipsoids at 50% probability), and selected bond lengths and angles of **3c-DMAD**. All hydrogens of the ligands are omitted for clarity.

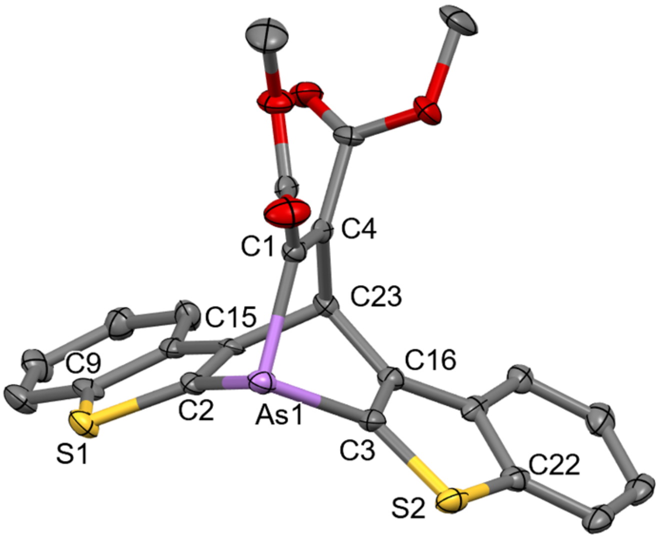

Distances (Å)			Angles (°)			
Atom1	Atom2	Length	Atom1	Atom2	Atom3	Angle
As1	C1	2.007(6)	C1	As1	C2	88.8(3)
As1	C2	1.953(7)	C1	As1	C3	89.5(3)
As1	C3	1.971(6)	C2	As1	C3	89.6(3)
C2	C15	1.36(1)	As1	C2	C15	118.4(5)
C15	C23	1.516(8)	C2	C15	C23	118.8(6)
C23	C16	1.533(8)	C15	C23	C16	108.0(5)
C16	C3	1.35(1)	C23	C16	C3	118.7(5)
S1	C2	1.739(6)	C16	C3	As1	118.2(5)
S1	C9	1.757(7)	C2	S1	C9	91.4(3)

S2	C3	1.729(6)	C3	S2	C22	90.8(3)
S2	C22	1.758(7)	As1	C1	C4	117.2(5)
C1	C4	1.33(1)	C1	C4	C23	119.8(6)
C4	C23	1.528(9)				

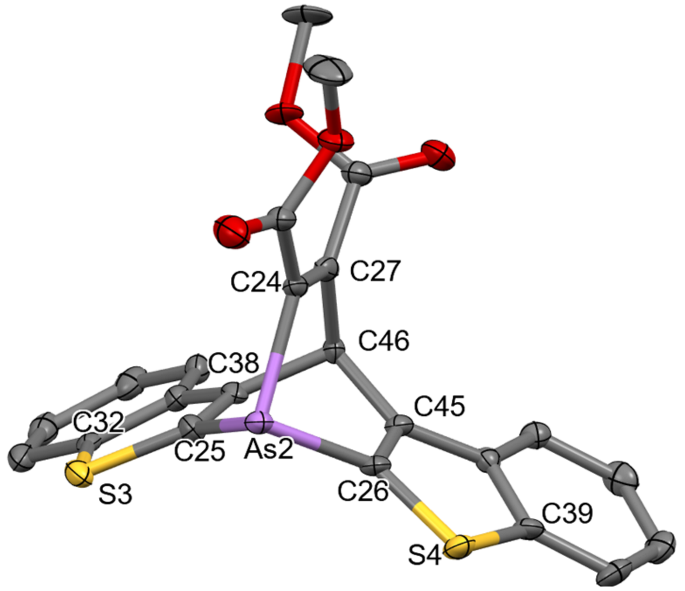

Distances (Å)			Angles (°)			
Atom1	Atom2	Length	Atom1	Atom2	Atom3	Angle
As2	C24	2.017(6)	C24	As2	C25	89.4(3)
As2	C25	1.958(6)	C24	As2	C26	89.4(3)
As2	C26	1.961(6)	C25	As2	C26	89.4(3)
C25	C38	1.36(1)	As2	C25	C38	119.1(5)
C38	C46	1.526(8)	C25	C38	C46	117.8(5)

C46	C45	1.520(8)	C38	C46	C45	108.4(5)
C45	C26	1.36(1)	C46	C45	C26	118.4(5)
S3	C25	1.741(6)	C45	C26	As2	118.5(5)
S3	C32	1.748(8)	C25	S3	C32	90.8(3)
S4	C26	1.731(6)	C26	S4	C39	91.2(3)
S4	C39	1.770(7)	As2	C24	C27	116.5(5)
C24	C27	1.34(1)	C24	C27	C46	120.0(6)
C27	C46	1.538(9)				

Table S13. ORTEP drawing (ellipsoids at 50% probability), and selected bond lengths and angles of **3c-benzyne**. All hydrogens of the ligands are omitted for clarity.

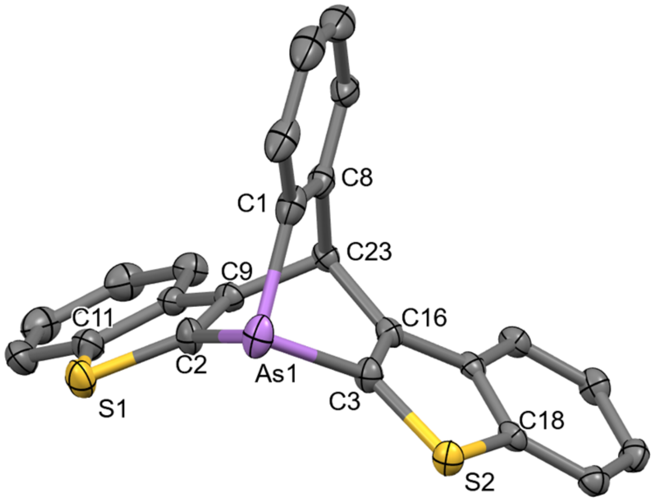

Distances (Å)			Angles (°)			
Atom1	Atom2	Length	Atom1	Atom2	Atom3	Angle
As1	C1	1.975(3)	C1	As1	C2	89.3(1)
As1	C2	1.962(3)	C1	As1	C3	89.9(1)
As1	C3	1.956(2)	C2	As1	C3	89.4(1)
C2	C9	1.361(4)	As1	C2	C9	118.5(2)
C9	C23	1.521(3)	C2	C9	C23	118.5(2)
C23	C16	1.522(3)	C9	C23	C16	108.2(2)
C16	C3	1.358(3)	C23	C16	C3	118.6(2)
S1	C2	1.729(3)	C16	C3	As1	118.7(2)
S1	C11	1.743(3)	C2	S1	C11	91.6(1)
S2	C3	1.730(2)	C3	S2	C18	91.2(1)

S2	C18	1.745(2)
C1	C8	1.397(4)
C8	C23	1.534(3)

As1	C1	C8	118.1(2)
C1	C8	C23	117.1(2)

Table S14. ORTEP drawing (ellipsoids at 50% probability), and selected bond lengths and angles of **3a-W(CO)₅**. All hydrogens of the ligands are omitted for clarity.

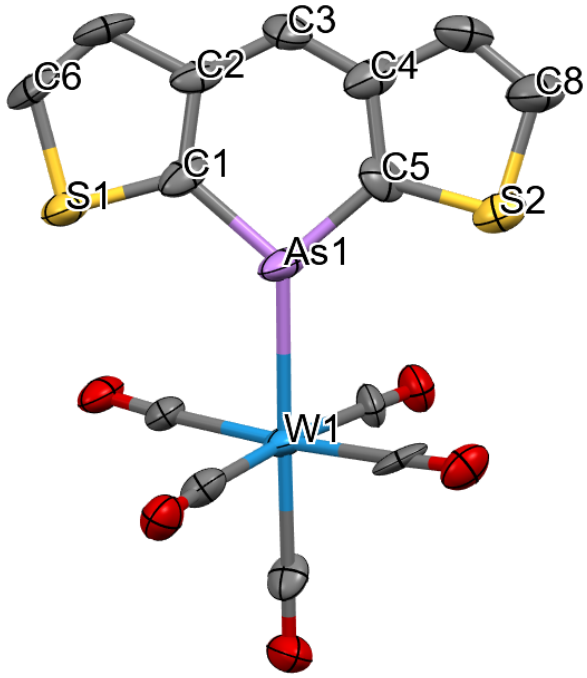

Distances (Å)			Angles (°)			
Atom1	Atom2	Length	Atom1	Atom2	Atom3	Angle
As1	W1	2.586(2)	C1	As1	C5	98.1(9)
As1	C1	1.87(2)	C1	As1	W1	128.6(6)
As1	C5	1.84(2)	C5	As1	W1	128.9(6)
C1	C2	1.41(3)	As1	C1	C2	124(1)
C2	C3	1.43(2)	C1	C2	C3	124(2)
C3	C4	1.34(3)	C2	C3	C4	124(2)
C4	C5	1.43(3)	C3	C4	C5	126(2)
S1	C1	1.72(2)	C4	C5	As1	124(1)

S1	C6	1.74(2)
S2	C5	1.72(2)
S2	C8	1.76(2)
W1	C10	2.00(2)
W1	C11	2.04(2)
W1	C12	2.02(2)
W1	C13	2.07(2)
W1	C14	2.05(2)

C1	S1	C6	89.9(9)
C5	S2	C8	91(1)
As1	W1	C10	177.4(6)
As1	W1	C11	88.3(5)
As1	W1	C12	96.2(5)
As1	W1	C13	87.5(5)
As1	W1	C14	88.2(5)

Table S15. ORTEP drawing (ellipsoids at 50% probability), and selected bond lengths and angles of **3d'**. All hydrogens of the ligands are omitted for clarity.

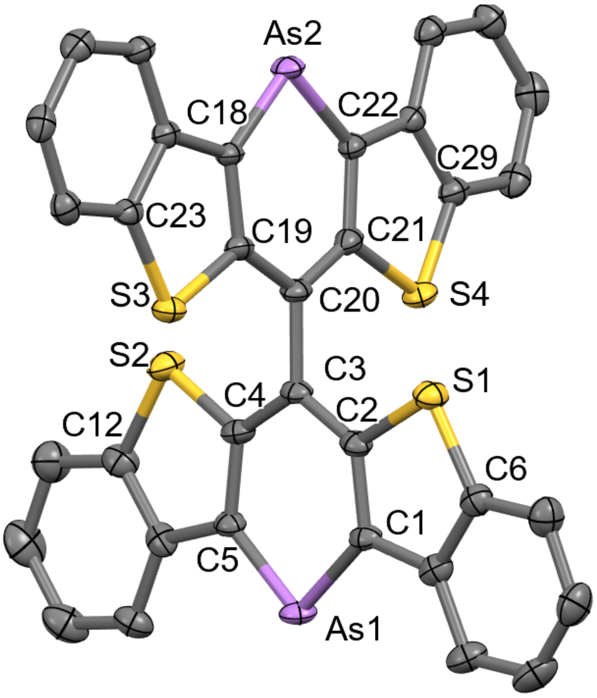

Distances (Å)			Angles (°)			
Atom1	Atom2	Length	Atom1	Atom2	Atom3	Angle
As1	C1	1.860(3)	C1	As1	C5	96.5(2)
As1	C5	1.851(4)	As1	C1	C2	125.1(3)
C1	C2	1.408(5)	C1	C2	C3	126.4(3)
C2	C3	1.390(5)	C2	C3	C4	120.5(3)
C3	C4	1.401(5)	C3	C4	C5	127.2(3)
C4	C5	1.408(4)	C4	C5	As1	124.4(3)
S1	C2	1.762(3)	C2	S1	C6	92.0(2)
S1	C6	1.750(4)	C4	S2	C12	91.6(2)

S2	C4	1.757(4)
S2	C12	1.745(3)
As2	C18	1.866(3)
As2	C22	1.867(4)
C18	C19	1.417(5)
C19	C20	1.401(5)
C20	C21	1.392(5)
C21	C22	1.415(5)
S3	C19	1.761(4)
S3	C23	1.742(4)
S4	C21	1.760(4)
S4	C29	1.749(3)

C18	As2	C22	97.4(2)
As2	C18	C19	123.8(3)
C18	C19	C20	126.8(3)
C19	C20	C21	120.9(3)
C20	C21	C22	127.4(3)
C21	C22	As2	123.5(3)
C19	S3	C23	91.9(2)
C21	S4	C29	91.6(2)

Table S16. ORTEP drawing (ellipsoids at 50% probability), and selected bond lengths and angles of **3d-P’**. All hydrogens of the ligands are omitted for clarity.

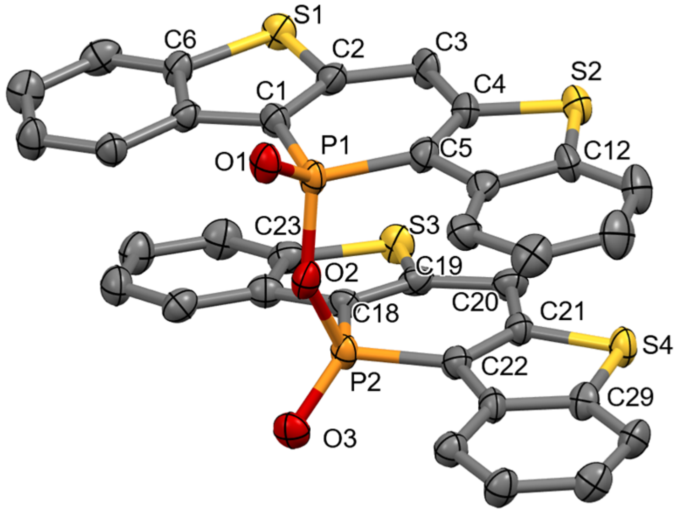

Distances (Å)			Angles (°)			
Atom1	Atom2	Length	Atom1	Atom2	Atom3	Angle
P1	O1	1.460(5)	O1	P1	O2	107.7(3)
P1	O2	1.611(4)	O1	P1	C1	116.1(3)
P1	C1	1.765(7)	O1	P1	C5	115.9(3)
P1	C5	1.750(8)	C1	P1	C5	104.0(3)
C1	C2	1.37(1)	C5	P1	O2	105.2(3)
C2	C3	1.47(1)	C1	P1	O2	107.1(3)
C3	C4	1.50(1)	P1	C1	C2	121.3(5)
C4	C5	1.368(9)	C1	C2	C3	129.2(6)
S1	C2	1.735(6)	C2	C3	C4	114.7(6)
S1	C6	1.754(7)	C3	C4	C5	127.5(6)

S2	C4	1.739(8)
S2	C12	1.730(7)
P2	O3	1.467(5)
P2	O2	1.621(4)
P2	C18	1.763(6)
P2	C22	1.771(8)
C18	C19	1.35(1)
C19	C20	1.51(1)
C20	C21	1.509(9)
C21	C22	1.37(1)
S3	C19	1.741(6)
S3	C23	1.726(8)
S4	C21	1.730(7)
S4	C29	1.737(6)

C4	C5	P1	122.8(5)
C2	S1	C6	91.4(3)
C4	S2	C12	91.4(3)
O3	P2	O2	107.3(3)
O3	P2	C22	116.2(3)
O3	P2	C18	116.2(3)
C22	P2	O3	116.2(3)
C22	P2	C18	103.3(3)
P2	C18	C19	123.8(5)
C18	C19	C20	129.0(6)
C19	C20	C21	111.8(5)
C20	C21	C22	130.3(6)
C21	C22	P2	121.4(5)
C19	S3	C23	91.6(3)
C21	S4	C29	91.7(3)
C22	P2	O2	106.8(3)
C18	P2	O2	106.3(3)

The solid samples of **3a**, **3b**, and **3d** were non-emissive. The packing structures of **3b** and **3d-plate** exhibited a sandwich herringbone arrangement, stacked through As···C interactions. **3a** and **3d-needle** displayed 1D-columnar packing, where As···As interactions were also observed. However, it was suggested that the As lone pair and σ*(As–C) interaction occurred in a loosely packed arrangement. Despite the presence of arsenic-related interactions, no orbital interactions conducive to solid-state emission were detected. Given that the compounds synthesized in this study are intrinsically non-emissive due to symmetry-forbidden transitions, it is likely that these compounds, lacking the interactions found in **3c**, did not exhibit solid-state emission (Φ < 0.01).

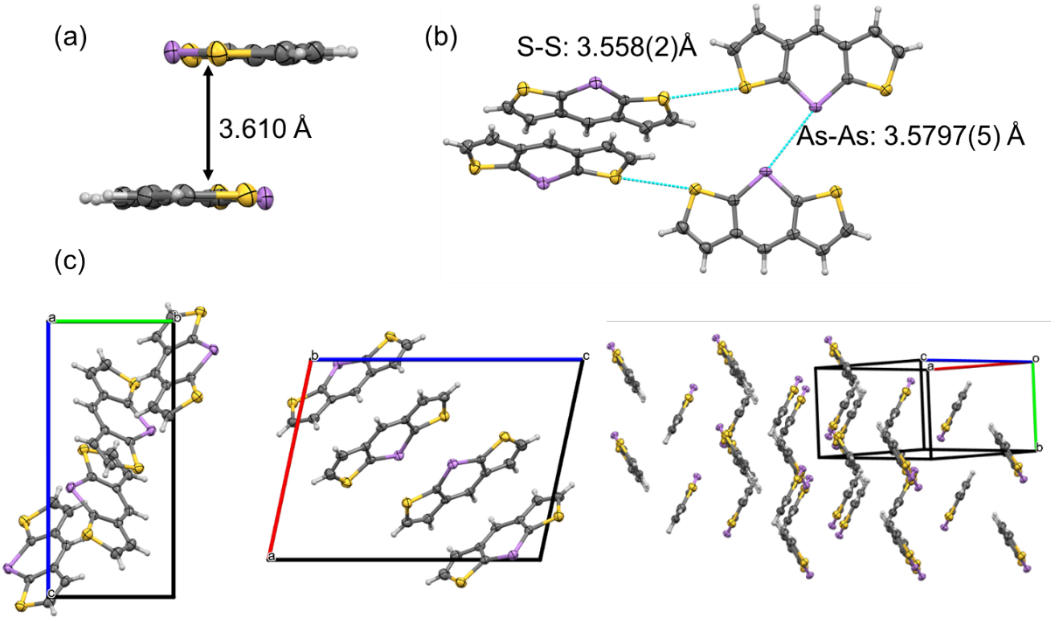

Figure S1. The crystal structure of **3a**. (a) stacking dimer. (b) Intermolecular interactions of As-As, S-S. (c) Packing structures along with a, b, c-axis.

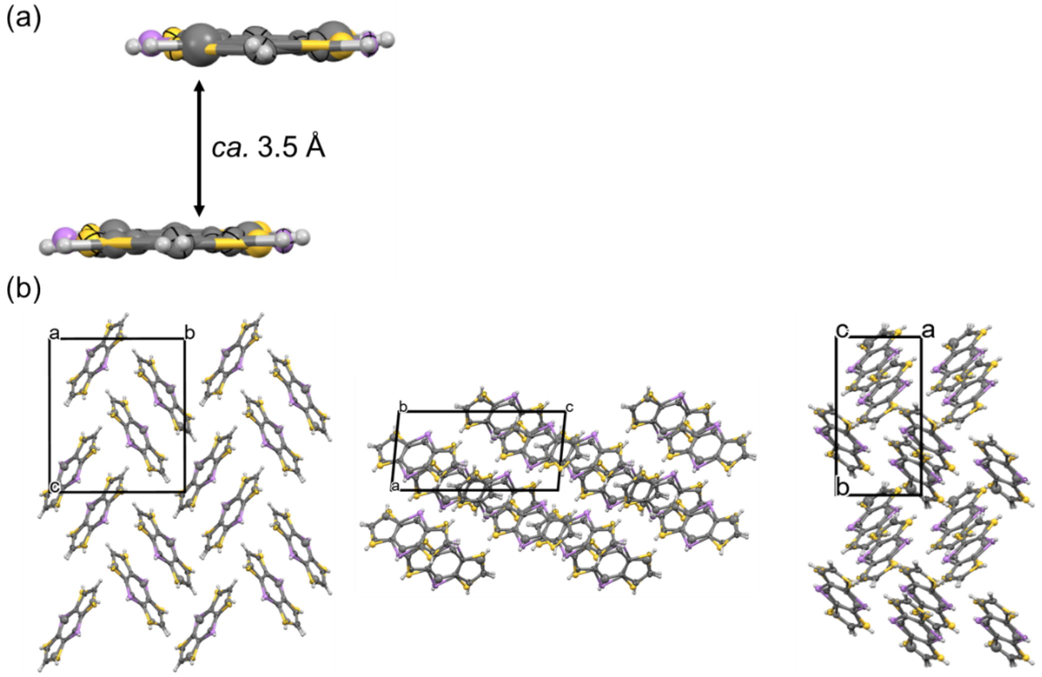

Figure S2. The crystal structure of **3b**. (a) stacking dimer. (b) Packing structures along with a, b, c-axis.

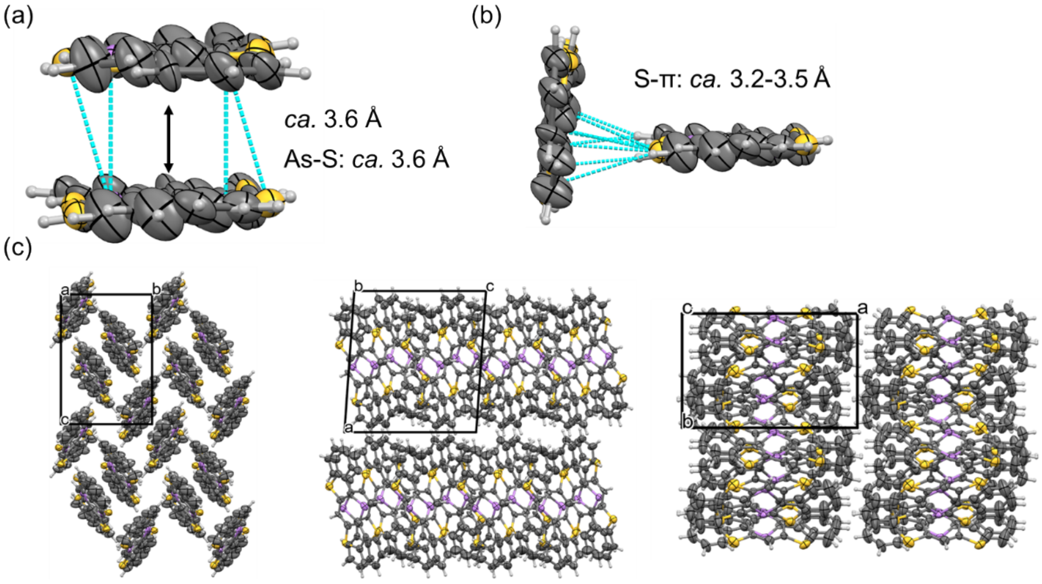

Figure S3. The crystal structure of **3d-plate**. (a) stacking dimer. (b) Intermolecular interactions of S-π. (c) Packing structures along with a, b, c-axis.

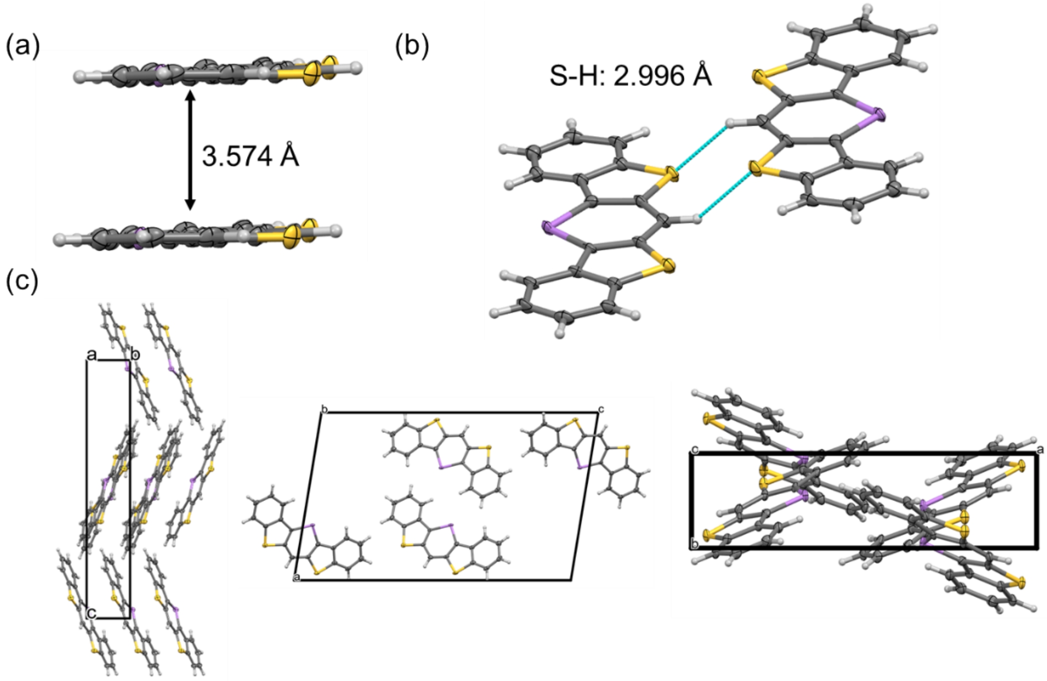

Figure S4. The crystal structure of **3d-needle**. (a) stacking dimer. (b) Intermolecular interactions of S-H. (c) Packing structures along with a, b, c-axis.

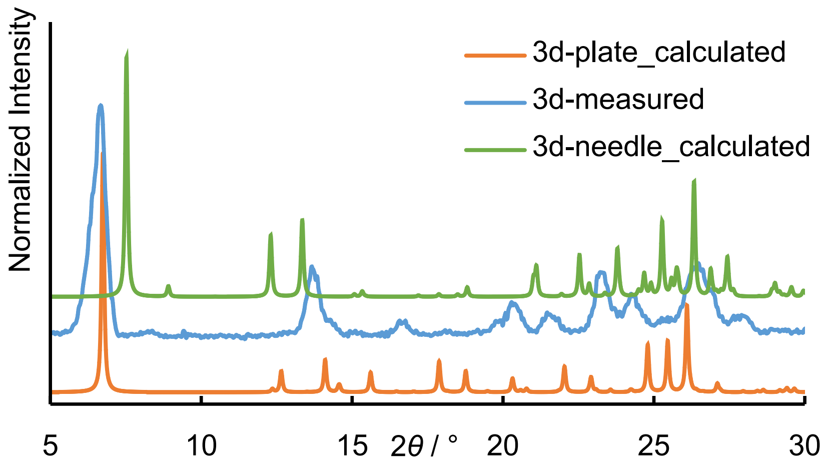

Figure S5. Powder XRD of **3d** used for photophysical and TRMC measurements.

5. Photophysical properties

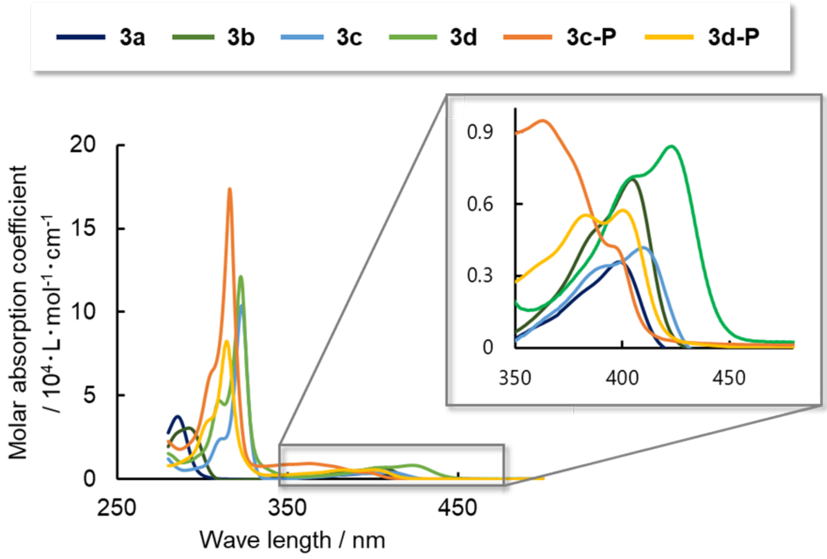

Figure S6. UV-vis spectra of **3a-d**, **3c-P**, and **3d-P** in 2-MeTHF.

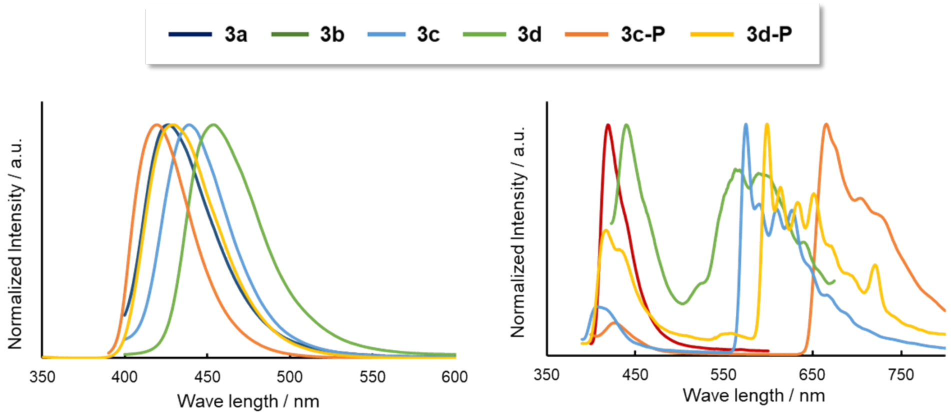

Figure S7. Emission spectra of **3a-d**, **3c-P**, and **3d-P** in 2-MeTHF at (a) 298 K and (b) 77 K.

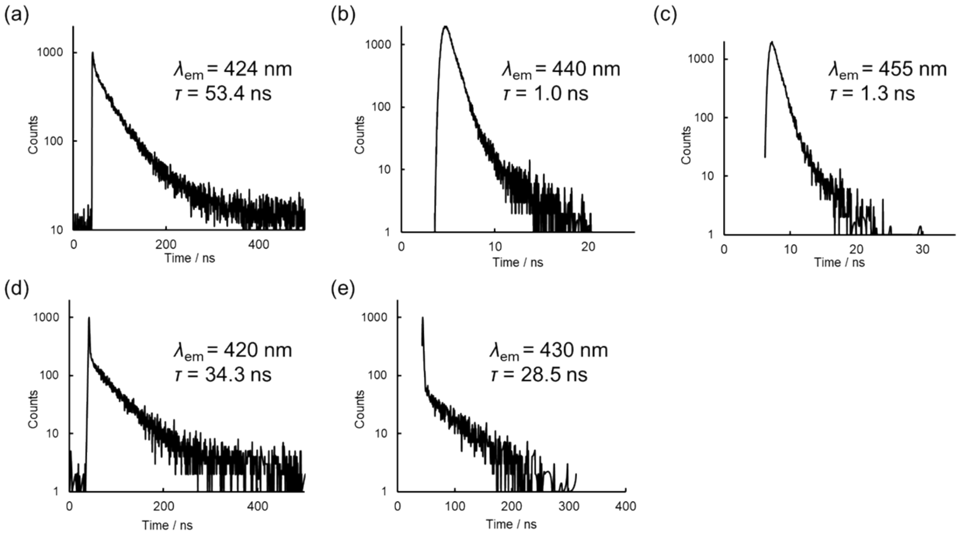

Figure S8. The emission decay kinetics in 2-MeTHF solution at 298 K. (a) **3a**, (b)**3c**, (c) **3d**, (d) **3c-P**, (e) **3d-P**.

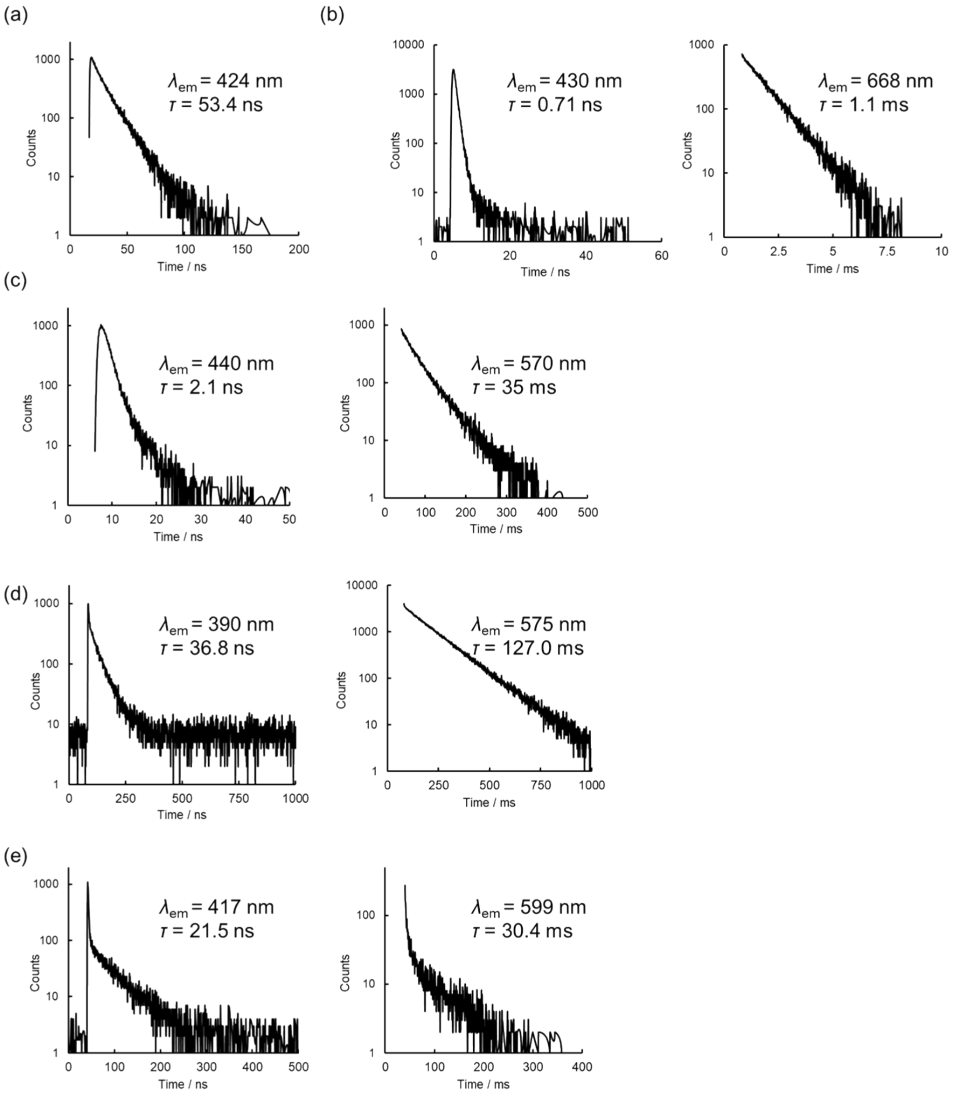

Figure S9. The emission decay kinetics in 2-MeTHF solution at 77K(a) **3a**, (b)**3c**, (c) **3d**, (d) **3c-P**, (e) **3d-P**.

Table S17. Photophysical properties of **3d-P** in 2-MeTHF solutions.

				298 K		77 K	
	λ_{abs} [nm]	ε [$M^{-1}\cdot cm^{-1}$]	λ_{ex} [nm]	λ_{em} [nm]	Φ	λ_{em} [nm]	Φ
**3d-P**	392	5700	316	430	0.04	417, 599	0.08, 0.20

6. Reactivity

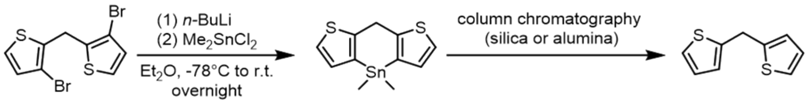

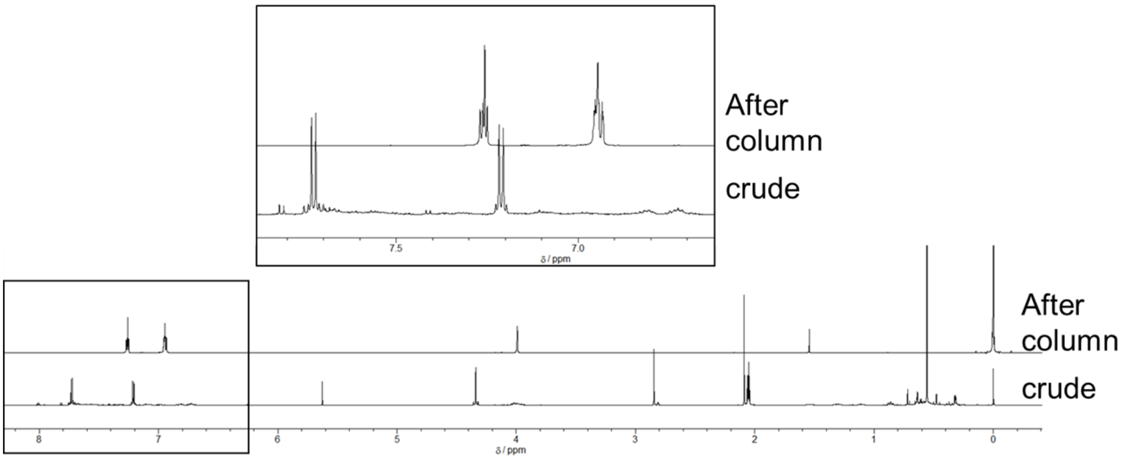

Figure S10. ^{1}H-NMR spectra of crude and after column chromatography of **2b**.

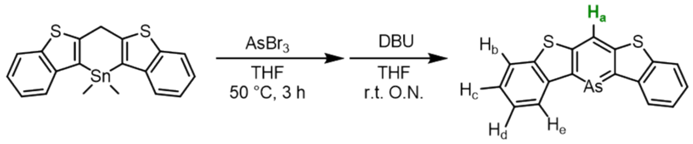

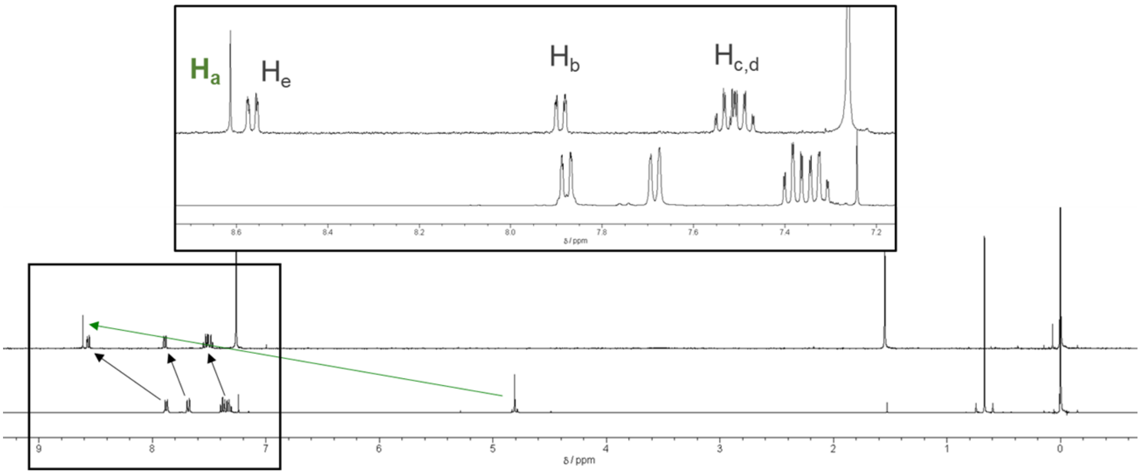

Figure S11. ^{1}H-NMR spectra of **2d** and **3d**. Methyl signal of Sn was disappeared and benzylic proton (4.81 ppm) of **2c** was shifted to 8.58 ppm.

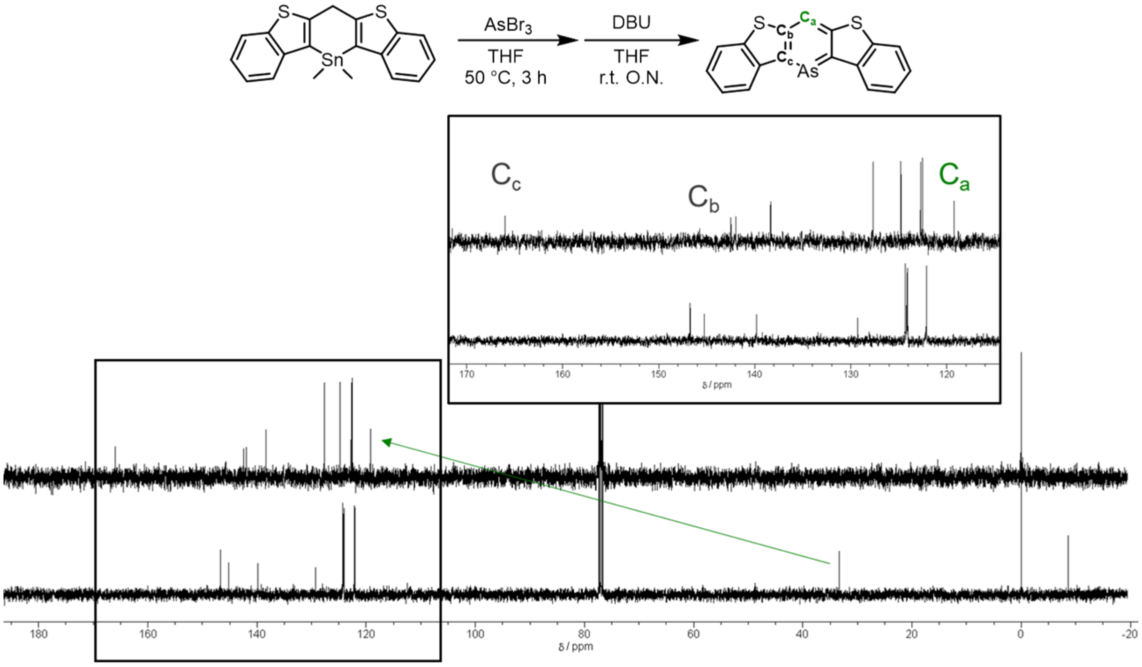

Figure S12. ^{13}C-NMR spectra of **2d** and **3d**. Methyl signal of Sn disappeared and benzylic carbon (33.3 ppm) of **2c** was shifted to 119.2 ppm.

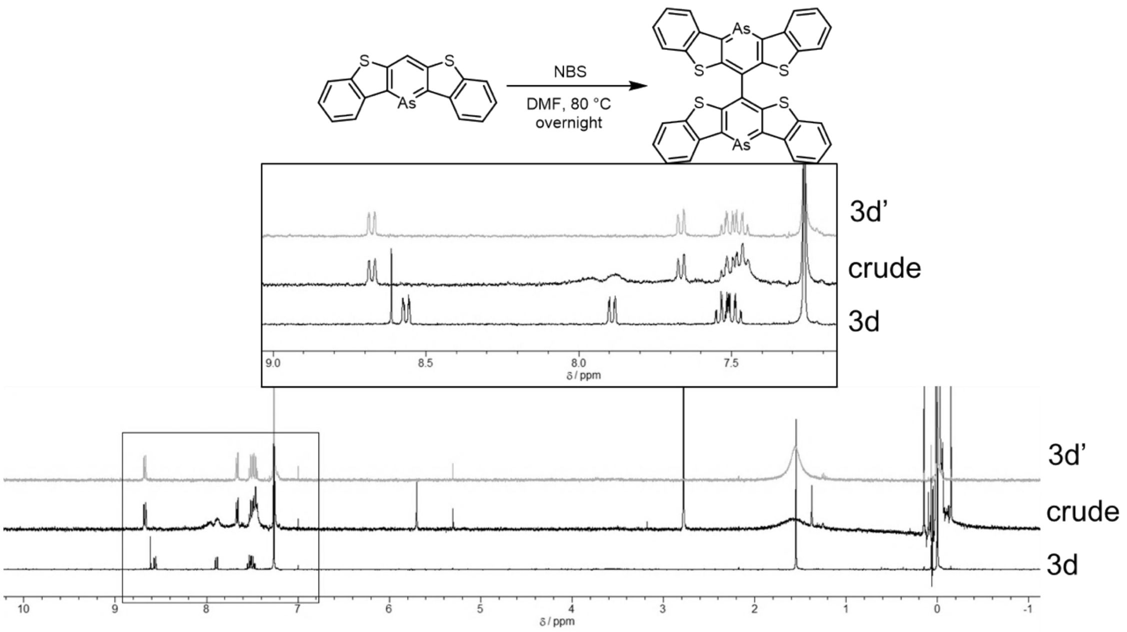

Figure S13. ^{1}H-NMR spectra of after the reaction of **3d** with NBS.

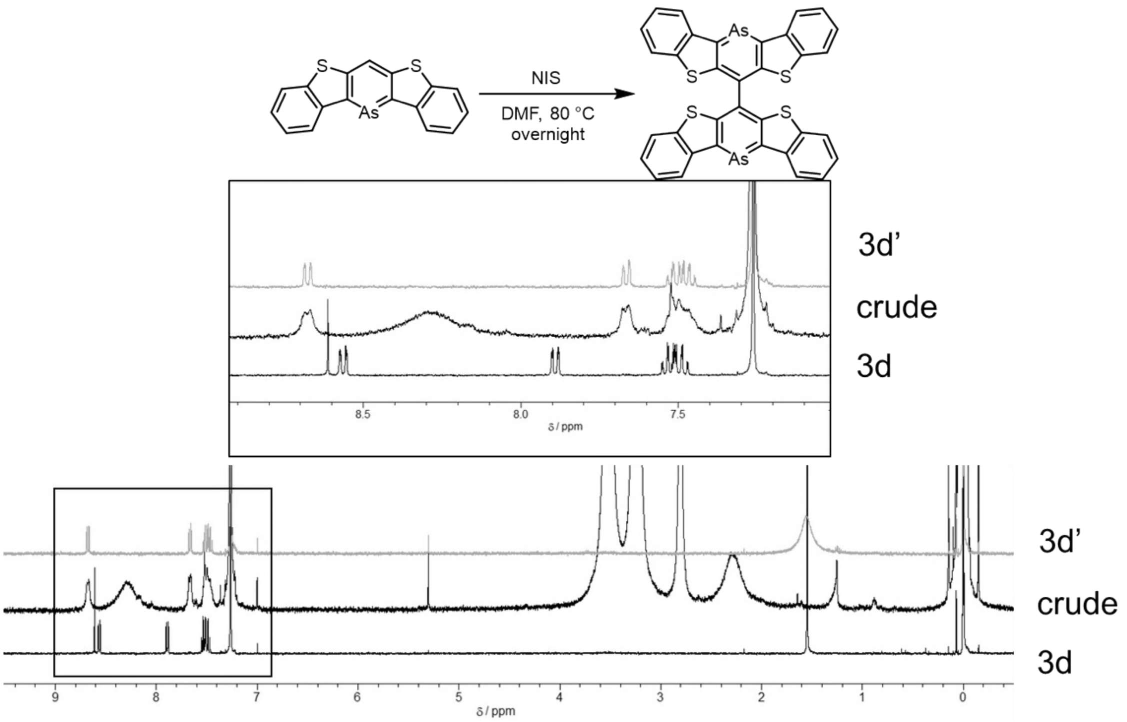

Figure S14. ^{1}H-NMR spectra of after the reaction of **3d** with NIS.

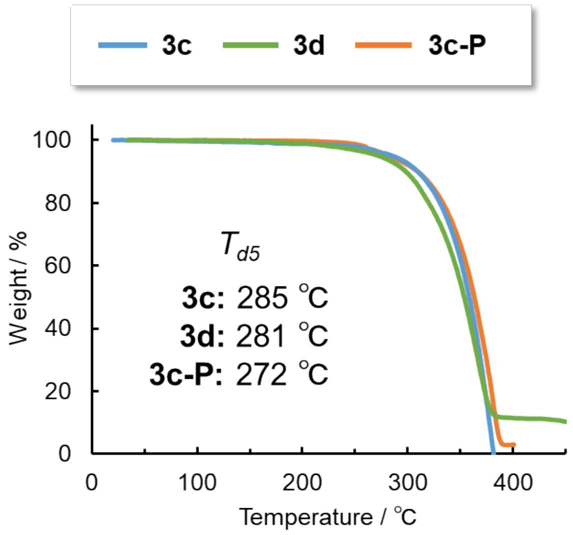

Figure S15. Thermal gravimetric analysis of **3c**, **3d** and **3c-P** under N_2.

7. Time-resolved microwave conductivity

Time-resolved microwave conductivity (TRMC) was measured for powder samples prepared on an adhesive tape on a quartz substrate. The microwave frequency and its power were ~9 GHz and ~3 mW, respectively. A third harmonic generation (355 nm) of a Nd:YAG laser (Continuum Inc., Surelite II, 5−8 ns pulse duration, 10 Hz) was used for the excitation (incident photon density I_0 = 9.1 × 10^{15} photons cm^{-2} $pulse^{-1}$). The photoconductivity ($\Delta\sigma$ = A^{-1} ΔP_r P_r^{-1} where A is the sensitivity factor, P_r is the reflected microwave power, and ΔP_r is the change in P_r upon exposure to light) was converted into the product of the quantum yield (φ) and sum of the charge carrier mobilities $\Sigma\mu$ (= μ_+ + μ_-) using the relationship $\varphi\Sigma\mu$ = $\Delta\sigma(eI_0F_{light})^{-1}$, where e and F_{Light} are the electron charge and correction (or filling) factor, respectively. The experiments were performed at room temperature in the air.

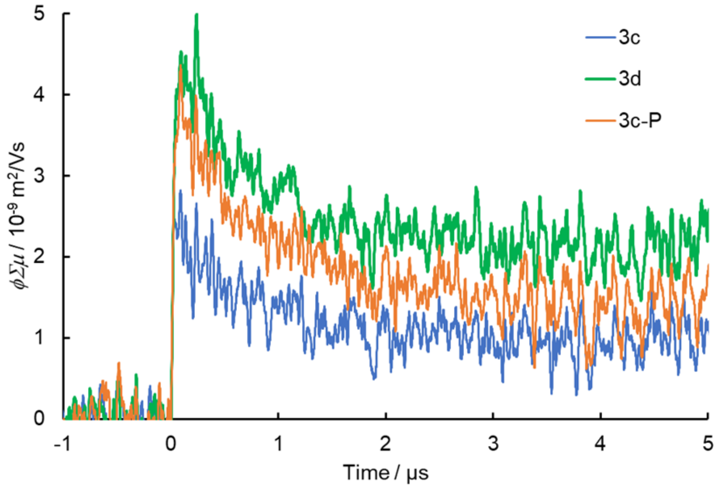

Figure S16. Photoconductivity transients recorded in crystalline powders of **3c-d** and **3c-P** upon excitation at 355 nm at 9.1 × 10^{15} photons cm^{-2} under air.

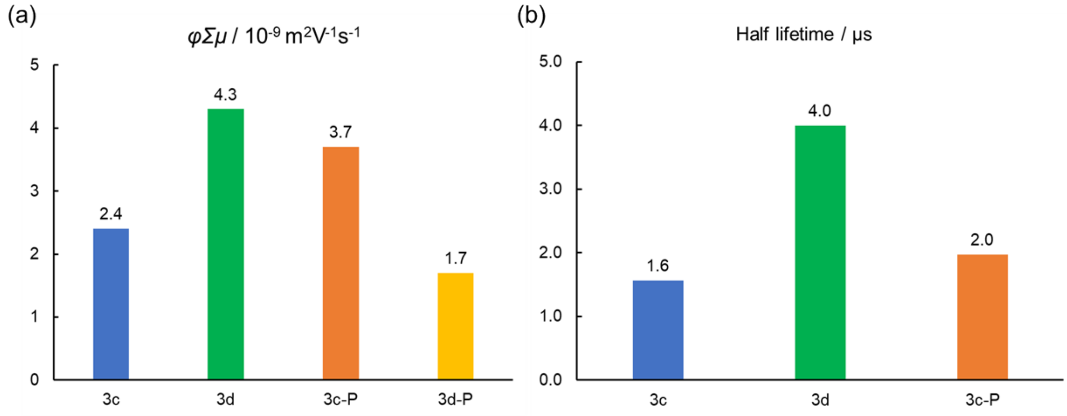

Figure S17. Summary of (a) photoconductivity of **3c**, **3c-d**, **3c-P**, and **3d-P**, and (b) life times of **3c**, **3c-d**, and **3c-P** (the signal of **3d-P** was too weak).

8. DFT calculations

Density functional theory (DFT) calculations were carried out to investigate the frontier orbitals. Initial structures were based on the X-ray data of the related compounds. For all the calculations, Gaussian 16 code was employed.[11] The calculations employed (TD-)B3LYP/def2-TZVP except for the **3a-W(CO)$_5$** and solid state dimer of **3c** and **3c-P**, which were calculated at B3LYP /def2-TZVP extended with Grimme's dispersion correction methods of D3 with Becke-Johnson damping (D3BJ) version and M06-2X/def2-TZVP, respectively. Also, natural bonding orbital (NBO) analysis was conducted by NBO program was version 7.0.[12] The nucleus-independent chemical shift NICS(1)$_{zz}$ were calculated at the geometrical center of the ring.[13] NICS-XY scans[14] were performed using the py.Aroma 3 package,[15] scanning from 1.7 Å above the plane of the ring. The anisotropy of the current (induced) density (ACID)[16] calculations were carried out at the CSGT-B3LYP/def2-TZVP level of theory. at the B3LYP-D3(BJ)/def2-TZVP level of theory. Moreover, non-covalent interaction (NCI) analysis[17] was performed using Multiwfn program packages,[18] which was represented by VMD1.9.3 program.[19] Charge transfer integrals in the crystal packing structures were calculated using the ADF2023 program[20] at the PBE/TZ2P level of theory.

Table S18. Hybrid of lone pair on Pn atom and Wiberg bond indices of C–Pn bond for pyridine, phosphinine, arsinine, **3a-d**, **3c-P**, and **3d-P** by NBO analysis based on the optimized structures.

	Hybrid orbital				WBI C–Pn	NPA charge
Pyridine	sp$^{2.25}$	s (30.7%)	p (69.1%)	d (0.2%)	1.44	-0.411
Phosphinine	sp$^{0.59}$	s (62.8%)	p (37.1%)	d (0.07%)	1.34	+0.712
Arsinine	sp$^{0.56}$	s (64.0%)	p (35.6%)	d (0.05%)	1.31	+0.765
**3a**	sp$^{0.38}$	s (72.2%)	p (27.7%)	d (0.03%)	1.22	+0.727
**3b**	sp$^{0.39}$	s (72.1%)	p (27.8%)	d (0.03%)	1.20	+0.701
**3c**	sp$^{0.38}$	s (72.3%)	p (27.7%)	d (0.03%)	1.22	+0.720
**3d**	sp$^{0.39}$	s (71.8%)	p (28.2%)	d (0.03%)	1.21	+0.715
**3c-P**	sp$^{0.61}$	s (62.2%)	p (37.7%)	d (0.03%)	1.27	+0.680
**3d-P**	sp$^{0.53}$	s (65.5%)	p (34.4%)	d (0.03%)	1.26	+0.679

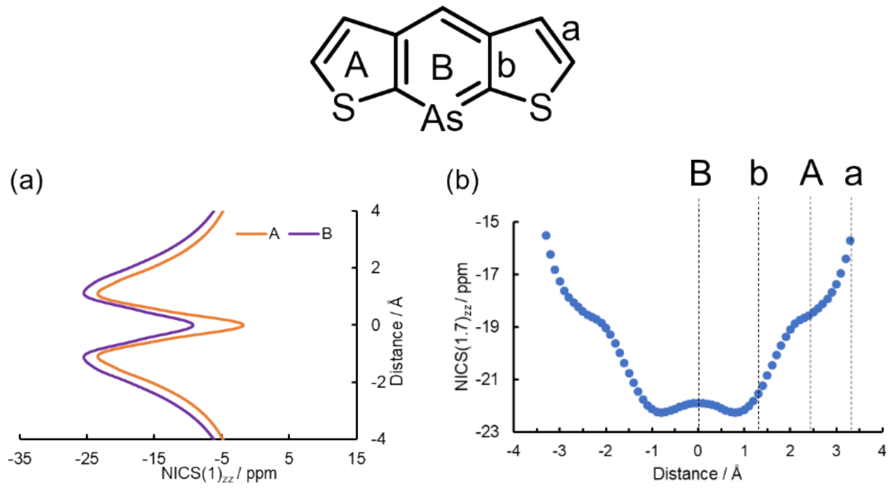

Figure S18. (a) NICS scan and (b) NICS-XY scan of **3a**

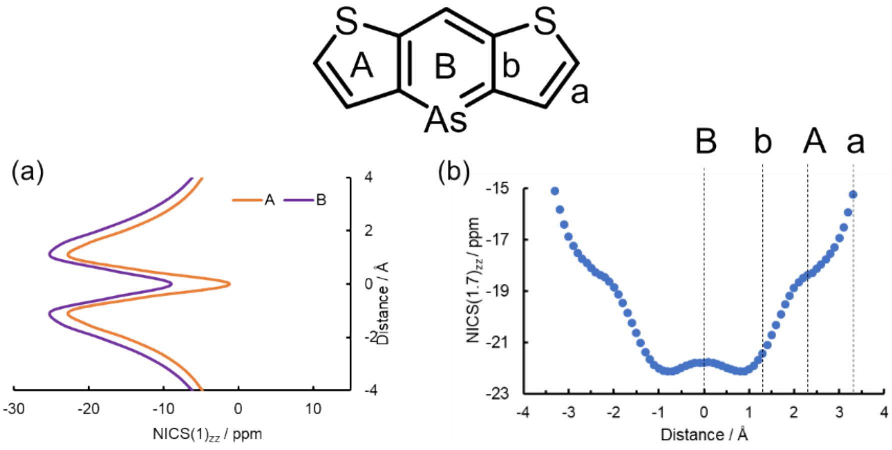

Figure S19. (a) NICS scan and (b) NICS-XY scan of **3b**

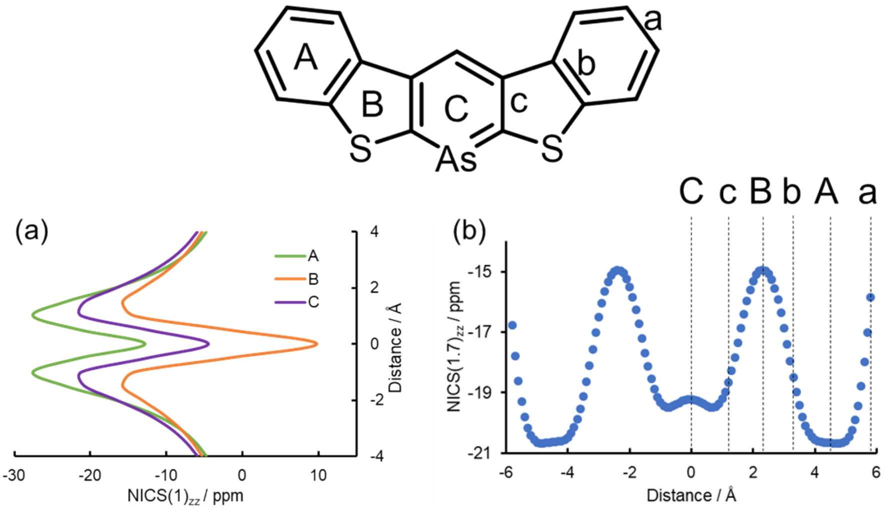

Figure S20. (a) NICS scan and (b) NICS-XY scan of **3c**

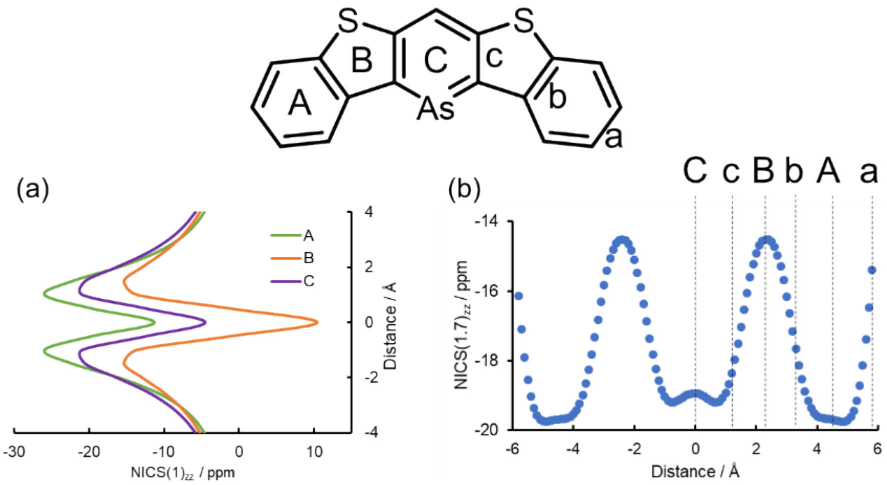

Figure S21. (a) NICS scan and (b) NICS-XY scan of **3d**

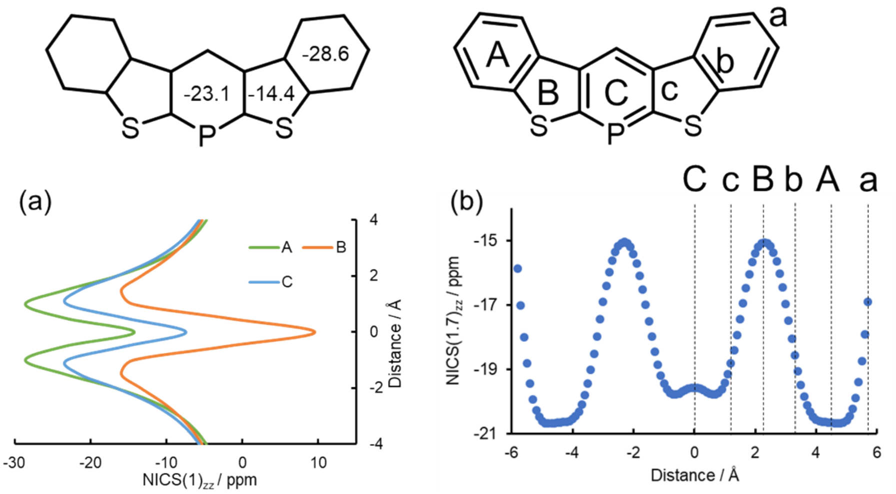

Figure S22. (a) NICS scan and (b) NICS-XY scan of **3c-P**

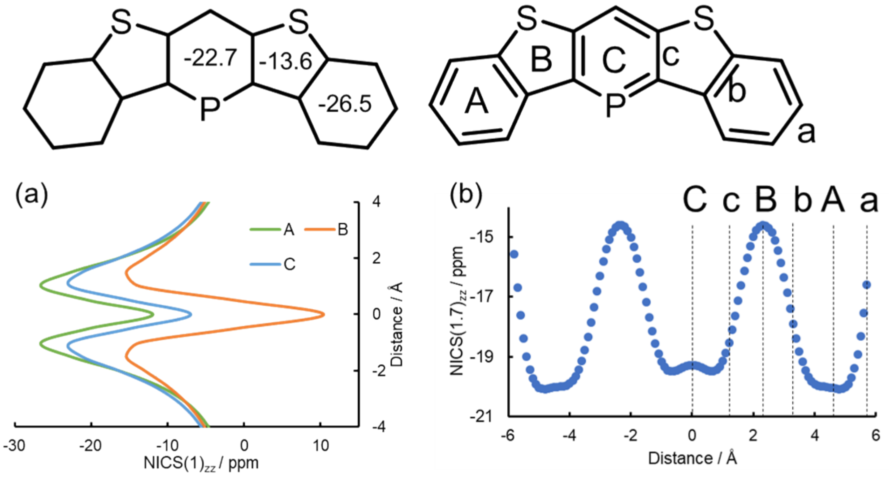

Figure S23. (a) NICS scan and (b) NICS-XY scan of **3d-P**

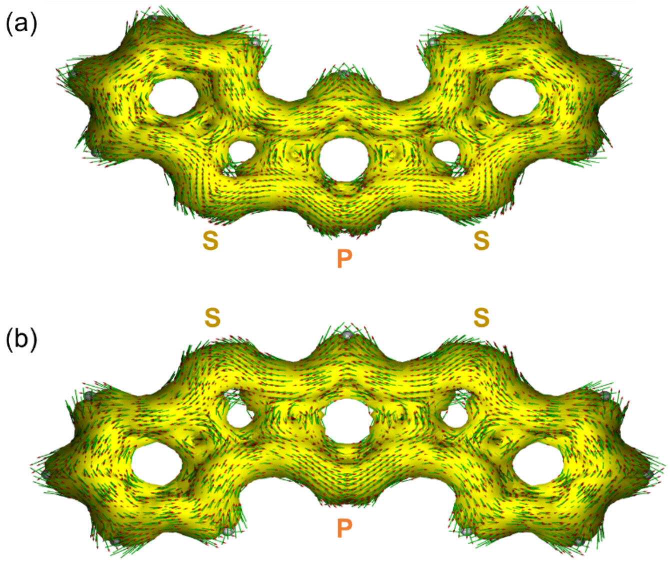

Figure S24. ACID plots of (a) **3c-P** and (b) **3d-P**.

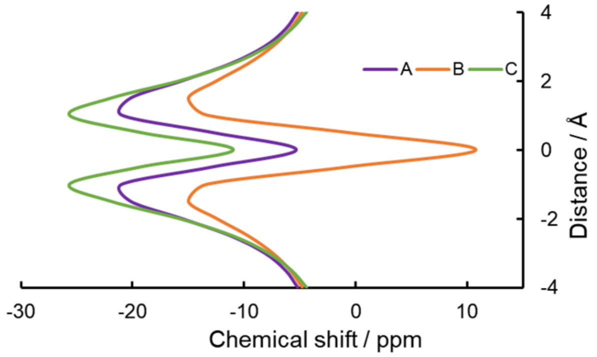

Figure S25. $NICS_{zz}$ scan of **3d'**. A: arsinine, B: thiophene, and C: benzene

Table S19. TD-DFT calculation for selected dimer d_1–d_4 of **3c** at M06/def2tzvp.

	HOMO / eV	LUMO / eV	ΔE / eV [a]	λ / nm [b]	f [c]
Monomer	−5.77	−2.00	3.09	401	0.0266
d_1	−5.60	−1.88	3.00	416	0.0126
d_2	−5.80	−2.07	3.09	402	0.0772
d_3	−5.65	−2.00	2.93	424	0.0214
d_4	−5.47	−2.17	2.61	476	0.2039
d_1–d_4	−5.44	−2.28	2.48	498	0.1479

[a] Excitation energy. [b] wavelength of the excitation [c] oscillator strength.

Table S20. TD-DFT calculation for selected dimer d_1-d_4 of **3c-P** at M06/def2tzvp.

	HOMO / eV	LUMO / eV	ΔE / eV [a]	λ / nm [b]	f [c]
Monomer	−6.85	−1.29	3.40	340	0.0215
d_4	−6.60	−1.40	3.64	365	0.1288

[a] Excitation energy. [b] wavelength of the excitation [c] oscillator strength.

Table S21. Transfer integrals between the dimer d_1-d_4 of **3c** at PBE/TZ2P**.**

	t_{HOMO} / meV	t_{LUMO} / meV	r_i /Å [a]
d_1	26.0	17.1	5.90
d_2	32.0	6.4	6.33
d_3	82.9	1.5	3.54
d_4	176.0	245.2	8.63

[a] Distances between the centroid of the molecules.

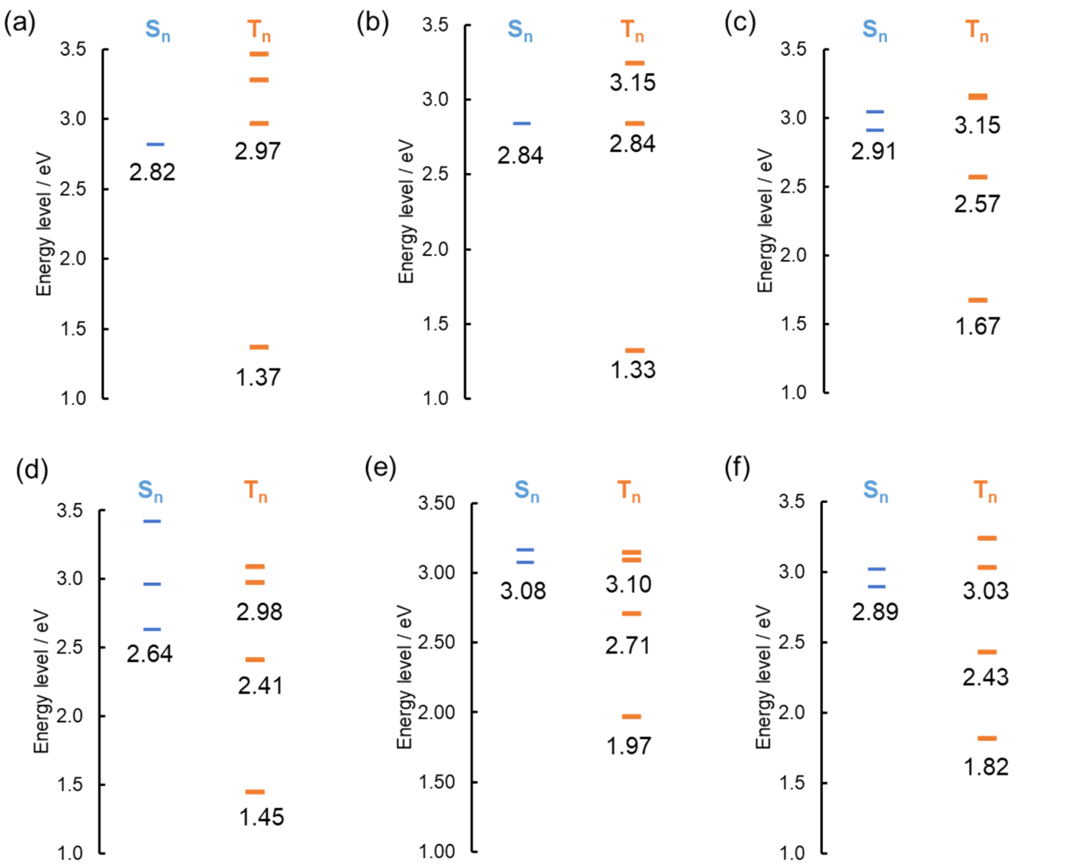

Figure S26. Energy levels of excited singlet and triplet state relative to the ground state of

(a) **3a**, (b) **3b**, (c) **3c**, (d) **3d**, (e) **3c-P**, and (f) **3d-P** based on the S_1 optimized geometries.

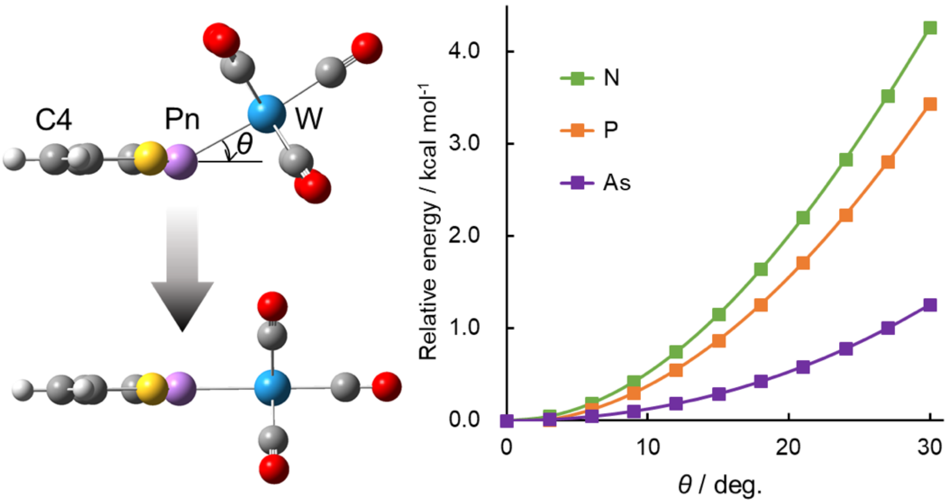

Figure S27. Energy plots of structures of nitrogen (green line), phosphorus (orange line) analogs of **3a-W(CO)₅** (purple line) with varying angles of W-Pn-C4. The energies were calculated based on the energies of the fully-optimized structures (B3LYP-D3BJ/def2TZVP).

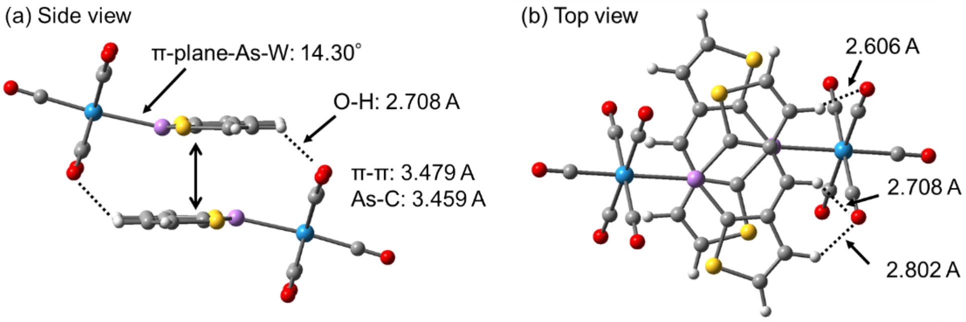

Figure S28. Optimized structure of stacking dimer of **3a-W(CO)₅** at B3LYP-D3BJ/def2TZVP. (a) Side view, (b) top view.

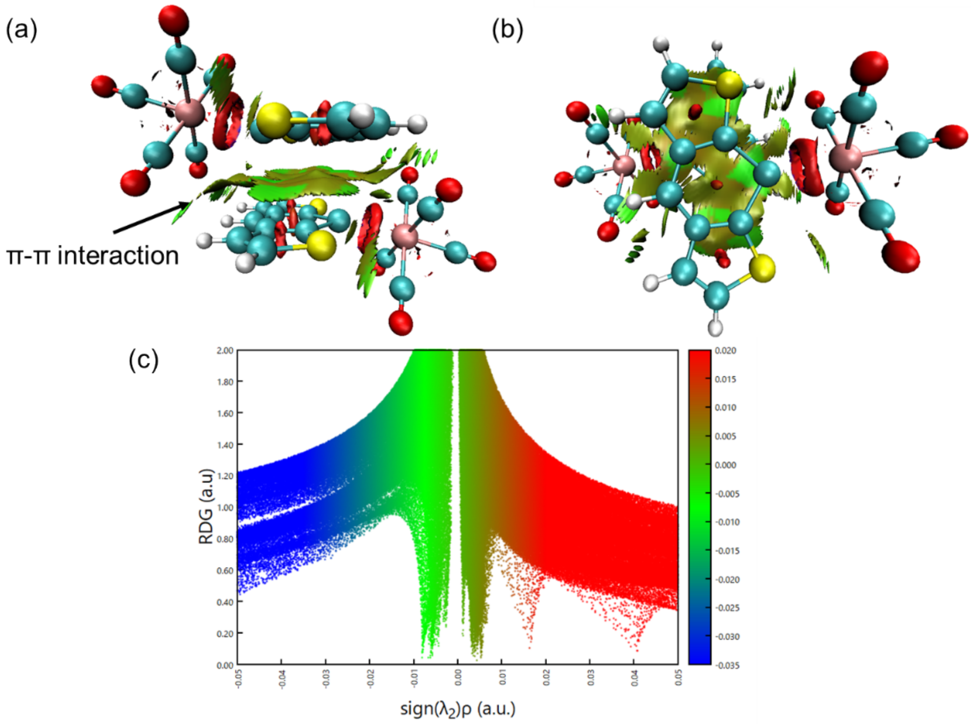

Figure S29. (a) (b) Noncovalent interaction (NCI) plots and (c) two-dimensional (2D) reduced density gradient (RDG) vs. sign(λ_2)ρ plots for stacking dimer of **3a-W(CO)$_5$**.

Cartesian coordinates in the structures optimized by DFT calculations

Table S22. **3a**: E(B3LYP/def2tzvp) = -3378.43954936 hartree

Number	Atom	X	Y	Z
1	C	3.558456	-1.016448	0.000010
2	C	1.375697	0.205892	0.000007
3	C	1.233097	-1.208014	-0.000003
4	As	0.000000	1.471643	0.000000
5	C	-1.375697	0.205892	-0.000007
6	C	-1.233097	-1.208014	0.000002
7	C	0.000000	-1.859450	0.000000
8	C	-3.558456	-1.016447	-0.000010
9	H	0.000000	-2.943670	0.000000
10	H	4.609734	-1.257635	0.000017
11	H	-4.609734	-1.257636	-0.000018
12	S	-3.059281	0.644449	0.000052
13	S	3.059281	0.644448	-0.000053

14	C	2.513966	-1.871111	0.000009
15	C	-2.513967	-1.871110	-0.000009
16	H	-2.619110	-2.947385	-0.000020
17	H	2.619107	-2.947386	0.000020

Table S23. **3b**: E(B3LYP/def2tzvp) = -3378.49280209 hartree

Number	Atom	X	Y	Z
1	C	-2.338602	1.757408	0.000000
2	C	-1.939829	3.053108	0.000000
3	C	-1.250988	0.825166	0.000000
4	C	0.000000	1.490774	0.000000
5	S	-0.219308	3.231754	0.000000
6	As	-1.448239	-1.036349	0.000000
7	C	0.377332	-1.450303	0.000000
8	C	1.411056	-0.481056	0.000000
9	C	1.254567	0.897760	0.000000
10	C	0.908681	-2.780589	0.000000
11	C	2.263739	-2.821308	0.000000
12	S	2.988085	-1.250484	0.000000
13	H	2.135035	1.527829	0.000000
14	H	-2.565287	3.931516	0.000000
15	H	-3.377071	1.457584	0.000000
16	H	0.289739	-3.666716	0.000000
17	H	2.893310	-3.696774	0.000000

Table S24. **3c**: E(B3LYP/def2tzvp) = -3685.84714488 hartree

Number	Atom	X	Y	Z
1	C	5.175215	-1.931572	0.000042
2	C	4.117460	-2.846685	0.000058
3	C	2.804590	-2.406677	0.000049
4	C	2.527999	-1.035358	0.000023
5	C	3.605069	-0.132769	0.000008
6	C	4.927972	-0.568136	0.000017
7	C	1.235670	-0.358135	0.000009
8	C	1.378944	1.048569	-0.000019
9	S	3.058230	1.528899	-0.000021

10	C	-1.235670	-0.358135	0.000005
11	C	-1.378944	1.048569	-0.000023
12	C	-2.527999	-1.035358	0.000016
13	C	-3.605069	-0.132769	-0.000003
14	S	-3.058230	1.528899	-0.000033
15	C	-2.804590	-2.406677	0.000042
16	C	-4.117460	-2.846685	0.000048
17	C	-5.175215	-1.931572	0.000029
18	C	-4.927972	-0.568136	0.000003
19	H	6.196707	-2.289712	0.000050
20	H	4.327975	-3.908318	0.000078
21	H	1.996637	-3.127283	0.000061
22	H	5.745006	0.141658	0.000006
23	H	-1.996637	-3.127283	0.000057
24	H	-4.327975	-3.908318	0.000068
25	H	-6.196707	-2.289712	0.000034
26	H	-5.745006	0.141658	-0.000011
27	C	0.000000	-1.001045	0.000020
28	As	0.000000	2.312173	-0.000045
29	H	0.000000	-2.083733	0.000041

Table S25. **3d**: E(B3LYP/def2tzvp) = -3685.84854308 hartree

Number	Atom	X	Y	Z
1	C	-0.726750	5.597049	0.000000
2	C	-1.862988	4.780139	0.000000
3	C	-1.740056	3.402633	0.000000
4	C	-0.469140	2.812691	0.000000
5	C	0.662015	3.653911	0.000000
6	C	0.542453	5.041112	0.000000
7	C	-0.119437	1.406672	0.000000
8	C	1.276108	1.216066	0.000000
9	S	2.155009	2.746208	0.000000
10	As	-1.352704	-0.000001	0.000000
11	C	-0.119435	-1.406670	0.000000
12	C	1.276111	-1.216069	0.000000
13	C	1.946901	0.000000	0.000000

14	C	-0.469139	-2.812690	0.000000
15	C	0.662013	-3.653911	0.000000
16	S	2.155009	-2.746209	0.000000
17	C	-1.740056	-3.402632	0.000000
18	C	-1.862988	-4.780137	0.000000
19	C	-0.726750	-5.597049	0.000000
20	C	0.542453	-5.041113	0.000000
21	H	-0.838193	6.673708	0.000000
22	H	-2.846705	5.231287	0.000000
23	H	-2.625674	2.779274	0.000000
24	H	1.421176	5.672789	0.000000
25	H	3.029843	0.000002	0.000000
26	H	-2.625674	-2.779273	0.000000
27	H	-2.846704	-5.231287	0.000000
28	H	-0.838196	-6.673708	0.000000
29	H	1.421177	-5.672787	0.000000

Table S26. **3c-P**: E(B3LYP/def2tzvp) = -1791.31651287 hartree

Number	Atom	X	Y	Z
1	C	5.209304	-1.582418	0.000036
2	C	4.182956	-2.533043	0.000051
3	C	2.856393	-2.137176	0.000042
4	C	2.535407	-0.775535	0.000017
5	C	3.580938	0.164336	0.000003
6	C	4.917426	-0.227858	0.000013
7	C	1.224545	-0.143165	0.000003
8	C	1.316572	1.270569	-0.000029
9	S	2.985015	1.808668	-0.000022
10	C	-1.224545	-0.143165	0.000000
11	C	-1.316572	1.270569	-0.000029
12	C	-2.535407	-0.775535	0.000011
13	C	-3.580938	0.164336	-0.000008
14	S	-2.985015	1.808668	-0.000037
15	C	-2.856393	-2.137176	0.000037
16	C	-4.182956	-2.533043	0.000043
17	C	-5.209304	-1.582418	0.000023

18	C	-4.917426	-0.227858	-0.000003
19	H	6.242229	-1.906131	0.000044
20	H	4.429064	-3.586963	0.000070
21	H	2.071801	-2.883285	0.000054
22	H	5.711056	0.507983	0.000003
23	H	-2.071801	-2.883285	0.000053
24	H	-4.429064	-3.586963	0.000063
25	H	-6.242229	-1.906131	0.000028
26	H	-5.711056	0.507983	-0.000017
27	C	0.000000	-0.803955	0.000016
28	H	0.000000	-1.886688	0.000038
29	P	0.000000	2.409421	-0.000049

Table S27. **3a-W(CO)$_5$ stacking dimer**: E(B3LYP/def2tzvp) = -8025.42048944 hartree

Number	Atom	X	Y	Z
1	W	-4.531821	0.191050	0.046994
2	S	-1.016637	2.203699	-1.866735
3	S	-1.997213	-3.593465	-0.156147
4	O	-3.578854	2.912055	1.445967
5	O	-5.562891	-2.519237	-1.341634
6	O	-4.758618	1.739163	-2.761007
7	O	-4.399245	-1.338223	2.860811
8	C	-4.667785	1.187966	-1.765947
9	O	-7.535354	0.977892	0.660280
10	C	-4.426323	-0.798632	1.851878
11	C	-3.898762	1.935388	0.940758
12	C	-5.188901	-1.559529	-0.850479
13	C	-0.910876	0.492065	-1.625742
14	C	-0.135950	-2.383988	-1.534544
15	C	0.597713	-1.401030	-2.196551
16	H	1.504729	-1.704619	-2.701793
17	C	0.222151	-3.778383	-1.462625
18	H	1.121033	-4.174643	-1.911306
19	C	-6.446270	0.693333	0.442255
20	C	0.245901	-0.052110	-2.245332
21	C	0.454945	2.207945	-2.783506

22	H	0.830666	3.138080	-3.177106
23	C	1.003016	0.979850	-2.908041
24	H	1.919370	0.786948	-3.444415
25	C	-1.352659	-2.138896	-0.840826
26	C	-0.665588	-4.523292	-0.769802
27	H	-0.627454	-5.583409	-0.577219
28	As	-2.109969	-0.476289	-0.620256
29	W	4.531717	-0.191235	-0.047077
30	S	1.015787	-2.203420	1.867431
31	S	1.998113	3.593192	0.155984
32	O	3.579174	-2.912222	-1.446364
33	O	5.563331	2.518755	1.341736
34	O	4.759148	-1.740188	2.760416
35	O	4.398228	1.338660	-2.860513
36	C	4.668190	-1.188673	1.765543
37	O	7.535094	-0.977716	-0.661609
38	C	4.425664	0.798835	-1.851718
39	C	3.898804	-1.935536	-0.941026
40	C	5.189113	1.559161	0.850537
41	C	0.910544	-0.491774	1.626201
42	C	0.136355	2.384467	1.534378
43	C	-0.597681	1.401804	2.196414
44	H	-1.504763	1.705710	2.701341
45	C	-0.221371	3.778939	1.462151
46	H	-1.120216	4.175518	1.910625
47	C	6.446070	-0.693292	-0.443113
48	C	-0.246209	0.052810	2.245479
49	C	-0.455965	-2.207124	2.783932
50	H	-0.832020	-3.137098	3.177594
51	C	-1.003749	-0.978870	2.908158
52	H	-1.920153	-0.785646	3.444329
53	C	1.353096	2.138925	0.840886
54	C	0.666660	4.523484	0.769309
55	H	0.628817	5.583573	0.576514
56	As	2.110151	0.476126	0.620940

Table S28. **3d_P** (S_0): E(B3LYP/def2tzvp) = -1791.31773813 hartree

Number	Atom	X	Y	Z
1	C	-0.974413	5.514673	0.000000
2	C	-2.083414	4.661417	0.000000
3	C	-1.916198	3.288009	0.000000
4	C	-0.626902	2.741466	0.000000
5	C	0.476382	3.617449	0.000000
6	C	0.312335	5.000209	0.000000
7	C	-0.230627	1.343414	0.000000
8	C	1.176138	1.205886	0.000000
9	S	1.999990	2.759061	0.000000
10	C	-0.230627	-1.343414	0.000000
11	C	1.176138	-1.205886	0.000000
12	C	1.862641	0.000000	0.000000
13	C	-0.626902	-2.741466	0.000000
14	C	0.476382	-3.617449	0.000000
15	S	1.999989	-2.759061	0.000000
16	C	-1.916199	-3.288009	0.000000
17	C	-2.083415	-4.661417	0.000000
18	C	-0.974413	-5.514672	0.000000
19	C	0.312335	-5.000209	0.000000
20	H	-1.120535	6.587229	0.000000
21	H	-3.081310	5.080429	0.000000
22	H	-2.779425	2.634235	0.000000
23	H	1.170297	5.659954	0.000000
24	H	2.945323	0.000000	0.000000
25	H	-2.779425	-2.634234	0.000000
26	H	-3.081310	-5.080429	0.000000
27	H	-1.120535	-6.587229	0.000000
28	H	1.170296	-5.659954	0.000000
29	P	-1.339899	0.000000	0.000000

Table S29. **3d_P_S1** (S_1): E(B3LYP/def2tzvp) = -1791.31279633 hartree

Number	Atom	X	Y	Z
1	C	0.000000	5.502148	0.996038
2	C	0.000000	4.643313	2.087660

3	C	0.000000	3.261208	1.908533
4	C	0.000000	2.721699	0.610150
5	C	0.000000	3.619050	-0.483016
6	C	0.000000	4.987860	-0.311989
7	C	0.000000	1.344500	0.219167
8	C	0.000000	1.201993	-1.196921
9	S	0.000000	2.746765	-2.004891
10	C	0.000000	-1.344500	0.219167
11	C	0.000000	-1.201993	-1.196921
12	C	0.000000	0.000000	-1.909166
13	C	0.000000	-2.721699	0.610150
14	C	0.000000	-3.619050	-0.483016
15	S	0.000000	-2.746765	-2.004891
16	C	0.000000	-3.261208	1.908533
17	C	0.000000	-4.643313	2.087660
18	C	0.000000	-5.502148	0.996038
19	C	0.000000	-4.987860	-0.311989
20	H	0.000000	6.573629	1.145970
21	H	0.000000	5.050283	3.090552
22	H	0.000000	2.598695	2.764473
23	H	0.000000	5.657635	-1.162226
24	H	0.000000	0.000000	-2.989519
25	H	0.000000	-2.598695	2.764473
26	H	0.000000	-5.050283	3.090552
27	H	0.000000	-6.573629	1.145970
28	H	0.000000	-5.657635	-1.162226
29	P	0.000000	0.000000	1.397867

Table S30. **3a_S1** (S_1): E(B3LYP/def2tzvp) = -3378.43434935 hartree

Number	Atom	X	Y	Z
1	C	3.581079	-1.000340	0.000015
2	C	1.374417	0.173561	-0.000005
3	C	1.236431	-1.222562	-0.000007
4	As	0.000000	1.512117	0.000000
5	C	-1.374417	0.173561	0.000005
6	C	-1.236431	-1.222562	0.000007

7	C	0.000000	-1.907192	0.000000
8	C	-3.581079	-1.000340	-0.000014
9	H	0.000000	-2.989089	0.000000
10	H	4.632075	-1.239012	0.000034
11	H	-4.632075	-1.239012	-0.000032
12	S	-3.047448	0.621725	0.000052
13	S	3.047448	0.621725	-0.000052
14	C	2.518803	-1.867245	-0.000005
15	C	-2.518803	-1.867245	0.000005
16	H	-2.637000	-2.942879	-0.000007
17	H	2.637000	-2.942879	0.000007

Table S31. **3c-P_S1** (S_1): E(B3LYP/None) = -1791.31817186 hartree

Number	Atom	X	Y	Z
1	C	5.211685	-1.588841	0.000035
2	C	4.190374	-2.534075	0.000053
3	C	2.854516	-2.138744	0.000045
4	C	2.530904	-0.773641	0.000018
5	C	3.584667	0.163694	0.000001
6	C	4.912761	-0.219002	0.000009
7	C	1.225665	-0.158467	0.000005
8	C	1.316738	1.265446	-0.000021
9	S	2.963925	1.802091	-0.000029
10	C	-1.225665	-0.158467	0.000002
11	C	-1.316738	1.265446	-0.000025
12	C	-2.530904	-0.773641	0.000011
13	C	-3.584667	0.163694	-0.000008
14	S	-2.963925	1.802091	-0.000038
15	C	-2.854516	-2.138744	0.000037
16	C	-4.190374	-2.534075	0.000041
17	C	-5.211685	-1.588841	0.000022
18	C	-4.912761	-0.219002	-0.000003
19	H	6.245717	-1.906877	0.000042
20	H	4.434544	-3.588219	0.000073
21	H	2.071999	-2.885901	0.000059
22	H	5.706952	0.515674	-0.000004

23	H	-2.071999	-2.885901	0.000053
24	H	-4.434544	-3.588219	0.000061
25	H	-6.245717	-1.906877	0.000026
26	H	-5.706952	0.515674	-0.000019
27	C	0.000000	-0.854473	0.000016
28	H	0.000000	-1.933507	0.000035
29	P	0.000000	2.461842	-0.000045

Table S32. **3c_S1** (S_0): E(B3LYP/def2tzvp) = -3685.84390720 hartree

Number	Atom	X	Y	Z
1	C	5.179163	-1.940232	0.000044
2	C	4.128404	-2.851768	0.000059
3	C	2.806649	-2.413407	0.000049
4	C	2.523172	-1.040591	0.000023
5	C	3.606265	-0.136997	0.000008
6	C	4.921917	-0.563782	0.000018
7	C	1.235638	-0.382315	0.000008
8	C	1.373995	1.036254	-0.000018
9	S	3.036974	1.517947	-0.000024
10	C	-1.235638	-0.382315	0.000005
11	C	-1.373995	1.036254	-0.000021
12	C	-2.523172	-1.040591	0.000016
13	C	-3.606265	-0.136997	-0.000002
14	S	-3.036974	1.517947	-0.000032
15	C	-2.806649	-2.413407	0.000041
16	C	-4.128404	-2.851768	0.000048
17	C	-5.179163	-1.940232	0.000030
18	C	-4.921917	-0.563782	0.000005
19	H	6.202675	-2.291289	0.000052
20	H	4.338662	-3.913432	0.000080
21	H	2.001269	-3.135898	0.000062
22	H	5.738245	0.146833	0.000006
23	H	-2.001269	-3.135898	0.000055
24	H	-4.338662	-3.913432	0.000067
25	H	-6.202675	-2.291289	0.000035
26	H	-5.738245	0.146833	-0.000009

27	C	0.000000	-1.057620	0.000019
28	As	0.000000	2.357885	-0.000045
29	H	0.000000	-2.137183	0.000038

Table S33. **3d'** (S_0): E(B3LYP/def2tzvp) = -7370.48872066 hartree

Number	Atom	X	Y	Z
1	C	0.000000	0.000000	0.748125
2	C	0.000000	1.217370	1.438304
3	C	0.000000	-1.217370	1.438304
4	C	0.000000	1.401607	2.835631
5	S	0.000000	2.754090	0.569997
6	C	0.000000	0.000000	-0.748125
7	C	0.000000	-1.401607	2.835631
8	S	0.000000	-2.754090	0.569997
9	C	0.000000	2.805195	3.194288
10	As	0.000000	0.000000	4.069387
11	C	0.000000	3.651019	2.068205
12	C	1.217370	0.000000	-1.438304
13	C	-1.217370	0.000000	-1.438304
14	C	0.000000	-2.805195	3.194288
15	C	0.000000	-3.651019	2.068205
16	C	0.000000	3.387004	4.469065
17	C	0.000000	5.037853	2.195067
18	C	1.401607	0.000000	-2.835631
19	S	2.754090	0.000000	-0.569997
20	C	-1.401607	0.000000	-2.835631
21	S	-2.754090	0.000000	-0.569997
22	C	0.000000	-3.387004	4.469065
23	C	0.000000	-5.037853	2.195067
24	C	0.000000	4.763864	4.599357
25	H	0.000000	2.758911	5.351364
26	C	0.000000	5.586822	3.467257
27	H	0.000000	5.674019	1.319577
28	C	2.805195	0.000000	-3.194288
29	As	0.000000	0.000000	-4.069387
30	C	3.651019	0.000000	-2.068205

31	C	-2.805195	0.000000	-3.194288
32	C	-3.651019	0.000000	-2.068205
33	C	0.000000	-4.763864	4.599357
34	H	0.000000	-2.758911	5.351364
35	C	0.000000	-5.586822	3.467257
36	H	0.000000	-5.674019	1.319577
37	H	0.000000	5.209801	5.585470
38	H	0.000000	6.662892	3.584577
39	C	3.387004	0.000000	-4.469065
40	C	5.037853	0.000000	-2.195067
41	C	-3.387004	0.000000	-4.469065
42	C	-5.037853	0.000000	-2.195067
43	H	0.000000	-5.209801	5.585470
44	H	0.000000	-6.662892	3.584577
45	C	4.763864	0.000000	-4.599357
46	H	2.758911	0.000000	-5.351364
47	C	5.586822	0.000000	-3.467257
48	H	5.674019	0.000000	-1.319577
49	C	-4.763864	0.000000	-4.599357
50	H	-2.758911	0.000000	-5.351364
51	C	-5.586822	0.000000	-3.467257
52	H	-5.674019	0.000000	-1.319577
53	H	5.209801	0.000000	-5.585470
54	H	6.662892	0.000000	-3.584577
55	H	-5.209801	0.000000	-5.585470
56	H	-6.662892	0.000000	-3.584577

Table S34. **3d_S1** (S_1): E(B3LYP/def2tzvp) = -3685.84451172 hartree

Number	Atom	X	Y	Z
1	C	-0.690527	5.618295	0.000000
2	C	-1.828105	4.796634	0.000000
3	C	-1.724046	3.414995	0.000000
4	C	-0.456220	2.811862	0.000000
5	C	0.684491	3.668262	0.000000
6	C	0.577193	5.057201	0.000000
7	C	-0.113340	1.420233	0.000000

8	C	1.264029	1.214463	0.000000
9	S	2.141824	2.743045	0.000000
10	As	-1.405785	0.000000	0.000000
11	C	-0.113340	-1.420233	0.000000
12	C	1.264029	-1.214464	0.000000
13	C	1.962940	0.000000	0.000000
14	C	-0.456220	-2.811862	0.000000
15	C	0.684491	-3.668262	0.000000
16	S	2.141824	-2.743045	0.000000
17	C	-1.724046	-3.414995	0.000000
18	C	-1.828105	-4.796634	0.000000
19	C	-0.690527	-5.618295	0.000000
20	C	0.577193	-5.057201	0.000000
21	H	-0.803335	6.694201	0.000000
22	H	-2.809258	5.254632	0.000000
23	H	-2.614184	2.799255	0.000000
24	H	1.461345	5.681181	0.000000
25	H	3.044083	0.000000	0.000000
26	H	-2.614184	-2.799254	0.000000
27	H	-2.809259	-5.254631	0.000000
28	H	-0.803336	-6.694201	0.000000
29	H	1.461345	-5.681181	0.000000

Table S35. **3b** (S_1): E(B3LYP/def2tzvp) = -3378.48830985 hartree

Number	Atom	X	Y	Z
1	C	-0.8555250	2.7951210	0.0000000
2	C	0.2299040	3.6422980	0.0000000
3	C	-0.5147680	1.4163600	0.0000000
4	C	0.8714820	1.2165980	0.0000000
5	S	1.6977670	2.7579820	0.0000000
6	As	-1.8154790	-0.0000110	0.0000000
7	C	-0.5147560	-1.4163620	0.0000000
8	C	0.8714950	-1.2165900	0.0000000
9	C	1.5719780	0.0000110	0.0000000
10	C	-0.8555250	-2.7951200	0.0000000
11	C	0.2299040	-3.6422910	0.0000000

12	S	1.6977710	-2.7579710	0.0000000
13	H	2.6525810	0.0000120	0.0000000
14	H	0.2377960	4.7191590	0.0000000
15	H	-1.8755610	3.1548510	0.0000000
16	H	-1.8755680	-3.1548310	0.0000000
17	H	0.2377910	-4.7191520	0.0000000

9. NMR Spectra

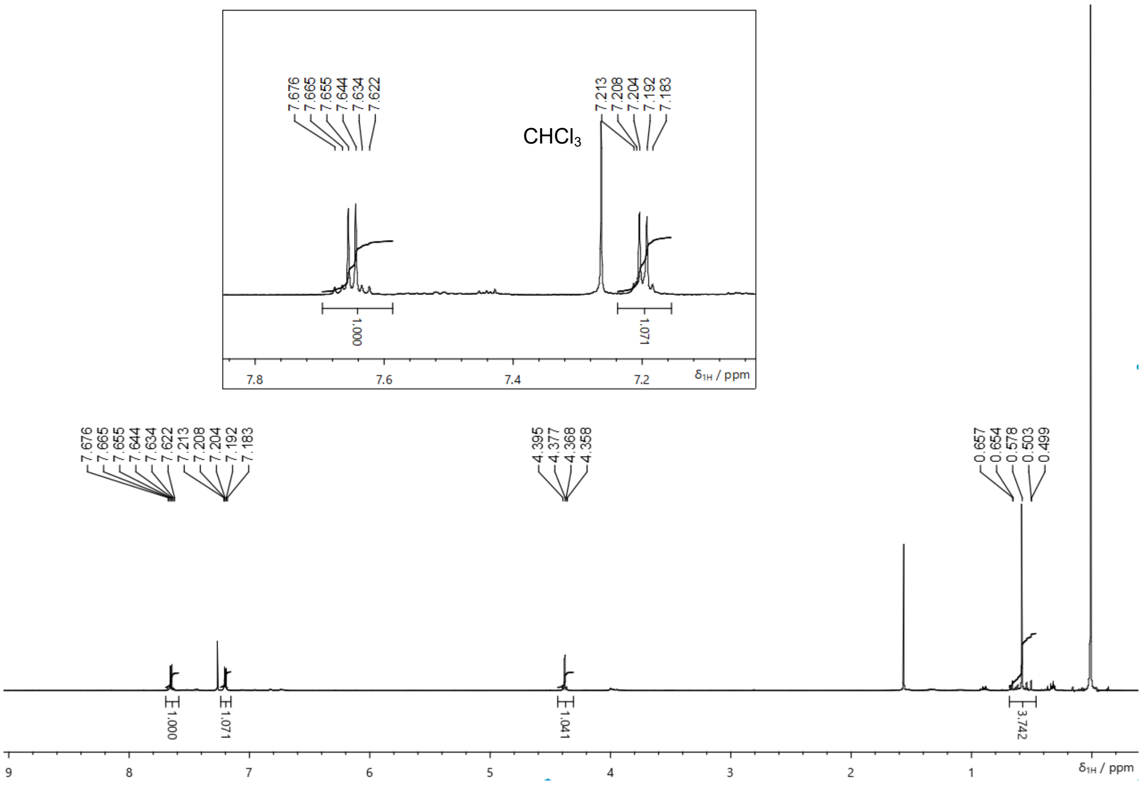

Figure S30. ^{1}H-NMR spectrum (400 MHz) of crude **2a** in $CDCl_3$

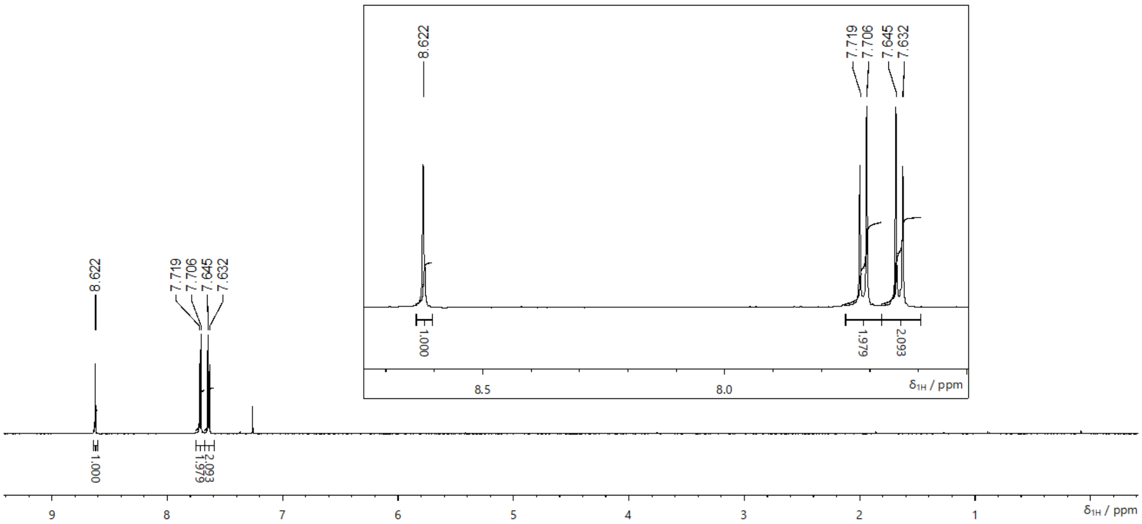

Figure S31. ^{1}H-NMR spectrum (400 MHz) of **3a** in $CDCl_3$.

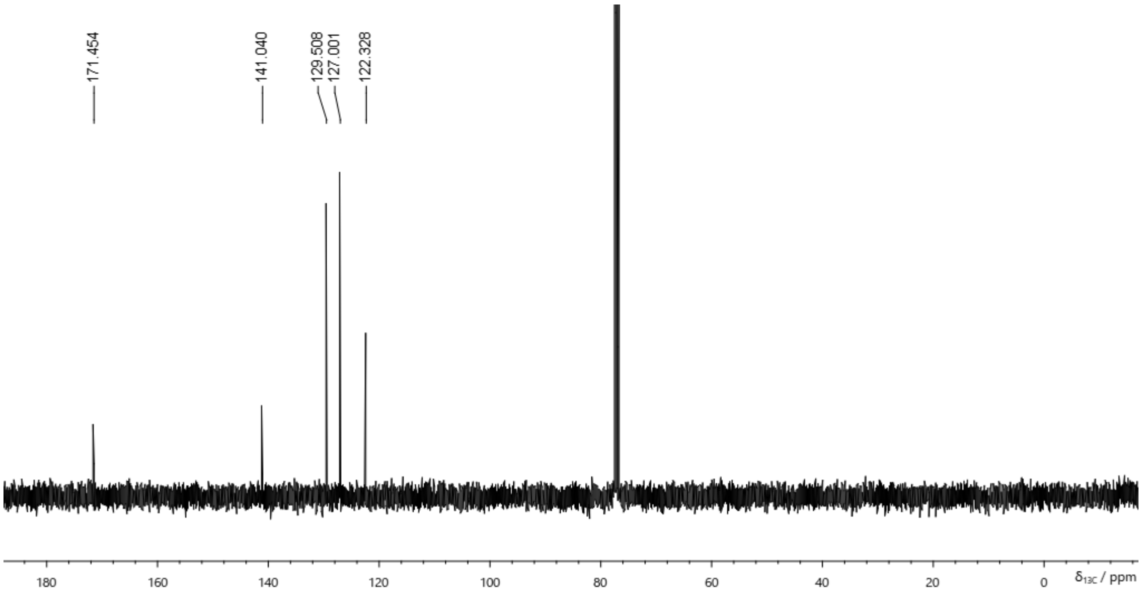

Figure S32. ^{13}C-NMR spectrum (100 MHz) of **3a** in $CDCl_3$.

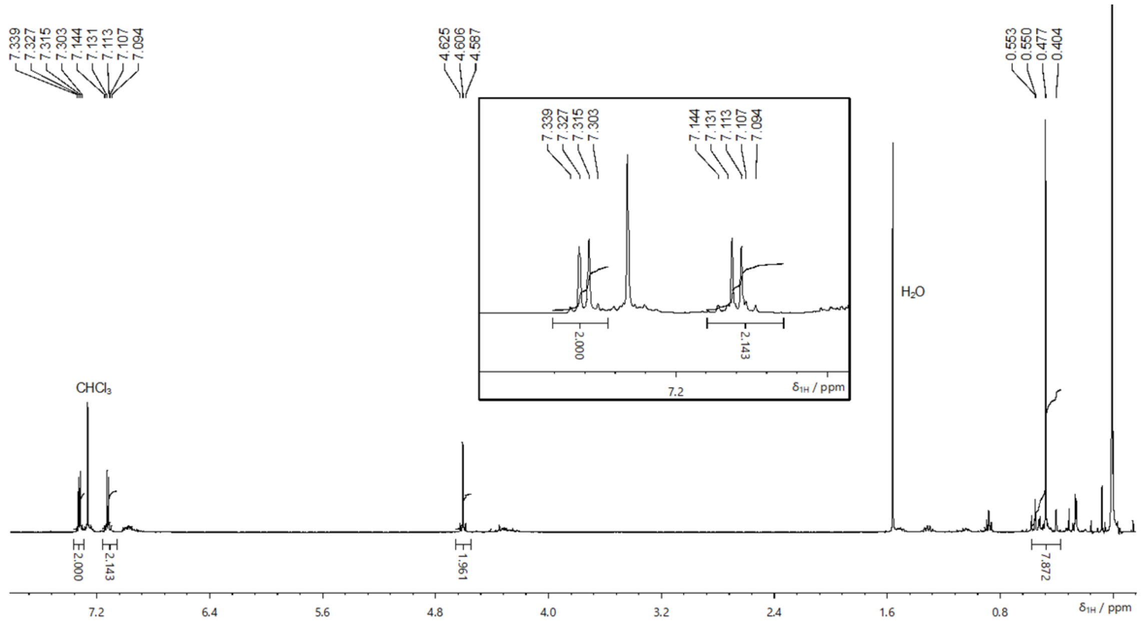

Figure S33. ^{1}H-NMR spectrum (400 MHz) of crude **2b** in $CDCl_3$.

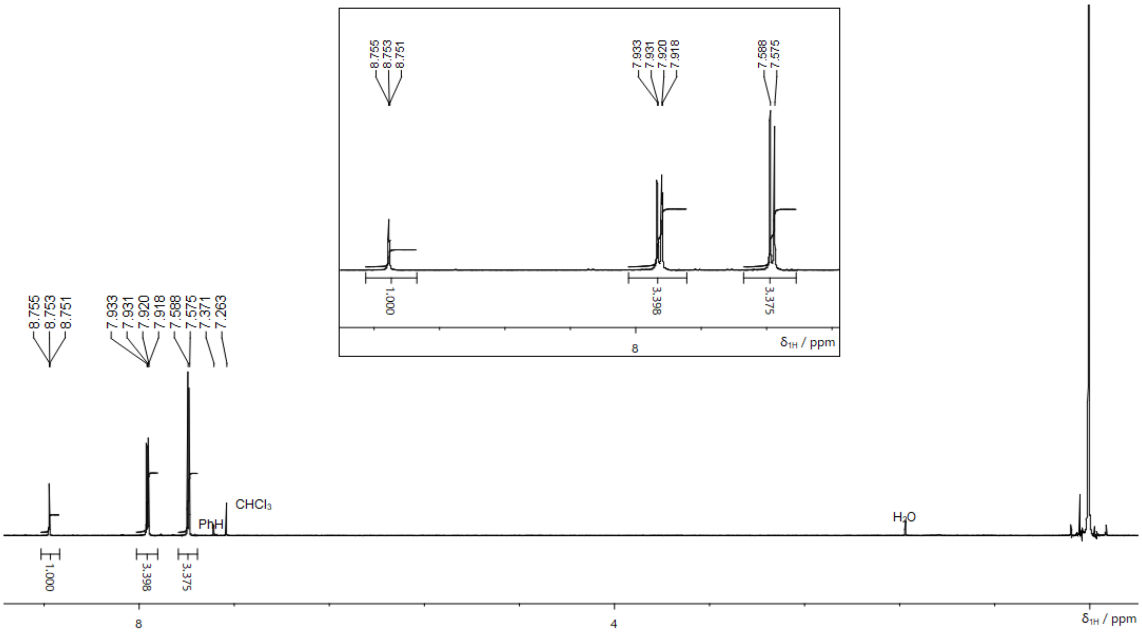

Figure S34. ^{1}H-NMR spectrum (100 MHz) of **3b** in $CDCl_3$.

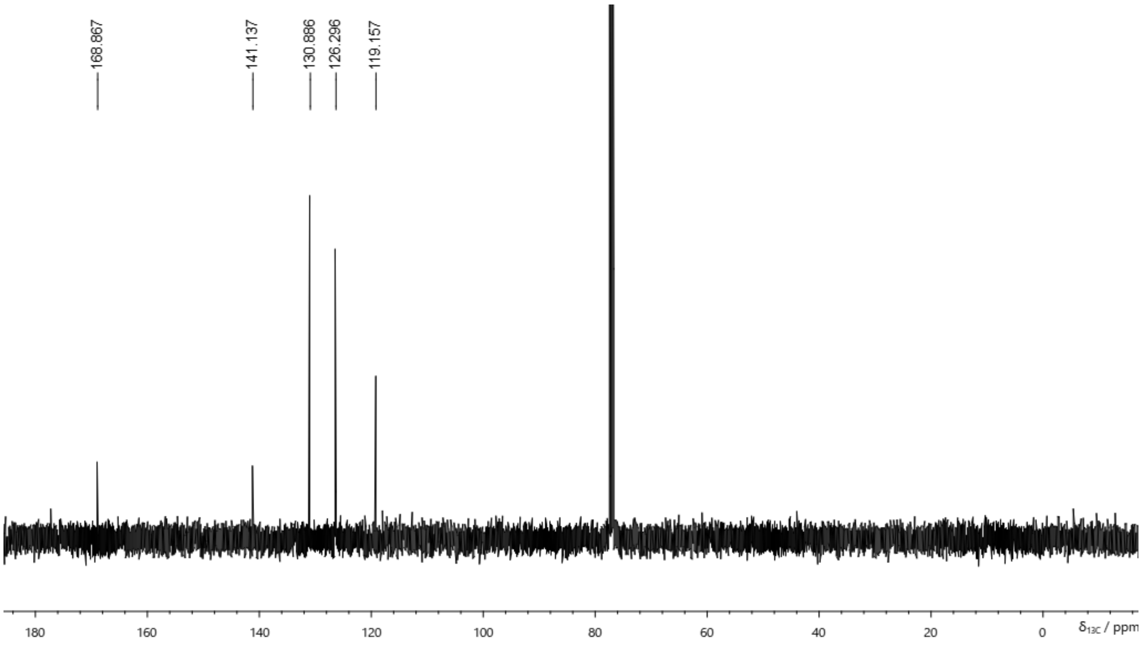

Figure S35. ^{13}C -NMR spectrum (400 MHz) of **3b** in $CDCl_3$.

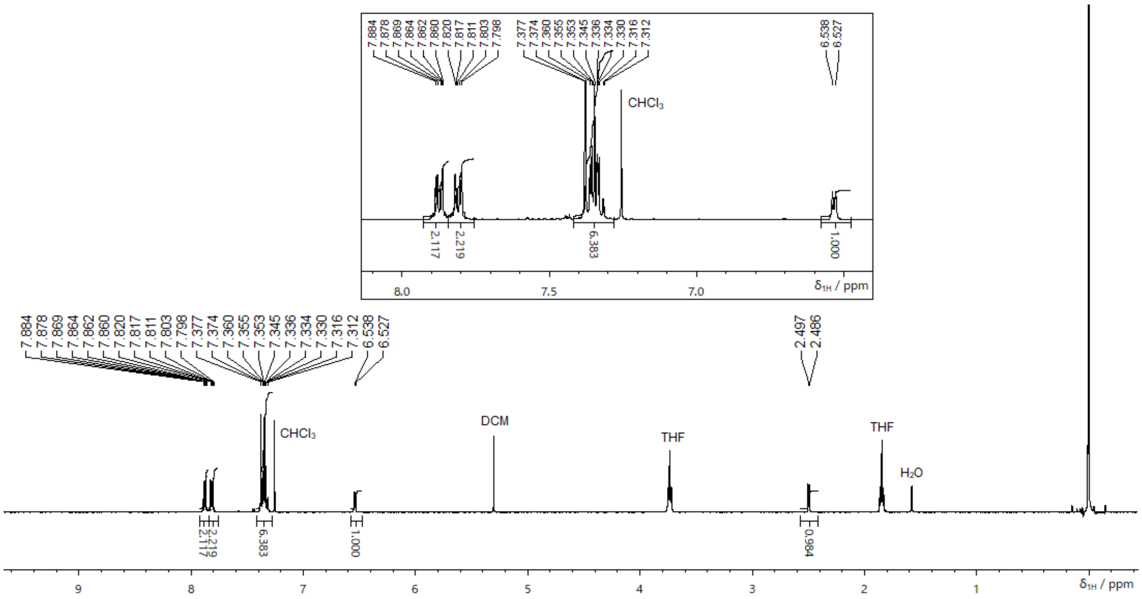

Figure S36. ^{1}H-NMR spectrum (400 MHz) of crude bis-3-benzo[*b*]thienylmethanol in $CDCl_3$.

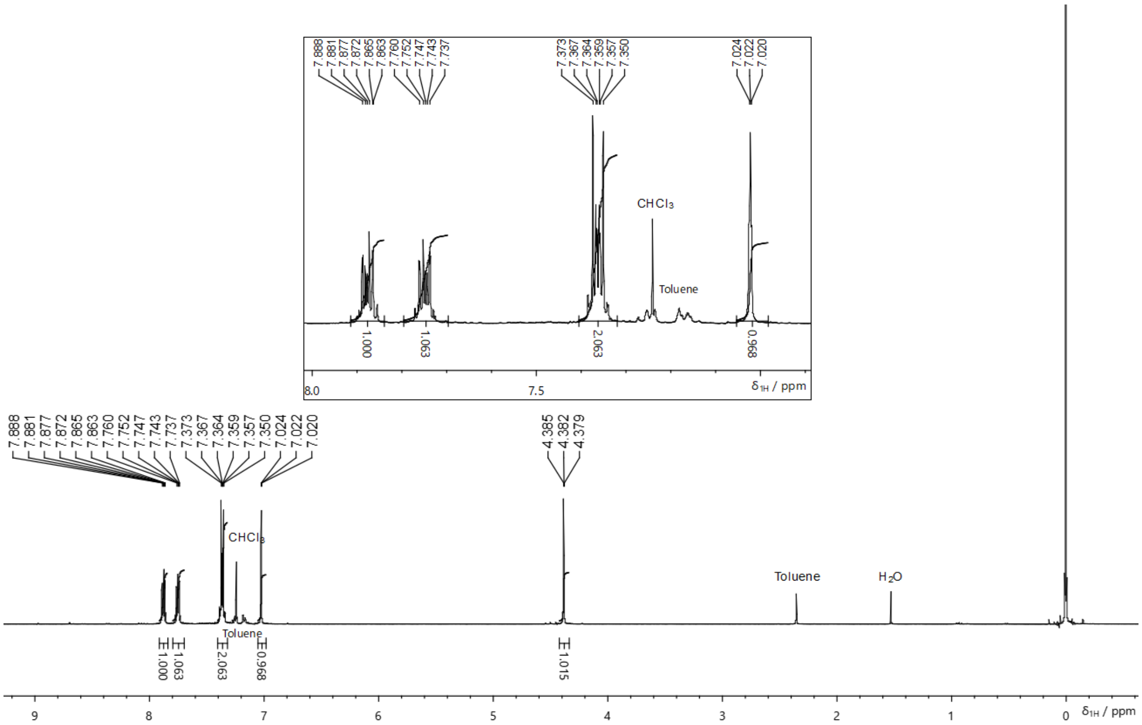

Figure S37. ^{1}H-NMR spectrum (400 MHz) of crude bis-3-benzo[*b*]thienylmethane in $CDCl_3$.

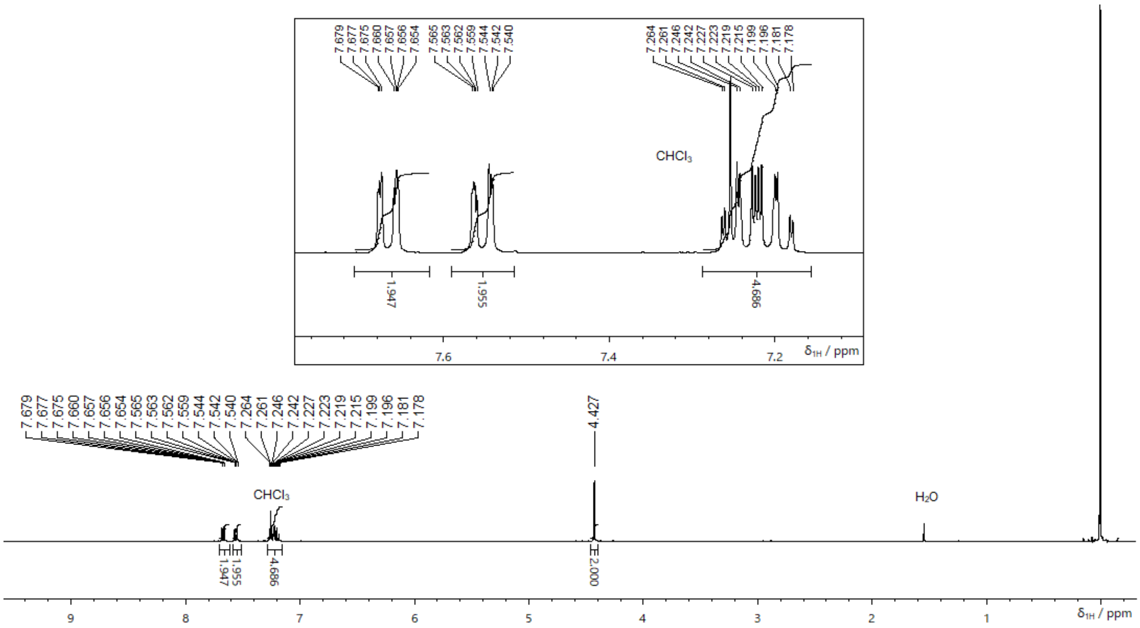

Figure S38. ^{1}H-NMR spectrum (400 MHz) of bis-3-(2-bromobenzo[*b*]thienyl)methane in $CDCl_3$.

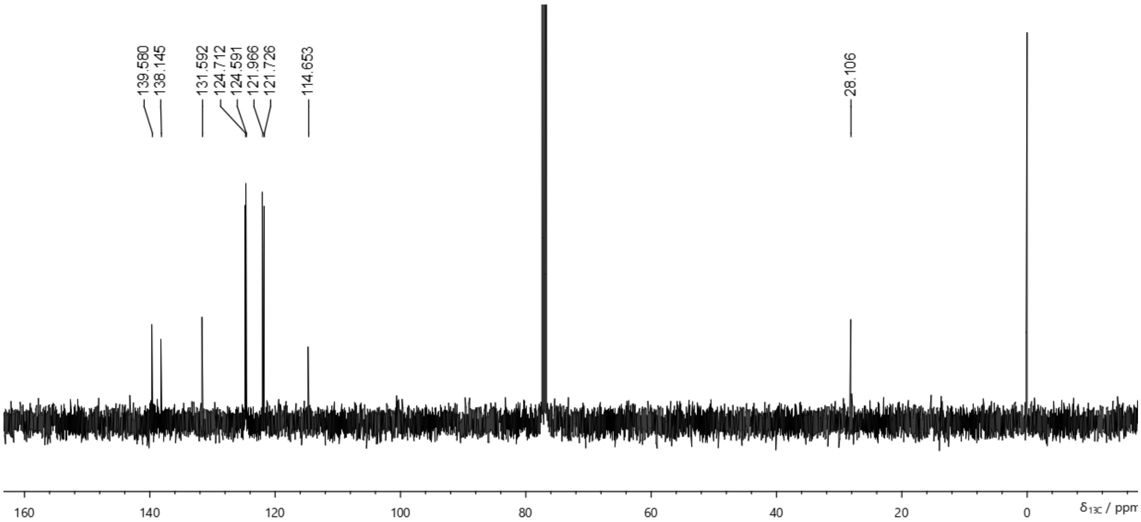

Figure S39. ^{13}C-NMR spectrum (100 MHz) of bis-3-(2-bromobenzo[*b*]thienyl)methane in $CDCl_3$.

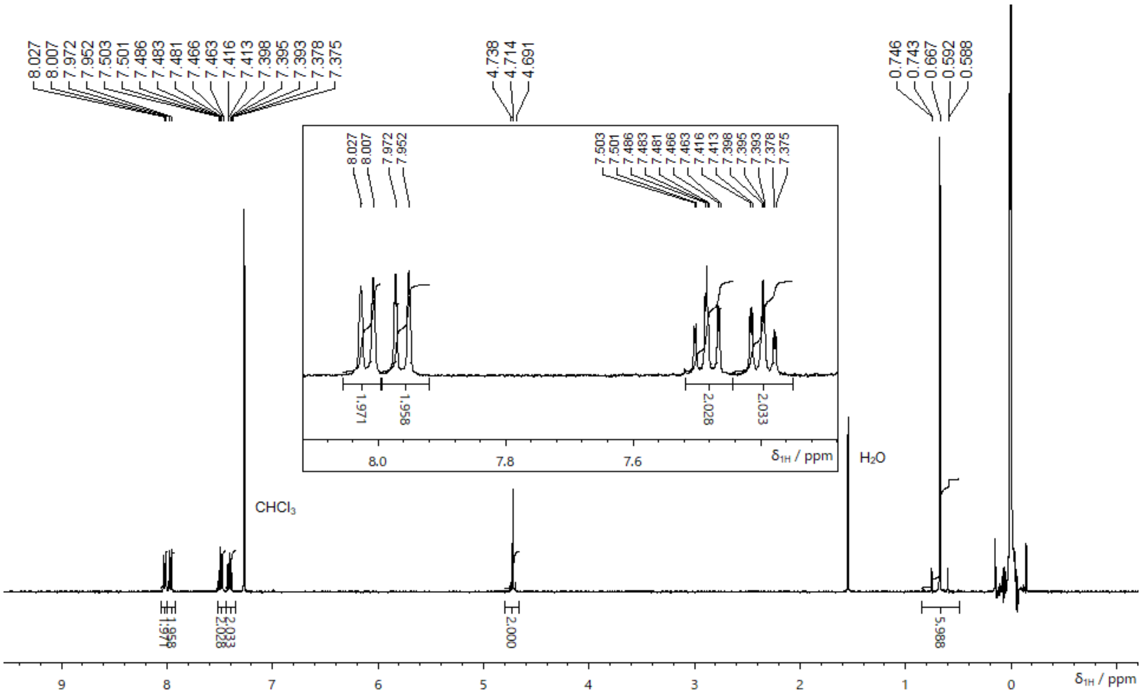

Figure S40. ^{1}H-NMR spectrum (400 MHz) of **2c** in $CDCl_3$.

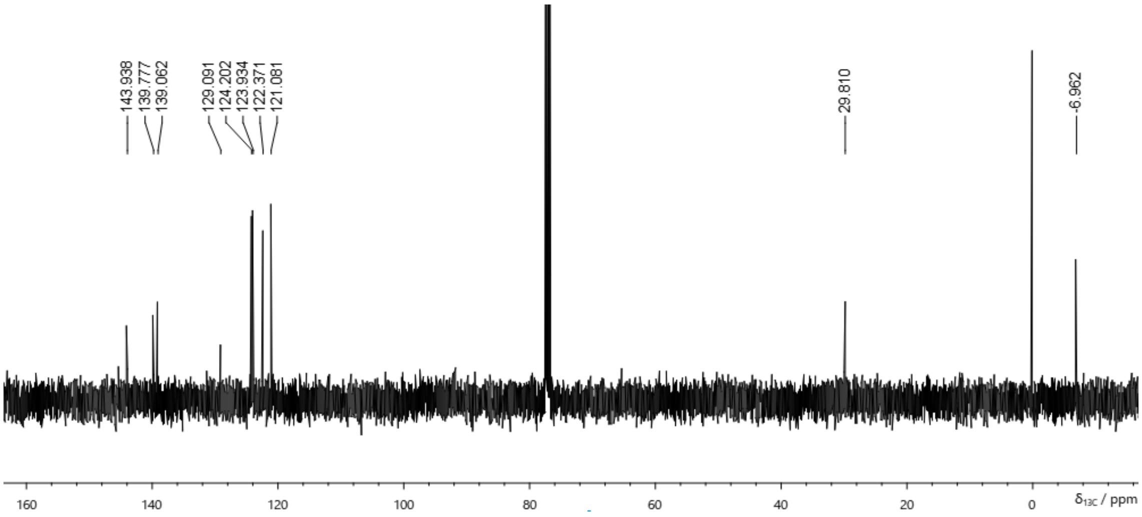

Figure S41. ^{13}C-NMR spectrum (100 MHz) of **2c** in $CDCl_3$.

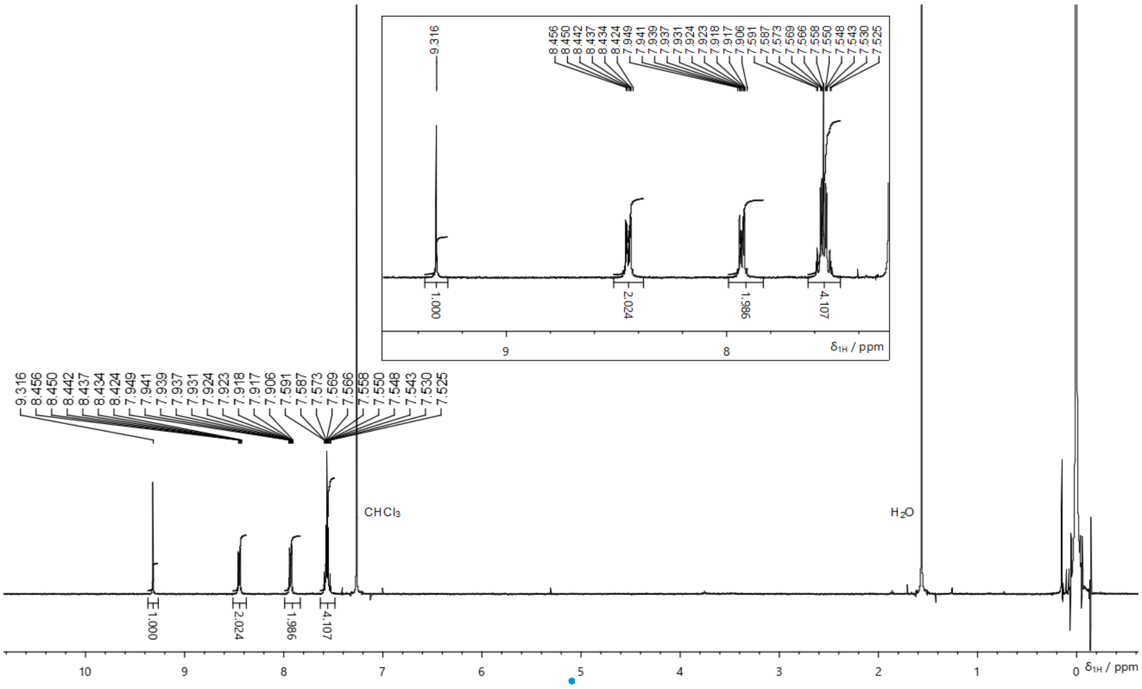

Figure S42. ^{1}H-NMR spectrum (400 MHz) of **3c** in $CDCl_3$.

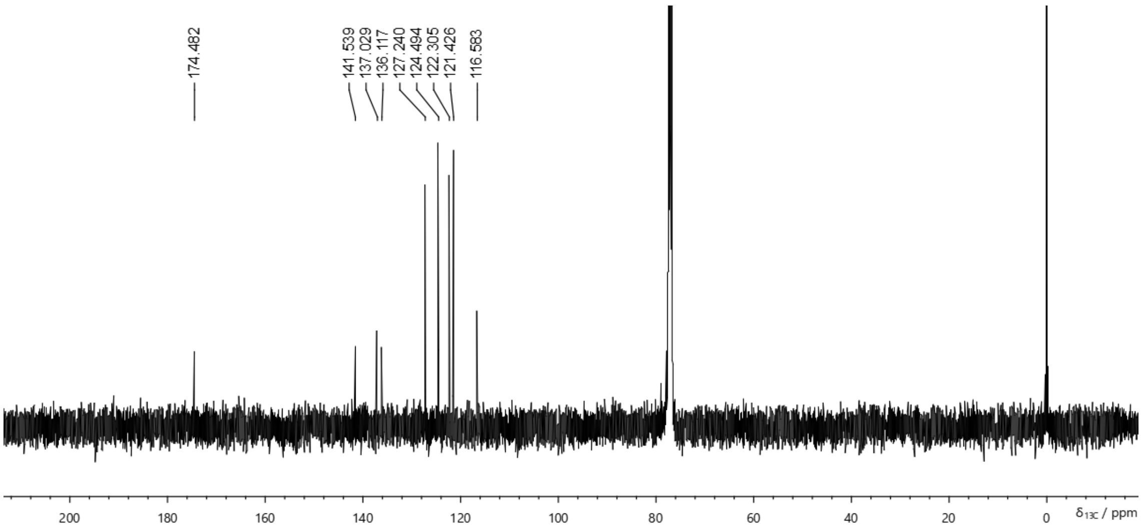

Figure S43. ^{13}C-NMR spectrum (100 MHz) of **3c** in $CDCl_3$.

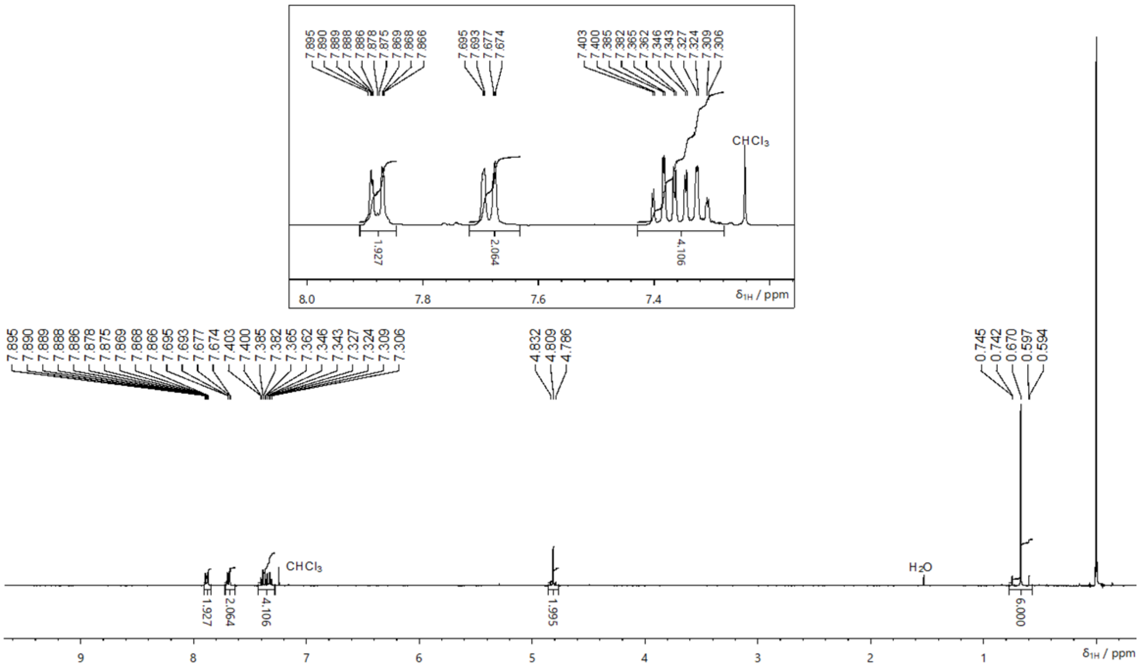

Figure S44. ^{1}H-NMR spectrum (400 MHz) of **2d** in $CDCl_3$.

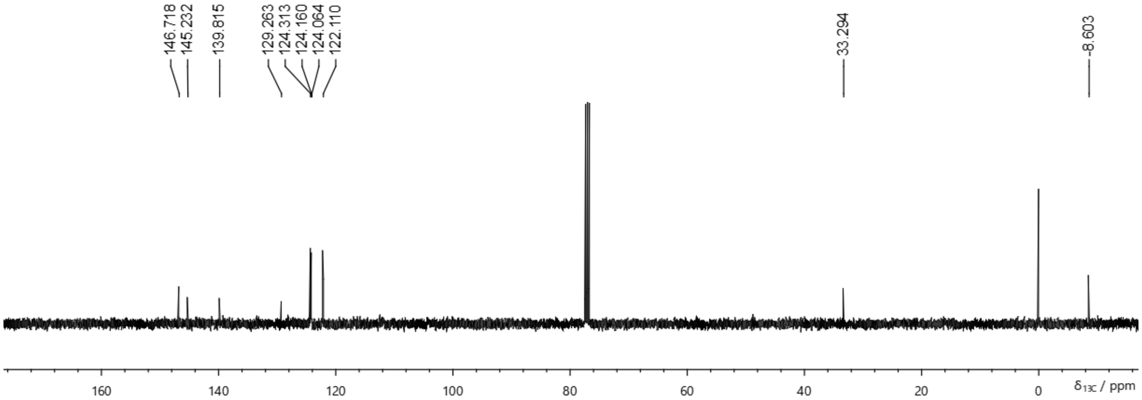

Figure S45. ^{13}C-NMR spectrum (100 MHz) of **2d** in $CDCl_3$.

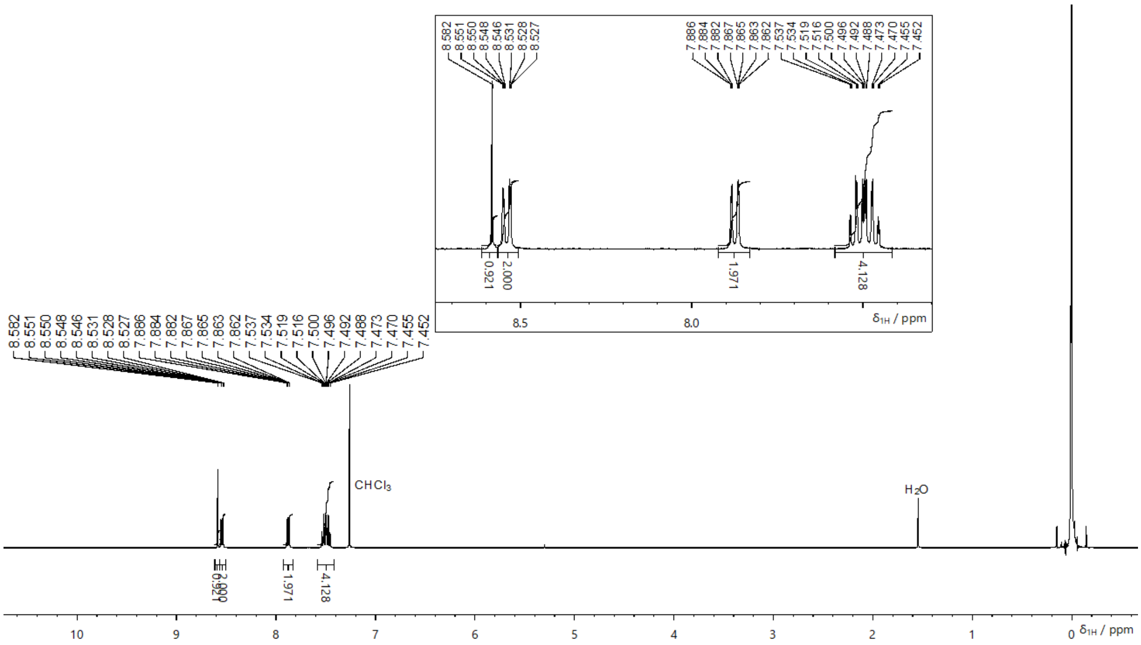

Figure S46. ^{1}H-NMR spectrum (400 MHz) of **3d** in $CDCl_3$.

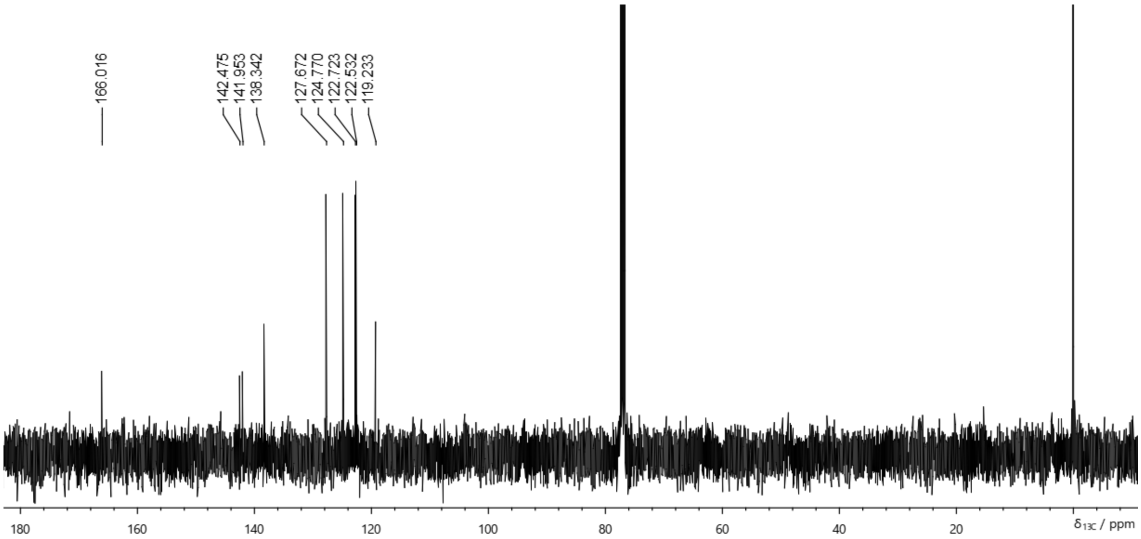

Figure S47. ^{13}C-NMR spectrum (100 MHz) of **3d** in $CDCl_3$

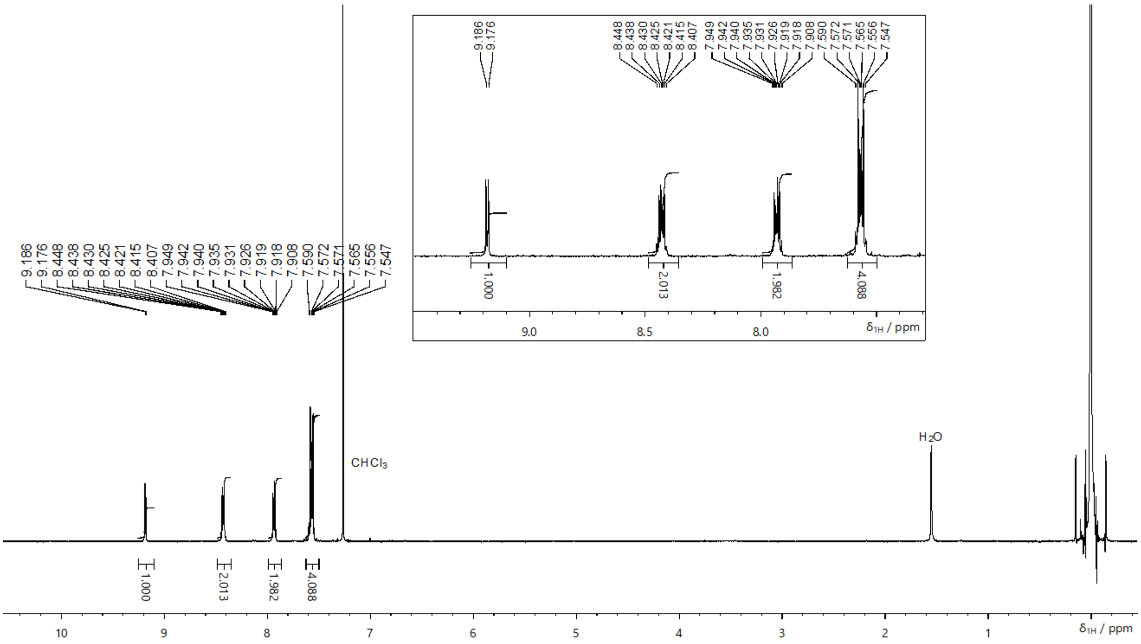

Figure S48. ^{1}H-NMR spectrum (400 MHz) of **3c-P** in $CDCl_3$.

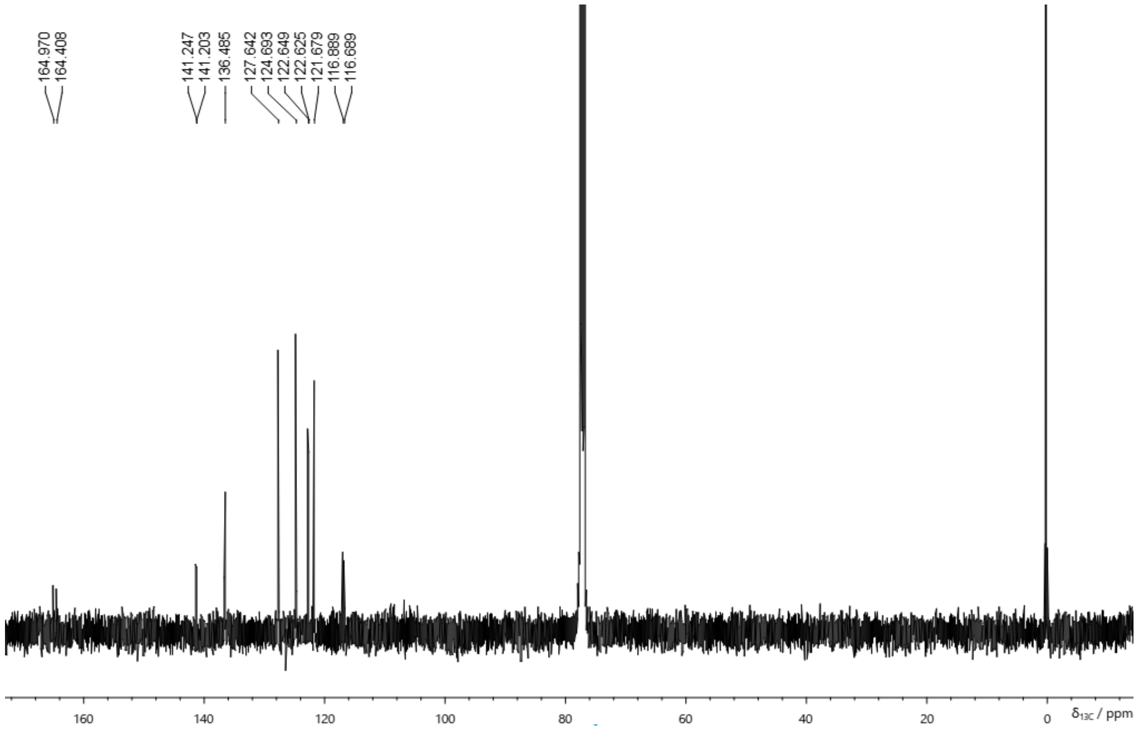

Figure S49. ^{13}C-NMR spectrum (100 MHz) of **3c-P** in $CDCl_3$.

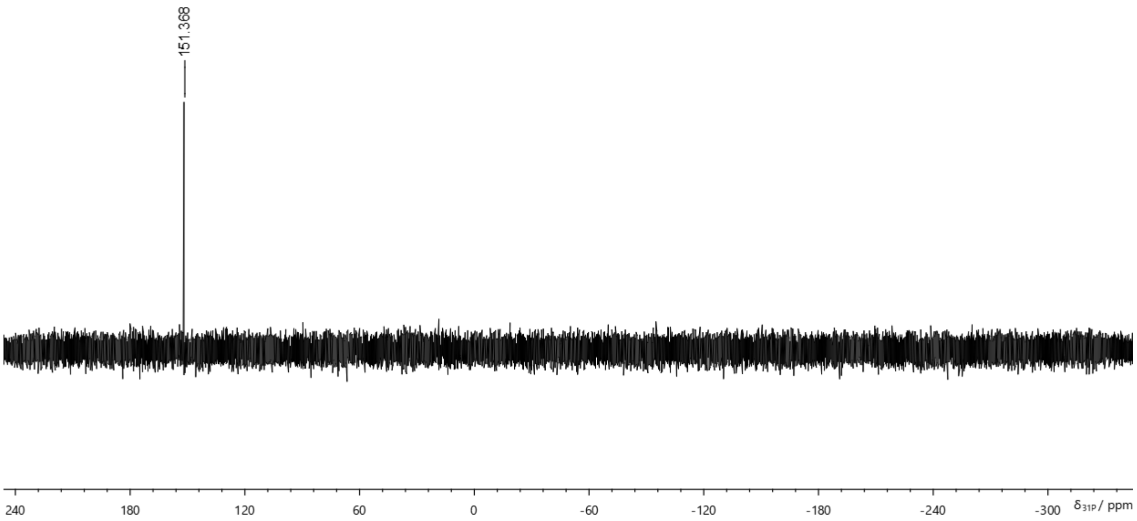

Figure S50. ^{31}P-NMR spectrum (162 MHz) of **3c-P** in $CDCl_3$.

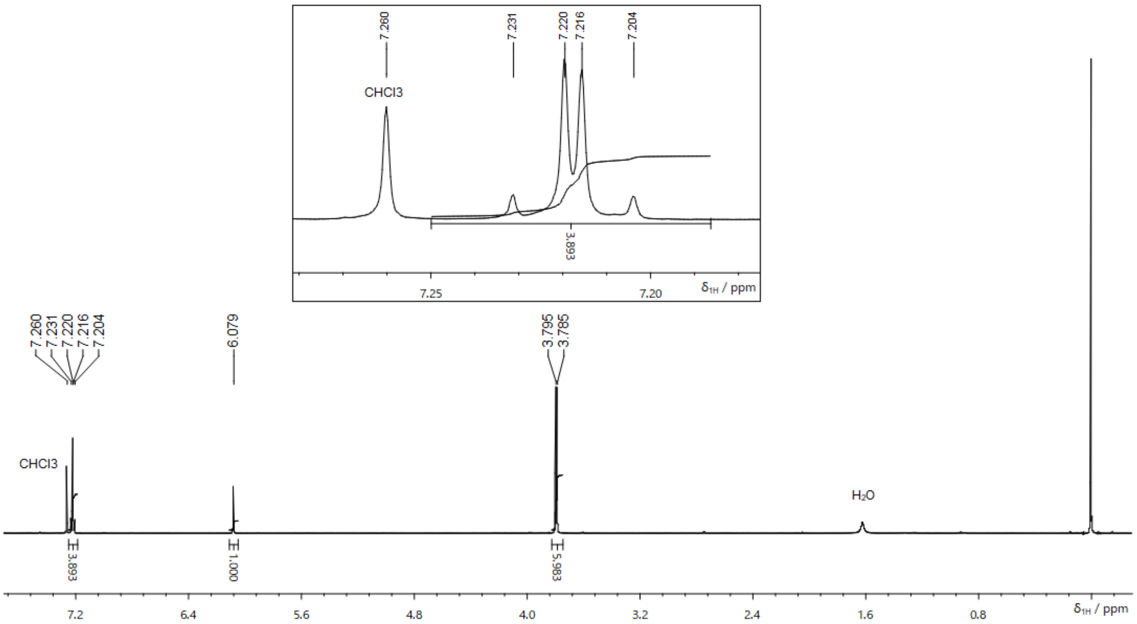

Figure S51. ^{1}H-NMR spectrum (400 MHz) of **3a-DMAD** in $CDCl_3$.

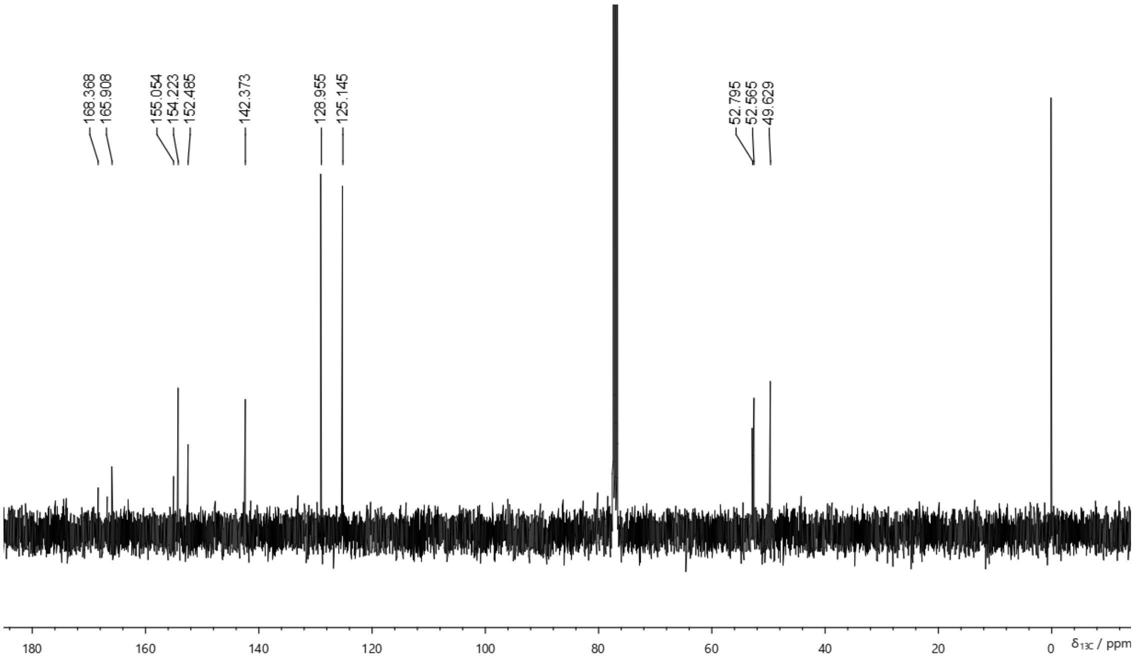

Figure S52. ^{13}C-NMR spectrum (100 MHz) of **3a-DMAD** in $CDCl_3$.

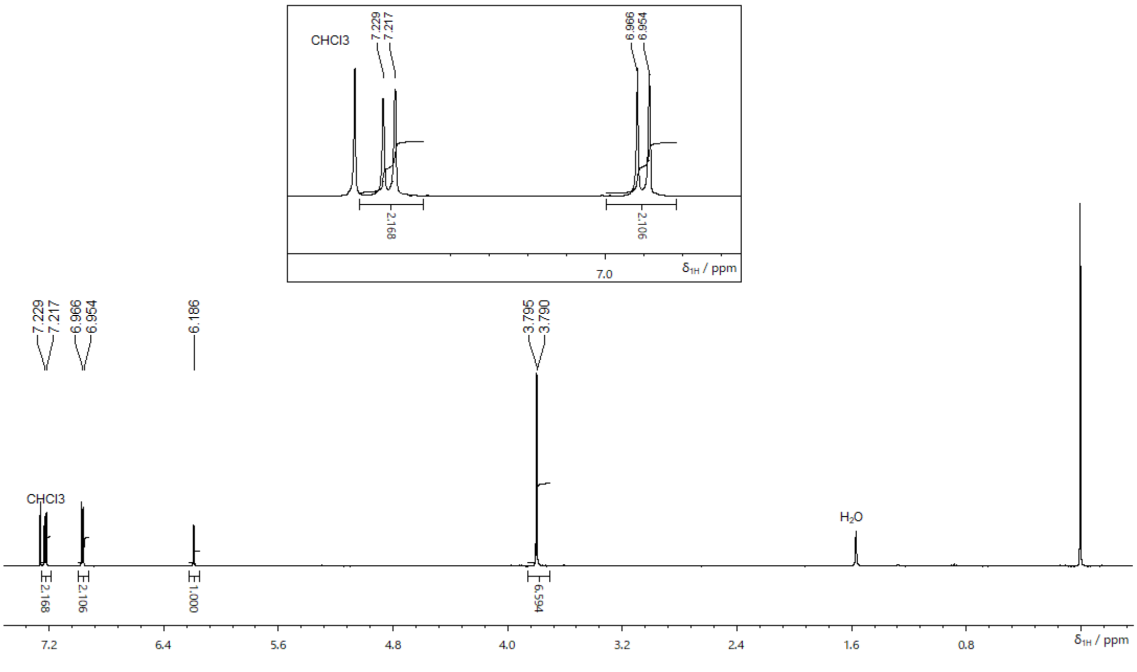

Figure S53. ^{1}H-NMR spectrum (400 MHz) of **3b-DMAD** in $CDCl_3$.

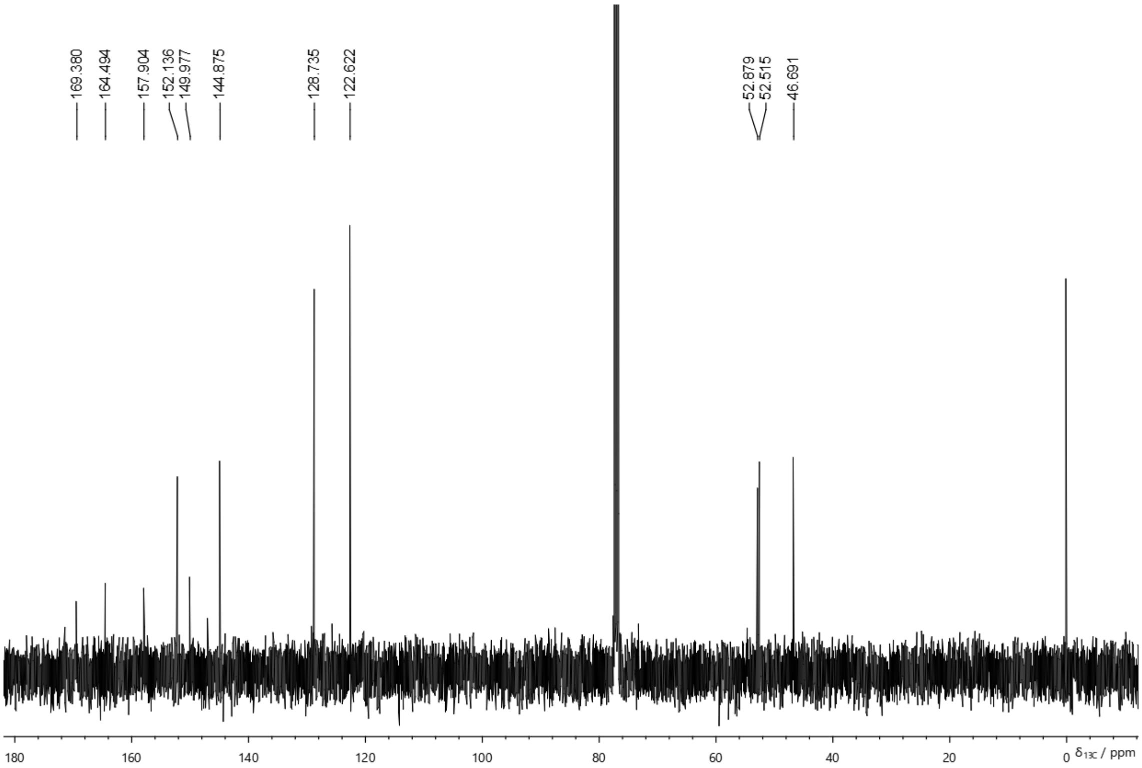

Figure S54. ^{13}C-NMR spectrum (100 MHz) of **3b-DMAD** in $CDCl_3$.

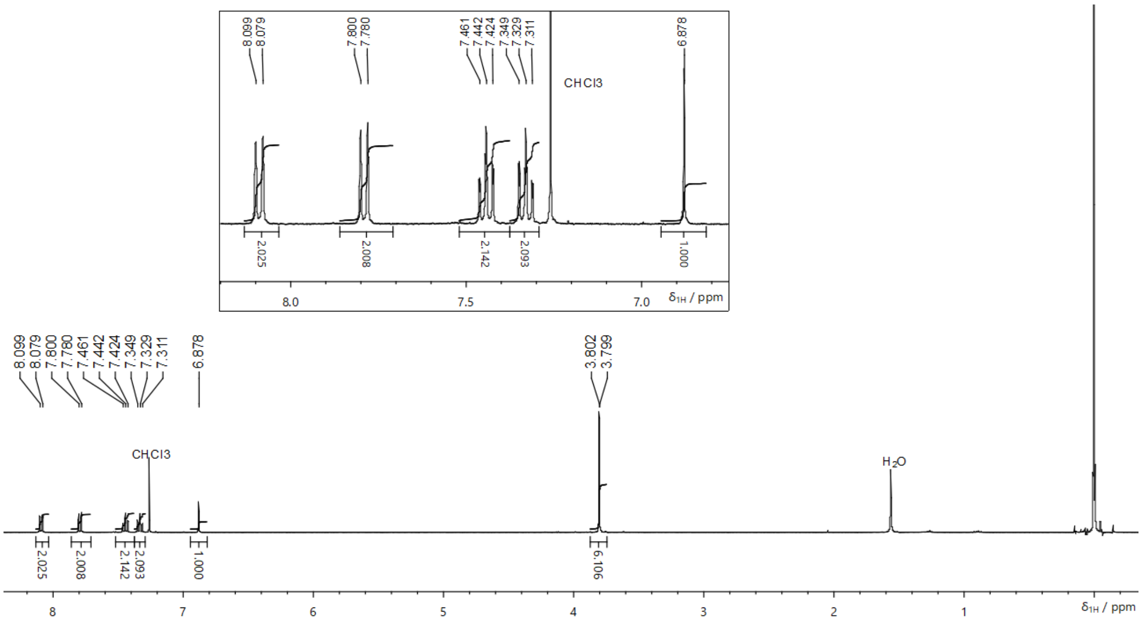

Figure S55. ^{1}H-NMR spectrum (400 MHz) of **3c-DMAD** in $CDCl_3$.

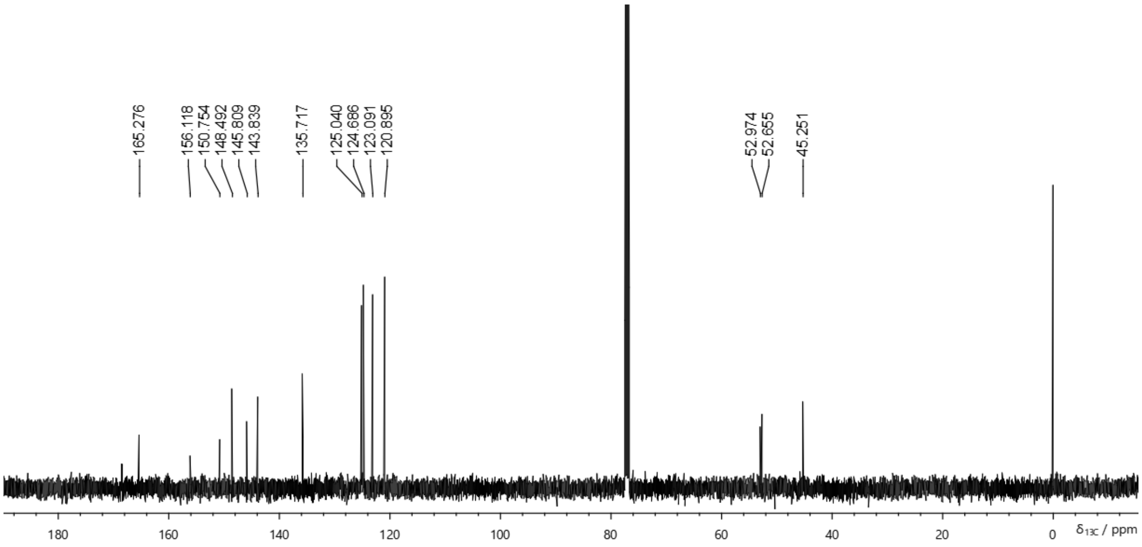

Figure S56. ^{13}C-NMR spectrum (100 MHz) of **3c-DMAD** in $CDCl_3$.

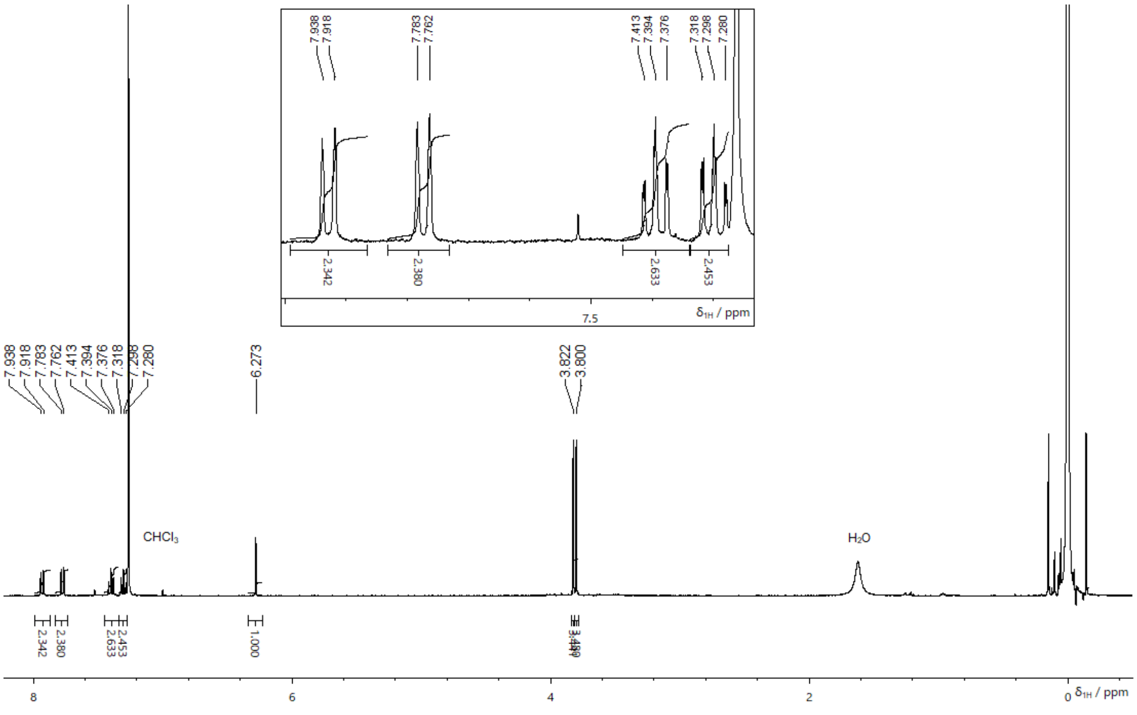

Figure S57. ^{1}H-NMR spectrum (400 MHz) of **3d-DMAD** in $CDCl_3$.

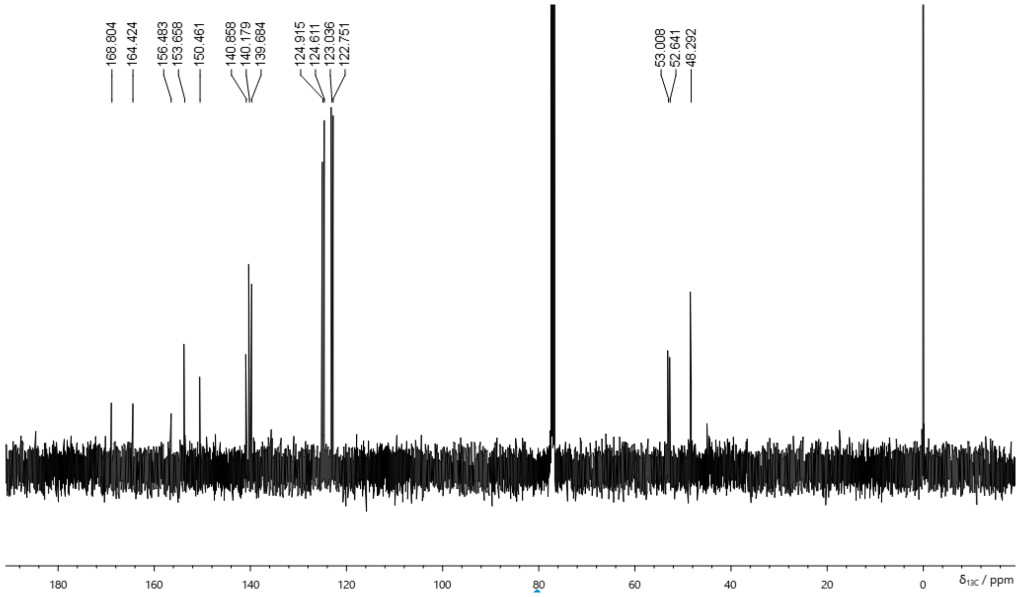

Figure S58. ^{13}C-NMR spectrum (100 MHz) of **3d-DMAD** in $CDCl_3$.

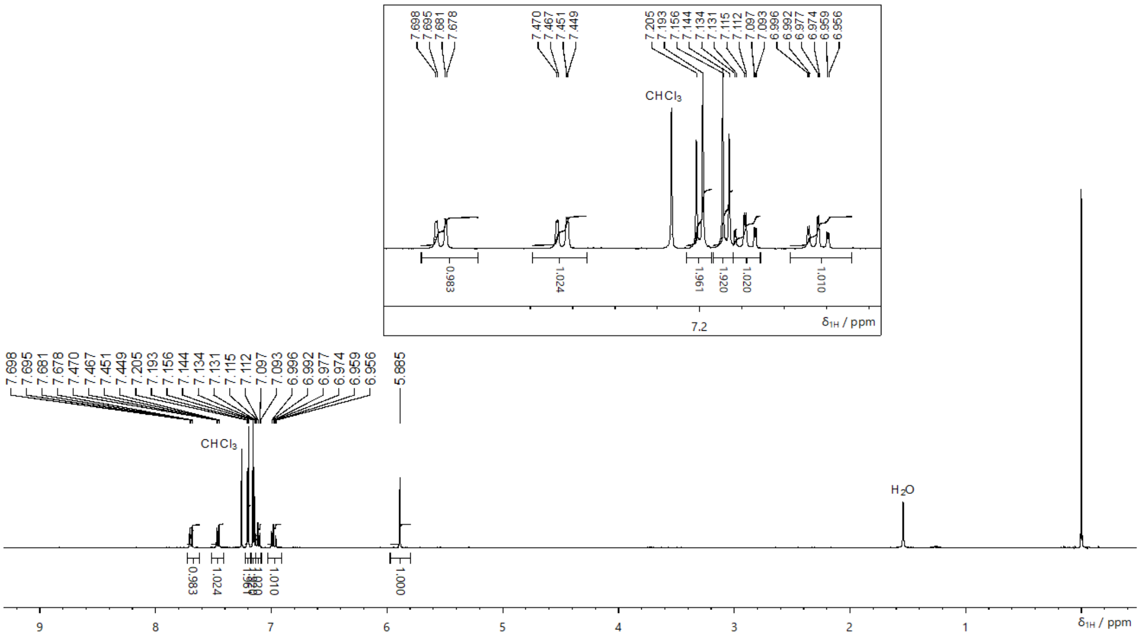

Figure S59. ^{1}H-NMR spectrum (400 MHz) of **3a-benzyne** in $CDCl_3$.

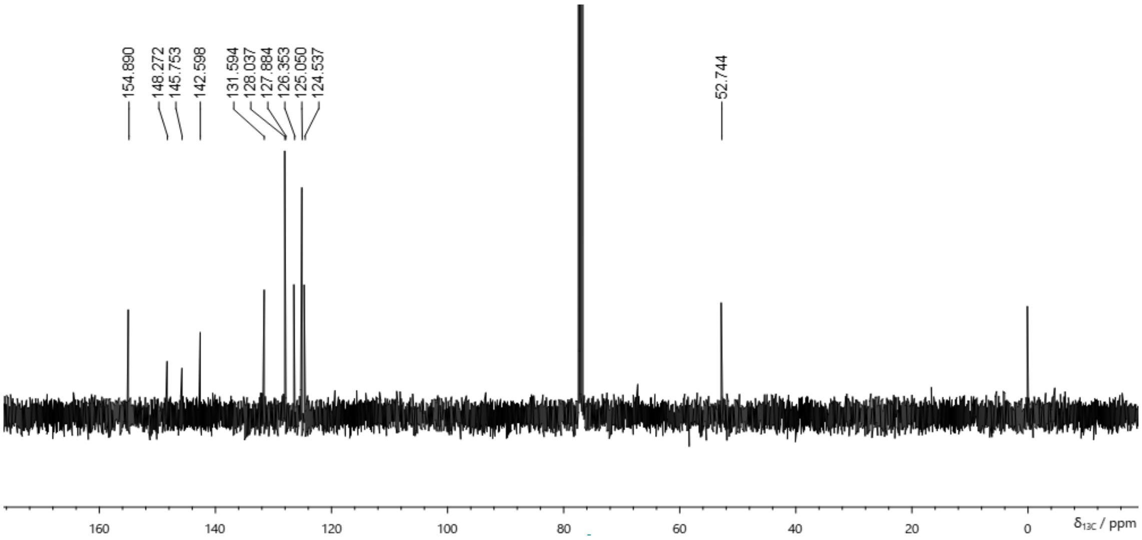

Figure S60. ^{13}C-NMR spectrum (100 MHz) of **3a-benzyne** in $CDCl_3$.

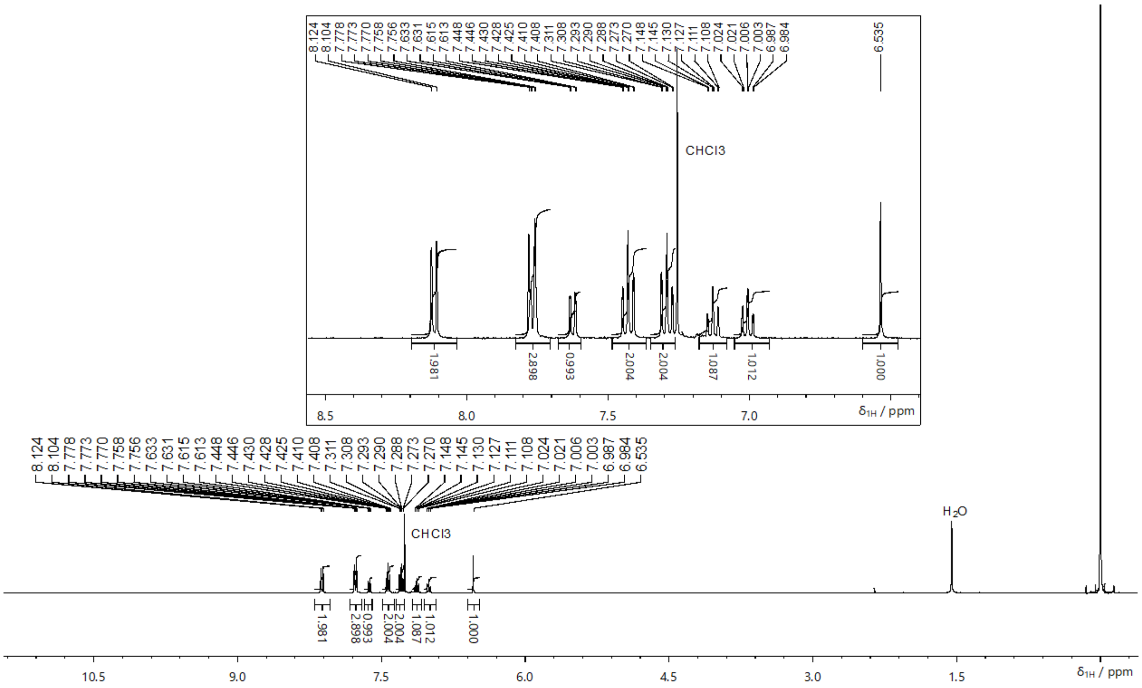

Figure S61. ^{1}H-NMR spectrum (400 MHz) of **3c-benzyne** in $CDCl_3$.

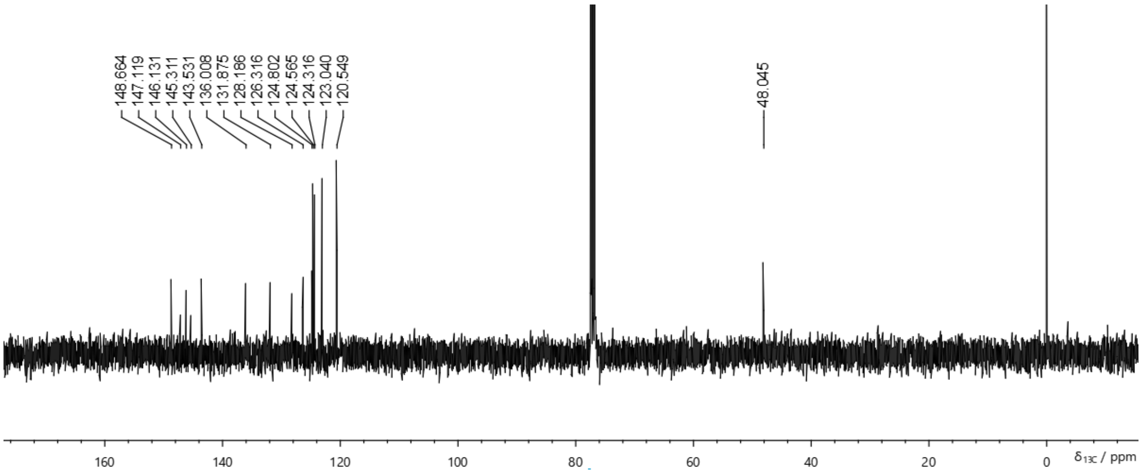

Figure S62. ^{13}C-NMR spectrum (100 MHz) of **3c-benzyne** in $CDCl_3$.

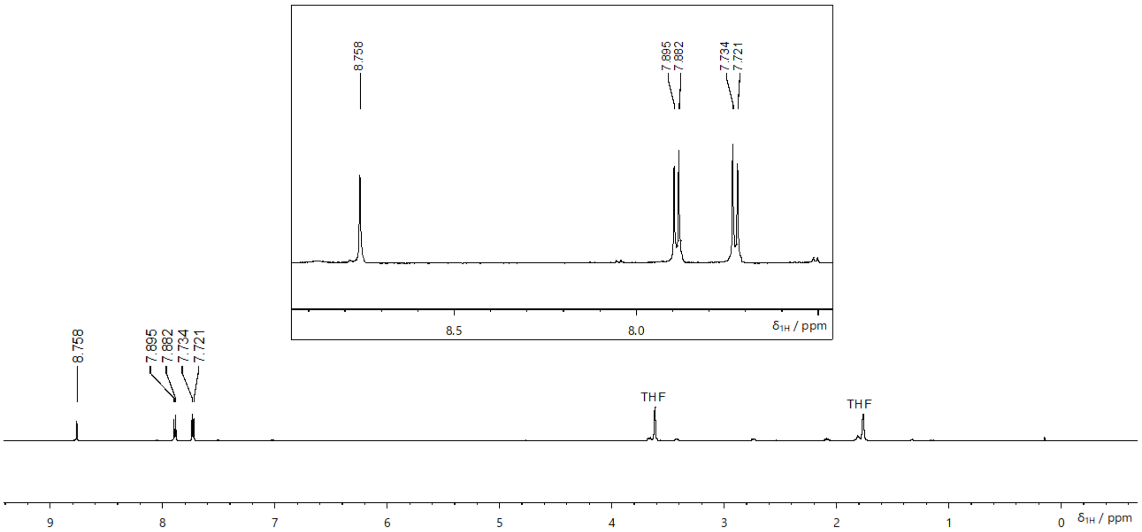

Figure S63. ^{1}H-NMR spectrum (400 MHz) of **3a-W(CO)$_5$** in $CDCl_3$. Minor impurities (1-4 ppm) were attributed to atmospheric solvent in the glove box.

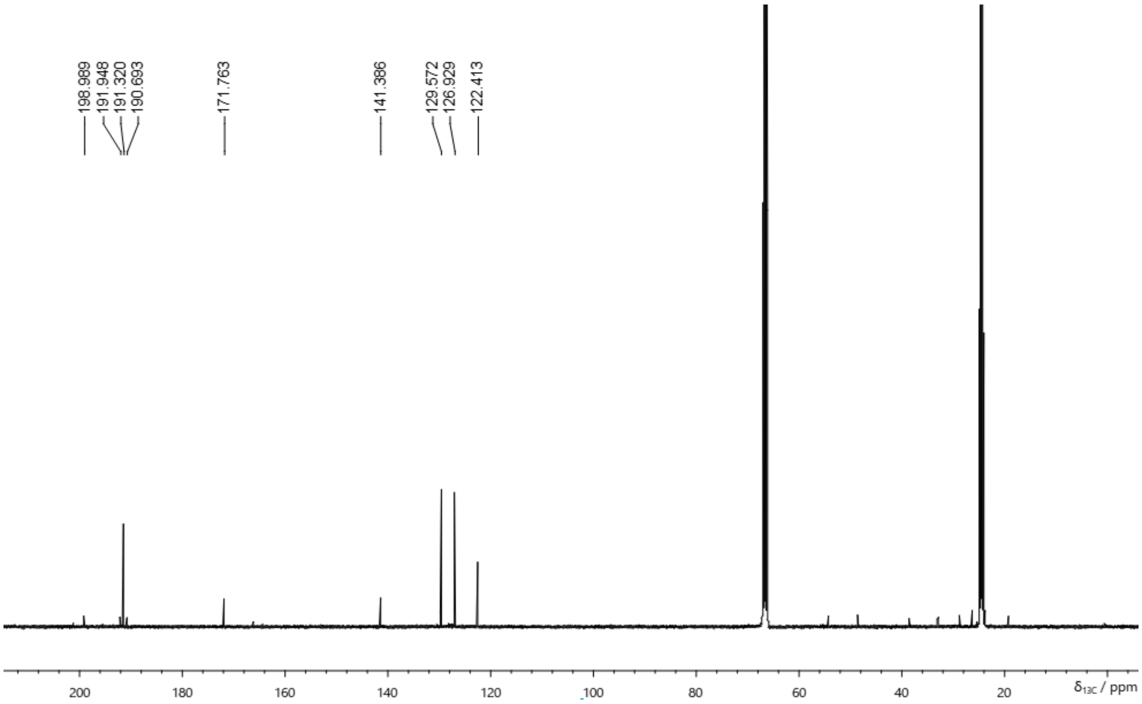

Figure S64. ^{13}C-NMR spectrum (100 MHz) of **3a-W(CO)$_5$** in $CDCl_3$. Minor impurities (16-56 ppm) were attributed to atmospheric solvent in the glove box.

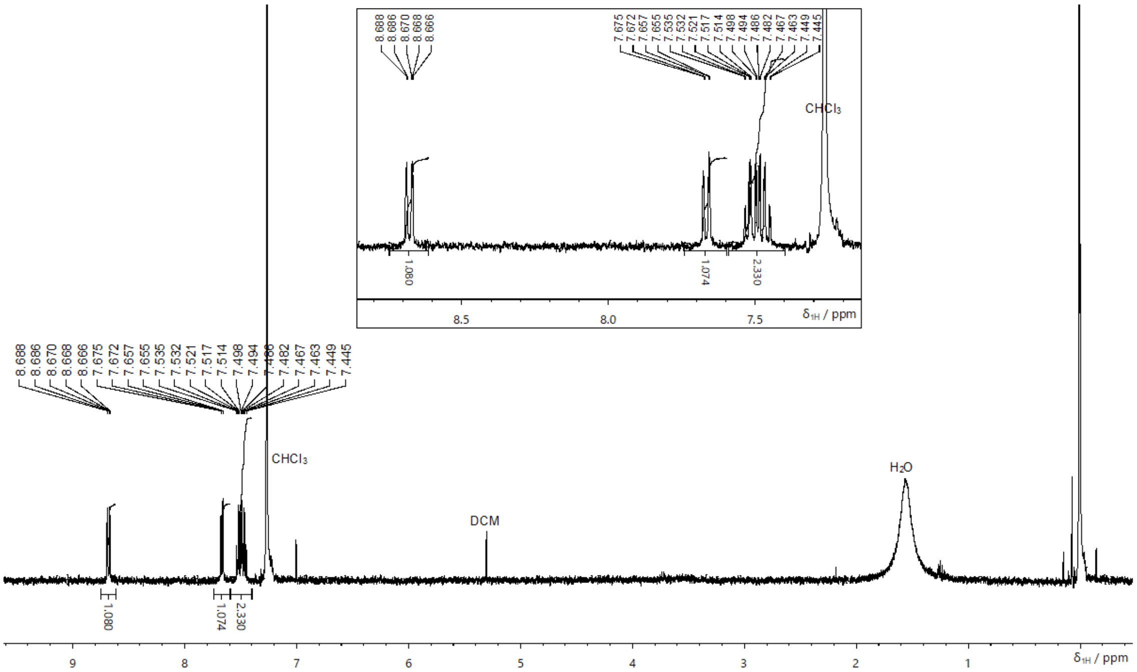

Figure S65. ^{1}H-NMR spectrum (400 MHz) of **3d’** in $CDCl_3$.

Reference

[1] (a) H. Wynberg, A. Kraak, *J. Org. Chem.* **1964**, *29*, 2455. (b) A. Kraak, A. K. Wiersema, P. Jordens, H. Wynberg, *Tetrahedron* **1968**, *24*, 3381.

[2] J. C. Bijleveld, M. Shahid, J. Gilot, M. M. Wienk, R. A. J. Janssen, *Adv. Funct. Mater.* **2009**, *19*, 3262.

[3] S. Tanaka, M. Konishi, H. Imoto, Y. Nakamura, M. Ishida, H. Furuta, K. Naka, *Inorg. Chem*. **2020**, *59*, 9587.

[4] L. E. Doyle, W. E. Piers, J. B-. Garcia, *J. Am. Chem. Soc.* **2015**, *137*, 2187.

[5] K. Matsuo, R. Okumura, H. Hayashi, N. Aratani, S. Jinnai, Y. Ie, A. Saeki, H. Yamada, *Chem. Commun.* **2022**, *58*, 13576.

[6] *CrysAlisPro Software System, Rigaku Oxford Diffraction* **2020**.

[7] R. C. Clark, J. S. Reid, Analytical numerical absorption correction multi-faceted crystal model, *Acta Crystallogr., Sect. A: Found. Crystallogr.* **1995**, *51*, 887.

[8] G. M. Sheldrick, *SHELXT, Acta Crystallogr., Sect. C: Struct. Chem.* **2015**, *A71*, 3.

[9] O. V. Dolomanov, L. J. Bourhis, R. J. Gildea, J. A. K. Howard, H. J. Puschmann, *Olex2 J. Appl. Crystallogr.* **2009**, *42*, 339.

[10] G. M. Sheldrick, *SHELXL2016, Acta Crystallogr., Sect. C: Struct. Chem.* **2015**, *C71*, 3.

[11] Gaussian 16, Revision C.01, M. J. Frisch, G. W. Trucks, H. B. Schlegel, G. E. Scuseria, M. A. Robb, J. R. Cheeseman, G. Scalmani, V. Barone, G. A. Petersson, H. Nakatsuji, X. Li, M. Caricato, A. V. Marenich, J. Bloino, B. G. Janesko, R. Gomperts, B. Mennucci, H. P. Hratchian, J. V. Ortiz, A. F. Izmaylov, J. L. Sonnenberg, D. Williams-Young, F. Ding, F. Lipparini, F. Egidi, J. Goings, B. Peng, A. Petrone, T. Henderson, D. Ranasinghe, V. G. Zakrzewski, J. Gao, N. Rega, G. Zheng, W. Liang, M. Hada, M. Ehara, K. Toyota, R. Fukuda, J. Hasegawa, M. Ishida, T. Nakajima, Y. Honda, O. Kitao, H. Nakai, T. Vreven, K. Throssell, J. A. Montgomery, Jr., J. E. Peralta, F. Ogliaro, M. J. Bearpark, J. J. Heyd, E. N. Brothers, K. N. Kudin, V. N. Staroverov, T. A. Keith, R. Kobayashi, J. Normand, K. Raghavachari, A. P. Rendell, J. C. Burant, S. S. Iyengar, J. Tomasi, M. Cossi, J. M. Millam, M. Klene, C. Adamo, R. Cammi, J. W. Ochterski, R. L. Martin, K. Morokuma, O. Farkas, J. B. Foresman and D. J. Fox, Gaussian, Inc., Wallingford CT, **2016**.

[12] A. E. Reed, L. A. Curtiss, F. Weinhold, *Chem. Rev*. **1988**, *88*, 899.

[13] F. Jensen, *J. Chem. Theory Comput.* **2015**, *11*, 132.

[14] R. G-. Poranne, A. Stanger, *Chem. Eur. J.* **2014**, *20*, 5673.

[15] Z. Wang, py.Aroma 3. https://wongzit.github.io/program/pyaroma

[16] R. Herges, D. Geuenich, *J. Phys. Chem. A* **2001**, *105*, 3214.

[17] (a) R. A. Boto, F. Peccati, R. Laplaza, C. Quan, A. Carbone, J.-P. Piquemal, Y. Maday, J. Contreras-García, *J. Chem. Theory Comput.* **2020**, *16*, 4150. (b) E. R. Johnson, S. Keinan, P. Mori-Sánchez, J. Contreras-García, A. J. Cohen, W. Yang, *J. Am. Chem. Soc.* **2010**, *132*, 6498.

[18] T. Lu, F. Chen, *J. Comput. Chem.* **2012**, *33*, 580

[19] VMD ver. 1.9.4a53 W. Humphrey, A. Dalke, K. Schulten, *J. Mol. Graphics* **1996**, *14*, 33.

[20] ADF2023, SCM, Theoretical Chemistry. Vrije Universiteit, Amsterdam, The Netherlands, http://www.scm.com.
